# Human interpretable grammar encodes multicellular systems biology models to democratize virtual cell laboratories

**DOI:** 10.1016/j.cell.2025.06.048

**Published:** 2025-07-26

**Authors:** Jeanette A.I. Johnson, Daniel R. Bergman, Heber L. Rocha, David L. Zhou, Eric Cramer, Ian C. Mclean, Yoseph W. Dance, Max Booth, Zachary Nicholas, Tamara Lopez-Vidal, Atul Deshpande, Randy Heiland, Elmar Bucher, Fatemeh Shojaeian, Matthew Dunworth, André Forjaz, Michael Getz, Inês Godet, Furkan Kurtoglu, Melissa Lyman, John Metzcar, Jacob T. Mitchell, Andrew Raddatz, Jacobo Solorzano, Aneequa Sundus, Yafei Wang, David G. DeNardo, Andrew J. Ewald, Daniele M. Gilkes, Luciane T. Kagohara, Ashley L. Kiemen, Elizabeth D. Thompson, Denis Wirtz, Laura D. Wood, Pei-Hsun Wu, Neeha Zaidi, Lei Zheng, Jacquelyn W. Zimmerman, Jude M. Phillip, Elizabeth M. Jaffee, Joe W. Gray, Lisa M. Coussens, Young Hwan Chang, Laura M. Heiser, Genevieve L. Stein-O’Brien, Elana J. Fertig, Paul Macklin

**Affiliations:** 1 Department of Oncology, Sidney Kimmel Comprehensive Cancer Center, Johns Hopkins University, Baltimore, MD, USA; 2 Convergence Institute, Johns Hopkins University, Baltimore, MD, USA; 3 Department of Pharmacology, Physiology, and Drug Development, University of Maryland School of Medicine, Baltimore, MD, USA; 4 Institute for Genome Sciences, University of Maryland School of Medicine, Baltimore, MD, USA; 5 Marlene & Stuart Greenbaum Comprehensive Cancer Center, University of Maryland School of Medicine, Baltimore, MD, USA; 6 University of Maryland Institute of Health Computing, University of Maryland School of Medicine, Baltimore, MD, USA; 7 Department of Intelligent Systems Engineering, Indiana University, Bloomington, IN, USA; 8 Department of Neuroscience, Johns Hopkins University, Baltimore, MD, USA; 9 Department of Biomedical Engineering, Oregon Health & Science University, Portland, OR, USA; 10 Department of Biomedical Engineering, Johns Hopkins University, Baltimore, MD, USA; 11 Institute for NanoBioTechnology, Johns Hopkins University, Baltimore, MD, USA; 12 Department of Genetic Medicine, Johns Hopkins University, Baltimore, MD, USA; 13 Department of Electrical and Computer Engineering, Johns Hopkins University, Baltimore, MD, USA; 14 Data Science and AI Institute, Johns Hopkins University, Baltimore, MD, USA; 15 Department of Pathology, Johns Hopkins University, Baltimore, MD, USA; 16 Department of Cell Biology, Johns Hopkins University School of Medicine, Baltimore, MD, USA; 17 Department of Chemical and Biomolecular Engineering, Johns Hopkins University, Baltimore, MD, USA; 18 Memorial Sloan Kettering Cancer Center, New York, NY, USA; 19 Department of Informatics, Indiana University, Bloomington, IN, USA; 20 Department of Biomedical Engineering, Georgia Institute of Technology, Emory University, Atlanta, GA, USA; 21 Centre de Recherches en Cancerologie de Toulouse, Toulouse, France; 22 Department of Medicine, Washington University Saint Louis, St. Louis, MO, USA; 23 Giovannis Institute for Translational Cell Biology, Johns Hopkins University School of Medicine, Baltimore, MD, USA; 24 Department of Materials Science and Engineering, Johns Hopkins University, Baltimore, MD, USA; 25 Mays Cancer Center, University of Texas Health, San Antonio, TX, USA; 26 MD Anderson Cancer Center, San Antonio, TX, USA; 27 Translational Tissue Engineering Center, Johns Hopkins University School of Medicine, Baltimore, MD, USA; 28 Knight Cancer Institute, Oregon Health & Science University, Portland, OR, USA; 29 Department of Cell, Developmental and Cancer Biology, Oregon Health & Science University, Portland, OR, USA; 30 Department of Neurology, Johns Hopkins University School of Medicine, Baltimore, MD, USA; 31 Kavli Neuroscience Discovery Institute, Johns Hopkins University, Baltimore, MD, USA; 32 Department of Applied Mathematics and Statistics, Johns Hopkins University, Baltimore, MD, USA; 33 Department of Medicine, University of Maryland School of Medicine, Baltimore, MD, USA; 34 These authors contributed equally; 35 Lead contact

## Abstract

Cells interact as dynamically evolving ecosystems. While recent single-cell and spatial multi-omics technologies quantify individual cell characteristics, predicting their evolution requires mathematical modeling. We propose a conceptual framework—a cell behavior hypothesis grammar—that uses natural language statements (cell rules) to create mathematical models. This enables systematic integration of biological knowledge and multi-omics data to generate *in silico* models, enabling virtual “thought experiments” that test and expand our understanding of multicellular systems and generate new testable hypotheses. This paper motivates and describes the grammar, offers a reference implementation, and demonstrates its use in developing both *de novo* mechanistic models and those informed by multi-omics data. We show its potential through examples in cancer and its broader applicability in simulating brain development. This approach bridges biological, clinical, and systems biology research for mathematical modeling at scale, allowing the community to predict emergent multicellular behavior.

## In brief

We developed a plain text modeling language—a cell behavior hypothesis grammar—to easily build virtual cell models and connect them to data, helping scientists to unlock the hidden dynamics of tissues. We provide examples showing how to use them in virtual experiments exploring how cancer responds to the cells in its environment and how the brain forms layers in development.

## Graphical Abstract

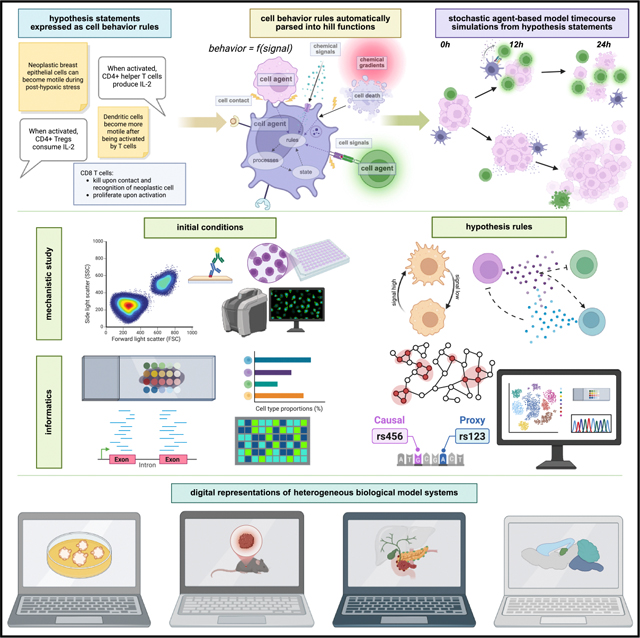

## INTRODUCTION

Generating temporally resolved multicellular predictions remains an open computational challenge.^1–3^ Bioinformatics techniques and machine learning can predict cellular trajectories and dynamic phenotypic changes in individual cell types from snapshots in single-cell assays,^4–6^ but they cannot account for more complex temporal changes throughout multicellular ecosystems. More advanced computational tools are needed to fill the gaps between measurement times and leverage biological knowledge and mechanism to forecast unseen emergent behaviors in multicellular systems *de novo*. Mechanistic mathematical modeling can extend static high-resolution data to multicellular dynamics. Agent-based modeling is a powerful mathematical modeling technique to predict emergent complex behaviors from populations of individual software agents that follow pre-defined rules based on their identity, state, and nearby conditions.^7^ Over a series of simulation time increments, each agent calculates its next action by evaluating its surroundings and internal state variables to calculate its next action. Agent-based models (ABMs) are well suited to studying the dynamics of multicellular biology, as each agent can encode a cell based on its state, type, and associated rules of behavior, including their actions upon or in response to nearby cells (i.e., cell-cell interactions).^8–11^ By encoding the rules of multicellular systems, ABMs empower *in silico* experimentation and modeling of cellular dynamics, even in the absence of temporal measurements.^9,12^ ABMs have been used as powerful *in silico* models to test hypotheses in human development and disease where comprehensive experimentation is not possible.^13–28^ By predicting the future state of cells and the impact of perturbations, ABMs provide a powerful toolset to generate digital twins and virtual clinical trials.^3,10,29–38^ Furthermore, the ability to run ABM simulations at scale across diverse biological conditions^38–40^ can refine biological understanding and predict future cellular behaviors in these complex systems. Altogether, these *in silico* models can prioritize bench experiments or clinical trials, addressing the costs and practical constraints of real-world experimentation.

While powerful, mathematical modeling lacks the language to *directly* connect to the vast accumulated knowledge of the biological community and to easily transform data into equations. As a result, widespread application of ABMs for modeling biological systems is currently limited both by the highly technical nature of most software implementations and the ability to integrate molecular data to ground simulations in the real world. The former issue gatekeeps ABMs away from those without significant computational experience, limiting widespread application and even posing a barrier for many potential users with extensive knowledge of the biological systems already encoded in ABMs. Even for advanced computational users, the custom coding required can limit reproducibility. Software implementing ABMs has been developed to overcome these limitations.^41–50^ Still, disease etiology and operating biological hypotheses are often hidden deep in source code, obscuring the assumptions about the system and the full set of hypotheses being simulated. These technical challenges to ABMs also limit the ability to embed molecular datasets, which are often too high-dimensional to manually encode into equations of agents and rules. A conceptual framing that can abstract cellular phenotypes and their interactions—combined with a simplifying coding infrastructure—is essential for the integration of molecular measurements to personalize model predictions. Facilitating *in silico* modeling requires both an intuitive language— to concisely express expert knowledge as plain text descriptions of the rules of cell interactions that “encode” a system—and also software to translate these plain text descriptions into mathematical expressions and executable models for immediate exploration of a digitized copy of the biological system.^47^

To enable human-interpretable construction of ABMs, we developed a cell behavior hypothesis grammar that bridges the divide between biology and mathematical modeling, by embracing well-defined human language hypotheses on cell behavior as a logical model that can be translated directly into the language of mathematical equations in an ABM. This one-for-one relationship between human language and mathematics allows us to systematically curate and integrate biological knowledge and high-throughput data to make biology *computable*.^51^ Briefly, our grammar defines the components of ABMs based on labeled cell types and behaviors. Rules can be both knowledge driven (e.g., expert statements drawing from literature and prior training) or data driven (directly measured from experimental data). This in turn enables virtual “thought experiments”^52^ that challenge and extend our understanding of multicellular systems and that generate new testable hypotheses. Thus, the grammar allows for broad application of ABMs in a reproducible, modular, and extensible manner. We demonstrate how this grammar’s reliance on annotated cellular states enables both encoding of expert-curated biological knowledge as well as high-throughput molecular profiling data, applying these techniques to sample models in cancer biology. These examples are progressively more complex and designed to span tumor cell growth, invasion, and response to immunotherapy. We then extend this grammar to a further example simulating brain development with models parameterized from spatial transcriptomics (ST) data in the Allen Brain Atlas,^53^ showing the broad applicability of our hypothesis grammar to biological systems beyond cancer. The cases embedding multi-omics and spatial molecular data enabled by this grammar demonstrate how to ground simulations in data to form more accurate digital models of multicellular dynamics.

## RESULTS

### A grammar encoding cell behavioral responses to extracellular signals

**Figure 1.**
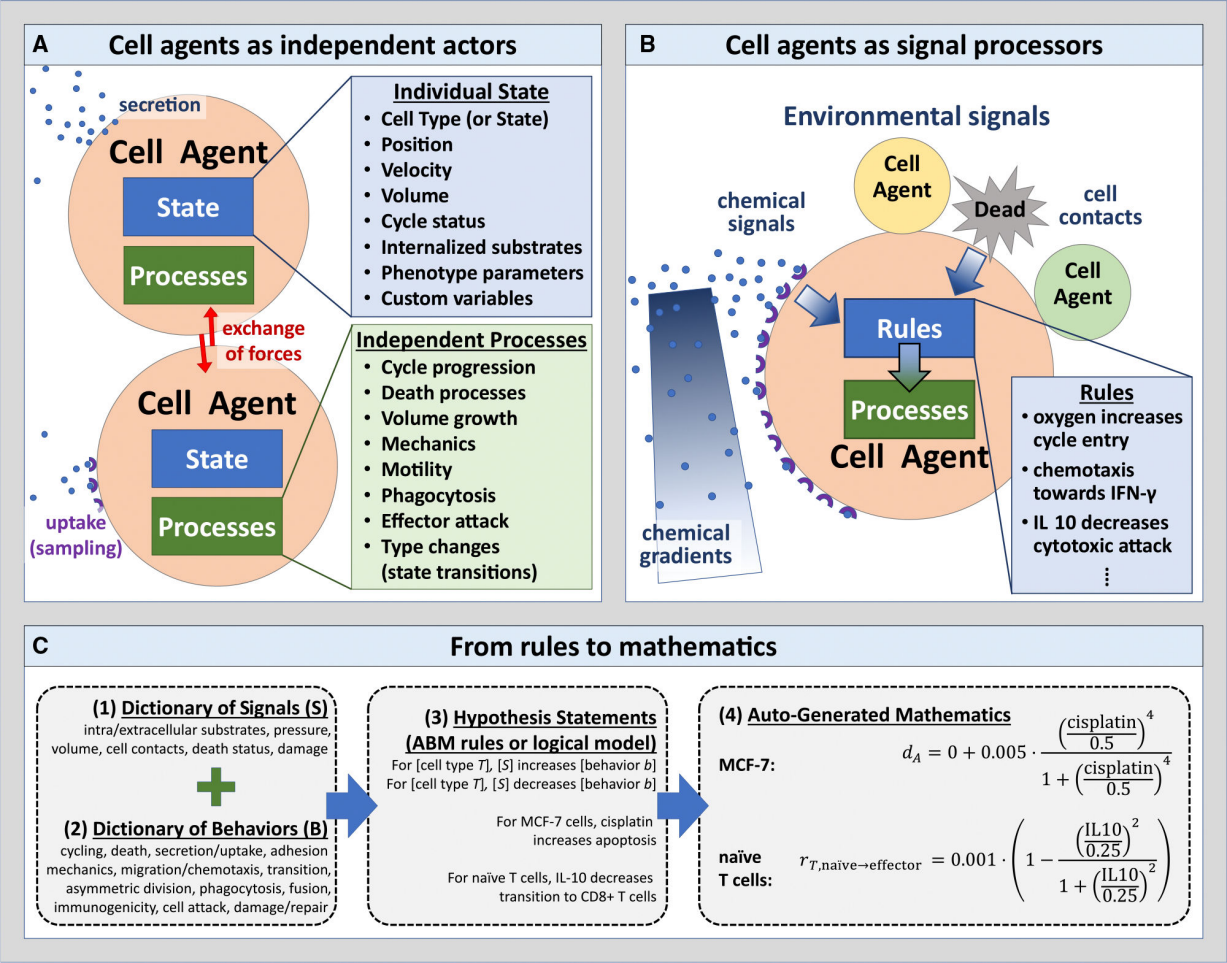
Using agent-based models to digitize cell knowledge (A) Agent-based models (ABMs) simulate cells as individual objects with separate states and processes. (B) Cell agents use rules that process biophysical signals in their microenvironment—including other cells—to drive changes in their behaviors. These rules are based on our biological hypotheses. (C) The cell behavior grammar combines signals and behaviors from well-defined dictionaries (1 and 2) to create interpretable hypothesis statements (3), which can be automatically transformed into mathematical models (4) for use in computer models.

In this paper, we implement our new hypothesis grammar for ABMs in the well-calibrated, robust agent-based modeling ecosystem of PhysiCell.^47^ ABM frameworks^8^ like PhysiCell^47^ model individual cells as software agents with independent states (e.g., position, cycle status) and processes (e.g., motility, secretion); see Figure 1A. Each cell agent responds to stimuli (signals) in their microenvironment, which effect changes in their behaviors (Figure 1B). Previous implementations of PhysiCell were limited to pre-defined models and interactions or required users to have expert knowledge across the diverse domains of biology, mathematics, and computer science to hand-code models with customized cellular agents, stimuli, and interactions. Here, we simplify this encoding by expanding the PhysiCell software to encode the agents and stimuli as human-readable sentences that are then parsed into ABMs. Briefly, this abstraction is enabled by writing cell hypotheses relating cell behavioral responses to signals in a grammar that can be translated into mathematics and executable code, as summarized below (e.g., typical rule in Figure 1C) and in detail in STAR Methods and Methods S1. In this hypothesis grammar, cellular behaviors and stimuli are expressed as nouns and their regulatory relationships as verbs, and parameters quantify these relationships. Hypotheses can be drawn from a variety of sources, including domain expertise, mining of prior literature, and analysis of transcriptomic and other data. Due to our uniform knowledge representation, all these rules can be compatibly integrated (Figures S1, S2, and S3) in mathematical models. Moreover, the use of plain language for cellular phenotypes also facilitates the direct mapping of ABM variables to the cellular labels inferred in analyses of single-cell and spatial multi-omics datasets.

In addition to the stimuli and cell types in the grammar and optional initial conditions provided from high-throughput molecular datasets, cellular behaviors simulated from ABMs also depend on both the parameters for the equations in these rules and the initial conditions of cellular phenotypes. While we implement the grammar using the PhysiCell agent-based modeling framework^47^ as a reference implementation, it can be translated to other agent-based modeling systems.^41,43–46,48–50^ Still, an advantage of building this hypothesis grammar on top of PhysiCell is that it enables us to use a broad set of biochemical and biophysical parameters, which has been previously quantified and experimentally validated in the extensive literature and community-based development using this modeling framework.^47^

The hypothesis grammar parses numeric variables for model parameters. Ideally, these parameters would be inferred from the literature or quantified from experimental data of the biological systems they seek to model. Parameter selection is a critical aspect of ABMs, as it can significantly affect simulation outcomes. While many parameters can be estimated experimentally or from the literature, the cellular and molecular heterogeneity of biological systems can differ between individuals and contexts. Throughout the development of PhysiCell, we have refined parameter selection processes in several ways (e.g., Bayesian approaches,^54^ large-scale parameter space sweeps,^38–40^ and community-developed tools^55^), laying the foundation for our hypothesis-based grammar and community-based outreach. Performing parameter sensitivity analysis remains a fundamental step in evaluating model performance. Prior to the grammar, implementing parameter sensitivity analyses generally required custom code to alter each model variable. We extend our software to include a graphical tool^56^ for exploring and tuning parameters of rules governing signals and behaviors to simplify *in silico* parameter exploration. Additionally, we created software for analysis across the entire input space of models (parameters, initial conditions, and hypotheses; see Methods S1). This additional software module^57^ is focused on *in silico* perturbations of parameters to test their sensitivity on model behavior.

### Order-of-magnitude parameter estimates robustly predict qualitative behaviors of oxygen-dependent tumor cell proliferation, with greatest sensitivity to cell motility

**Figure 2.**
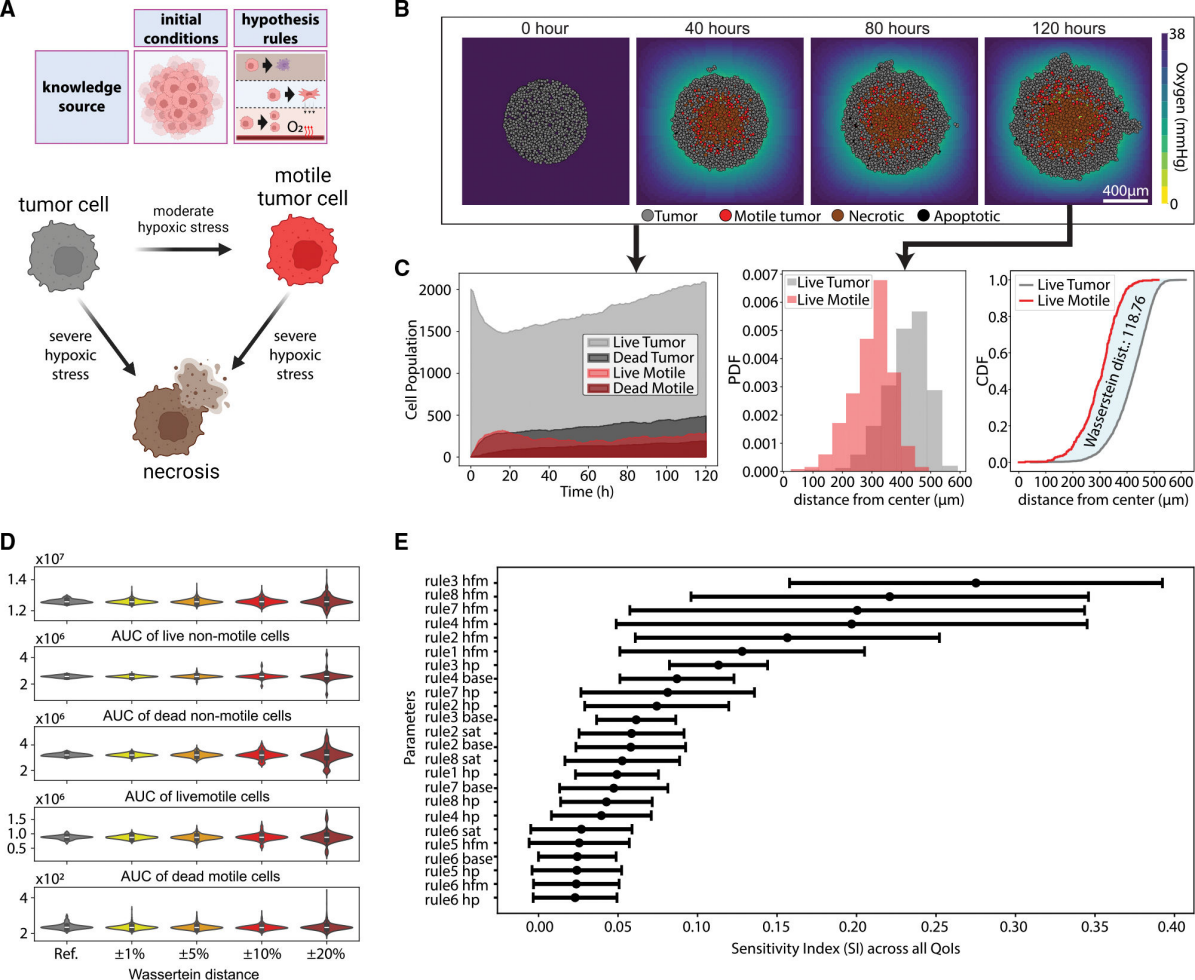
The hypoxia model captures the post-hypoxic dynamics (A) Schematic of the tumor hypoxia model. Initially, the tumor is homogeneous without immune infiltration. (B) Simulation of tumor hypoxia dynamics over 5 days, using reference parameters. (Bar: 400 μm.) (C) Metrics extracted from the simulation, including area under the curve (AUC) for cell populations over 5 days, the radial distribution of live non-motile and live motile tumor cells at endpoint, and the Wasserstein distance of these distributions. (D) Variation of quantities of interest (QoIs) under multiplicative perturbations in the 24D parameter space. (E) Mean and standard deviation of the sensitivity index for each parameter across all QoIs. Model rules are enumerated in the order of their insertion. Each rule includes parameters representing the base behavior (base), saturation level (sat), half-max signal value (hfm), and hill power (hp). Parameters such as *rule3_hfm* and *rule7_hfm* denote the oxygen half-max values that trigger necrosis in non-motile and motile cells, respectively. Similarly, *rule8_hfm* and *rule4_hfm* represent the oxygen half-max values for phenotype transitions between motile and non-motile cells. A full description of all parameters can be found in Methods S1.

In cancer, cell proliferation becomes unchecked, exhausting oxygen and nutrients in non-vascularized tumors. Modeling resource consumption provides a foundation for mathematical modeling of tumors, and this serves as the base example of tumor cell behavior in the absence of an immune response, from which other modeling examples are built.^47,54,58–64^ Following prior work developing a custom mathematical model of this system,^54^ we now adapt our hypothesis grammar to model hypoxia-induced migration, where low oxygen conditions can “reprogram” tumor cells to a transient, post-hypoxic phenotype of increased chemotactic migration and where subsequent prolonged exposure to non-hypoxic conditions can “revert” those cells back to a less motile phenotype (Figure 2A). The language encodes these cell behaviors by using the language “oxygen decreases necrosis” and “oxygen decreases transformation to motile tumor cells.”

We used these rules to simulate 5 days of growth of a 2D tumor in an environment of 38 mmHg oxygenation (physioxia^65^), starting from 2,000 viable cells seeded randomly in a virtual disk with a 400-*μ*m radius (Figure 2B). The ABM generated from these rules simulates a virtual tumor with an oxygen-poor necrotic core, while hypoxic cells disseminate throughout the virtual tumor with increasing frequency near the peri-necrotic boundary. Here, we observe an *in silico* model of a transient post-hypoxic phenotype of increased chemotactic migration, where cells eventually return to their baseline phenotype upon reoxygenation. Consistent with prior modeling predictions and experimental validation,^54,66^ these motile cells form invasive “plumes” in non-hypoxic tumor regions, but they can fail to exit the tumor and invade the surrounding tissue when their hypoxic adaptations do not persist in higher oxygen conditions (Figure 2B).

In this model, we selected parameters from our prior calibration of this model from literature-derived parameters and experimental validation to simulate the dynamics of an MDA-MB-231-derived orthotopic murine breast tumor model.^54,66^ We investigated the sensitivity of the model results to the parameter values, by computing the impact of varying parameter variations by 1% to 20% on key quantities of interest (QoIs). We assessed population growth by analyzing the area under curve (AUC) of non-motile and motile tumor cell populations and evaluated the differences in the radial distribution between live motile and non-motile cells (Figure 2C). The median values of the QoIs remained relatively stable across different perturbation levels in parameter space, indicating that the overall model behavior was not strongly influenced by small changes in these parameters. The variability of these outputs increased with higher perturbation levels, suggesting that while average behavior might be robust, individual simulations could exhibit greater variation (Figure 2D). Although individual simulation replicates showed variability consistent with the stochastic nature of tumor growth, key qualitative behaviors—such as the emergence of necrotic cores and the spatial gradients of cell proliferation and migration—were consistently observed across multiple replicates (Figure S4; Methods S1). This finding aligns with our recent large-scale investigation of highly stochastic tumor-immune ABMs,^38^ which found that the outcome of individual simulation replicates can vary widely, while large sets of replicates can remain concordant.

Perturbations to the base values of the rules did not lead to significant changes in the QoIs. Half-max values—particularly those associated with necrosis onset and transitions between motile and non-motile phenotypes—significantly influenced both the magnitude and timing of tumor growth and the spatial distributions of cell populations (Figure 2E). These findings underscore the importance of careful parameter estimation and rigorous sensitivity analysis in the application of ABMs, emphasizing the need for experimental quantification of the contribution of individual parameters to emergent behaviors in tumors.^38^

### Rules simulating fibroblast and neoplastic cells in pancreatic cancer demonstrate that fibroblasts promote invasion and physically block progression

**Figure 3.**
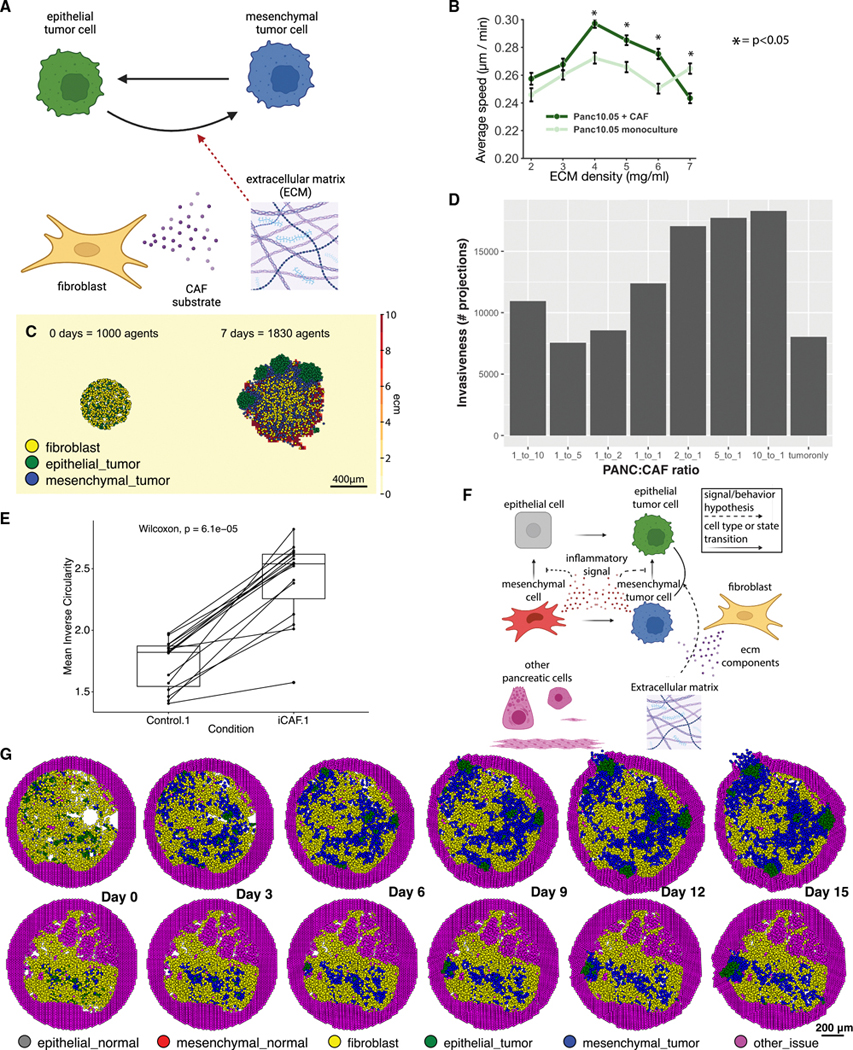
CAFs support the epithelial-to-mesenchymal transition in simulated pancreatic epithelial cells, which promotes invasive growth and the establishment of new epithelial-like foci (A) Schematic of the CAF-epithelial model. (B) Monoculture and co-culture PANC migration speed vs. ECM density. (C) Simulation of CAF-epithelial dynamics initialized at a CAF:epithelial ratio of 1:1 over 7 days. (D) Mean invasiveness for each simulated admixture (PDAC:CAF). (E) Patient-derived pancreatic organoids (PDOs) are significantly more invasive when cultured in inflammatory CAF (iCAF)-conditioned media as compared with control. (F) Schematic of the extended CAF-epithelial model for integration with Visium data. (G) Simulations of samples PDAC01 (bottom row) and PDAC02 (top row) Visium tissue over 15 days. (Bar: 200 μm.)

In contrast to the hypoxia-derived tumor progression in our previous model, pancreatic ductal adenocarcinoma (PDAC) tumors are characterized by a dense stroma consisting of cancer associated fibroblasts (CAFs) that have a dual role in promoting and hindering tumor growth. We sought to adapt the rules framework to model CAF and tumor cell interactions informed by ST and single-cell RNA sequencing (scRNA-seq) datasets of PDAC. Our previous studies of cell-cell interactions in scRNA-seq analysis and organoid co-cultures found that fibroblasts induce epithelial-to-mesenchymal transition (EMT) in neoplastic cells through communication via the extracellular matrix (ECM)- sensing integrin receptor ITGB1.^67^ This neoplastic cell phenotype was mutually exclusive with proliferative signaling in epithelial cells. Therefore, we encoded two neoplastic cell subtypes in our model: an epithelial cell type that proliferates and a mesenchymal cell type that undergoes EMT but does not proliferate (Figure 3A).

The tumor cell states and parameters in our oxygen-dependent tumor growth model provided a foundation for modeling the neoplastic cell states in PDAC, particularly the proliferative phenotype associated with the more epithelial-like neoplastic cells. The more complex biophysical impact of fibroblast and ECM interactions and the precise cellular behavior of EMT in this mesenchymal subtype of cells were not sufficiently well described to encode model rules or parameters. To address this limitation, we performed single-cell tracking experiments^68^ to assess the impact of both the ECM and fibroblasts on human pancreatic tumor cells. Co-culturing PDAC cells with CAFs increased motility except in the highest ECM concentrations, as compared with monoculture, leading us to hypothesize that increased cell motility is a dominant feature of fibroblast-mediated signaling on neoplastic cells (Figures 3B, S5, S6, S7, and S8; Methods S1). These data also showed that ECM density has a complex effect on neoplastic cell motility: as collagen-I density was increased in monoculture, tumor cell motility first increased and then decreased, which was consistent with prior published observations from other cancer types.^68,69^

Based on our gene expression analysis and these experimental data, we hypothesized that fibroblasts secrete factors that both alter ECM density and promote neoplastic cell phenotype changes to increase motility in the co-culture condition. We encoded these 2-fold effects in the ABM (where signaling from fibroblasts promotes EMT in neoplastic cells) by adding secretion of a simulated factor from fibroblasts, which promotes a phenotypic shift from epithelial-like to mesenchymal-like. This transformation rate depended on the local ECM density, with the rate increasing from 0 to 0.01 min^−1^ as the ECM density increases. We described the neoplastic cell motility response to its local ECM density using two Hill functions, which combined to produce the biphasic motility behavior observed in our experiments (Figure S9; Methods S1). Based on the notable sensitivity of our ABM to motile neoplastic cells, we sought to further use our experimental data to parameterize motility rates in this ABM to more accurately reflect PDAC biology. To isolate the CAF signaling effect on mesenchymal-like neoplastic cell motility from the effect on EMT induction, we used the *in vitro* PDAC monoculture cell motility data to parameterize the *in silico* migration rate of mesenchymal cells as a function of ECM density (Figures S3 and S9; Methods S1). In this way, the fibroblasts in this model could shift the microenvironment in favor of tumor progression, consistent with the hypothesis generated from our transcriptional signatures and the *in vitro* cell motility data. Finally, to encode the switch between this mesenchymal phenotype and the alternative epithelial-like subtype, we simulated a simple (generalized) pro-inflammatory factor which was pro-tumorigenic and induced proliferative signaling in the epithelial-type neoplastic cells.

To first simulate the impact of CAF density on cancer progression, we generated a series of ABMs of virtual co-culture experiments at varying cell densities. We initialized the model by seeding a total of 1,000 cells at various PANC:CAF ratios (using the rules and parameters derived above to simulate Panc 10.05 and HT-231 cell behavior) for 7 simulated days (Figure 3C; Videos S1–S8). In the simulations, fibroblasts promoted tumor cell invasion. We sought to quantify the impact of fibroblast density on this invasion by counting the number of invasive projections of simulated tumor cells away from the central tumor mass over time (STAR Methods). By 24 h, the simulated co-cultures all had a significant increase in invasion relative to simulated monoculture, with the highest levels of invasion observed in the 10:1 and 5:1 PANC:CAF ratio simulations (Figure 3D). This enhanced invasion in the 10:1 and 5:1 PANC:CAF ratio simulations was observed across all time points, while the 2:1 PANC:CAF simulation reached a similar level of invasion at later time points. On the other hand, the 1:5 and 1:10 PANC:CAF ratio simulations reach similar levels of invasion to monoculture at later time points.

To validate the observed impact of CAF-related signaling on neoplastic cell invasion, we also performed *in vitro* cultures of a panel of patient-derived PDAC organoids in CAF-conditioned media and measured the change in invasion (Figure 3E). While PDAC organoids invaded when embedded in collagen alone,^70^ we observed significantly increased invasion in the CAF-conditioned media in these experiments, consistent with the hypothesis that secreted factors from CAFs are sufficient to induce invasion. One limitation of our model was its abstraction into a single CAF subtype. To test whether this was sufficient to capture the invasive process, the invasion assays in PDAC organoids were performed in conditioned media from inflammatory CAFs (iCAFs) and myofibroblastic CAFs (myCAFs).^71^ We observed similar levels of invasion with both CAF subtypes, supporting the abstraction of a single subtype of CAFs for our initial models (Figure S10).

We next sought to identify the potential of adapting our ABM to forecast cellular states based on initial conditions from human tissue. For this mathematical model, we initialized cell positions in a virtual tissue based on Visium ST data from two human PDAC lesions selected for their high fibroblast density^72^ (Figures 3F and 3G). Our bioinformatics methods for three-way integration between H&E imaging data, ST, and transcriptional signatures of cellular phenotypes^72^ (see STAR Methods) were used to categorize and position the epithelial-like and mesenchymal-like neoplastic cell phenotypes, fibroblasts, and ECM in our model. Other cell types identified in the Visium data were modeled as essentially inert, providing structure and scaffolding for the tumor cells and CAFs. Cellular phenotypes annotated in the two Visium datasets were input into PhysiCell and used to initialize two distinct ABMs. Each lesion’s development was forecasted for 15 days (Figure 3G; Videos S9 and S10). We observed a transitory state in which the neoplastic cells transitioned from mixed epithelial and mesenchymal states to become nearly uniformly mesenchymal due to interactions with fibroblasts. Subsequently, groups of epithelial neoplastic cells arose in both models and even invaded beyond the tumor boundary.

Initializing the ABM from ST data could also estimate regional changes to cellular phenotypes and the impact of tumor heterogeneity. In both simulations, an interface of mesenchymal neoplastic cells was maintained between the epithelial neoplastic cell and fibroblast cell masses. In PDAC02, rapidly dividing epithelial neoplastic cell clusters arose from lesions not surrounded by fibroblasts and invaded the bounding pancreatic cells. In contrast, the dense, uniform fibroblasts surrounding all of the lesions in PDAC01 slowed invasion. The reduced rate of invasion resulted in smaller invasive lesions at 15 days in the PDAC01 sample compared with PDAC02. Whereas all the lesions in PDAC02 invaded the bounding pancreatic cells, PDAC01 developed an epithelial neoplastic cell mass constrained from further motility by the surrounding CAFs and dense ECM they had constructed. These computational predictions showed the hypothesized neoplastic-fibroblast interactions inducing the transition between classical (epithelial-like) and basal (mesenchymal-like) pancreatic transcriptional subtypes, as observed in primary human pancreatic tumor progression, and the return to a more epithelial-like classical subtype at metastatic sites.^73–75^ Moreover, the spatially resolved simulations from tissue also demonstrated how CAFs, despite their tumor-promoting behavior, can also serve as a physical barrier to prevent neoplastic cell invasion. These simulations of PDAC showed how we can parameterize ABMs from transcriptional analysis and cellular-level biophysical measurements, yielding simulations that inform experimentally testable hypotheses.

### Development of immune resistance in a diverse TME of T cells and macrophages

**Figure 4.**
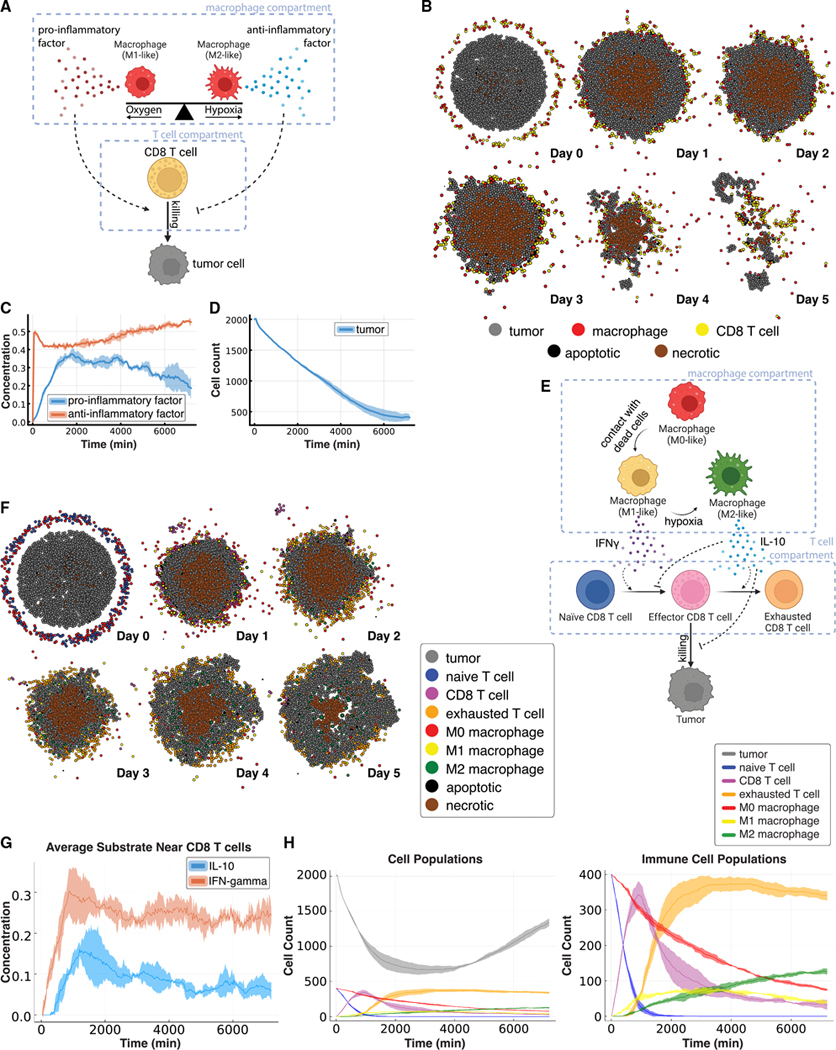
A simulated tumor evades cytotoxic killing by manipulating its immune microenvironment (A) Schematic of the tumor-immune interaction model. (B) Simulation of tumor-immune interaction model over 5 days showing tumor killing. (C) Time series of average substrate levels surrounding CD8^+^ T cells. Shaded regions indicate one standard deviation. (D) Time series of tumor cell count. Shaded regions indicate one standard deviation. (E) Schematic of the extended tumor-immune model, with three possible macrophage states and three CD8^+^ T cell states. (F) Simulation of extended tumor-immune model over 5 days, showing tumor survival after exhausting T cells and pushing more macrophages to the M2-like state. (G) Time series of average substrate levels surrounding CD8^+^ T cells. Shaded regions indicate one standard deviation. (H) Growth curves for all populations; the right plot zooms in to show just the immune dynamics, the exhaustion of T cells, and the switch toward M2-like macrophages. Shaded regions indicate one standard deviation.

To introduce virtual immune cells, we extended the ABM by including CD8^+^ T cell agents capable of contact-mediated cytotoxic killing, as well as phenotypically diverse macrophage populations. We developed the rules so that CD8^+^ T cells were also stimulated by pro- and anti-inflammatory factors that modulate the probability that killing will occur after a given cell contact. Simulated macrophages switched between promoting and suppressing tumor killing (secreting a pro- or anti-inflammatory factor, respectively) depending on the oxygenation in their immediate surroundings and as described in the literature.^76–78^ Macrophages were also responsible for phagocytosing dead cells and could increase secretion of pro-inflammatory factors, attracting CD8^+^ T cells that homed to the tumor by following this chemokine. CD8^+^ T cells could attack and damage malignant epithelial cells, and accumulated damage could cause tumor cell death. In tissue culture, macrophages can be polarized into cell states commonly referred to as M1 and M2. These phenotypes are plastic, and in our ABM, macrophages could transition between M1-like and M2-like depending on the signals in its environment, consistent with the literature^76–78^ (Figure 4A).

We used these rules to simulate 5 days of growth of a 2D tumor in tissue culture in a virtual environment of 38 mmHg oxygenation (physioxic conditions^65^), starting with 2,000 viable tumor cells seeded randomly, surrounded by a ring of immune cells seeded with 100 of each non-tumor cell type (Figure 4B). Through these simulations, we observed that CD8^+^ T cells clustered together and migrated throughout the tumor along with macrophages to accomplish tumor clearance, with a corresponding dominance of pro-inflammatory factor as the simulation proceeded (Figures 4C and 4D). This model showed how the innate and adaptive immune systems cooperated in the task of tumor sensing and clearance and demonstrated a simplified, plastic M1-like to M2-like axis of macrophage behavior in tissue culture, marked by rapid and reversible changes along this axis. We anticipated that the parameterization of immune cell models would also impact simulations results. By default, we chose parameter values based on existing literature^79–82^ to ensure biological relevance. To further investigate parameter sensitivity, we applied our new parameter sensitivity toolbox enabled by our rules to perform a local sensitivity analysis around the selected parameters using multiplicative perturbations of 1%, 5%, 10%, and 20%. We observed that while these perturbations introduced variation in the QoIs, they preserved the central tendency of the model outputs. We found that the half-max oxygen value for macrophage polarization had the greatest impact on the selected QoIs (Figures S11 and S12). This uncertainty quantification was consistent with the significant impact of macrophages on immune response and immunosuppressive progression during carcinogenesis.

To further model immune response initiation and macrophage-mediated resistance in tissue culture, we extended the immune cell subtypes in our model to represent M0-like, M1-like, and M2-like macrophages and naive, activated, and exhausted CD8^+^ T cell subtypes (Figure 4E). While *in vivo* macrophage populations do not polarize into discrete states in this way, we applied these categories as ways of broadly characterizing macrophages as either pro- or anti-inflammatory. Interleukin-10 (IL-10) and interferon (IFN)-γ modulated the activation of naive T cells with (pro-inflammatory) IFN-γ promoting and (anti-inflammatory) IL-10 inhibiting this activation. In the activated CD8^+^ T cell compartment, IFN-γ and IL-10 promoted proliferation and exhaustion, respectively. We initialized the tumor as before with 2,000 tumor cells inside a disc. The immune compartment was initialized with 400 M0-like macrophages and 400 naive CD8^+^ T cells in a ring around the disc of tumor cells (Figure 4F). ABM simulations demonstrated dynamics where pro- and anti-inflammatory factors occupied the neighborhood of the CD8^+^ T cells in roughly equal proportions throughout the simulation (Figure 4G) that initially caused the tumor population to shrink by more than 50% before recovering to nearly its original volume by day 5 (Figure 4H). This was facilitated by an immune compartment that initially had a rapid activation of naive T cells and a slower exhaustion of these newly activated CD8^+^ T cells (Figure 4H). The macrophage compartment accelerated both shifts with M1-like macrophages secreting IFN-γ to help activate T cells and later M2-like macrophages secreting IL-10 to induce CD8^+^ T cell exhaustion. The level of hypoxia modulated the balance between the M1-like and M2-like macrophage populations through its regulation of the transition from the pro-inflammatory M1-like state to the anti-inflammatory M2-like state. By the end of the simulation, the CD8^+^ T cell compartment was entirely exhausted, and the macrophage compartment inside the tumor boundaries was entirely M2-like (Figures 4F, later time points and 4H), permitting significant tumor regrowth. These immune dynamics were responsible for the initial CD8^+^ T cell-induced regression of the tumor and subsequent resistance to immune attack, as commonly observed in late-stage, immunosuppressive tumors.

### Modeling macrophage-induced invasion generates the experimentally testable hypothesis that EGFR signaling promotes neoplastic cell motility in breast cancer

**Figure 5.**
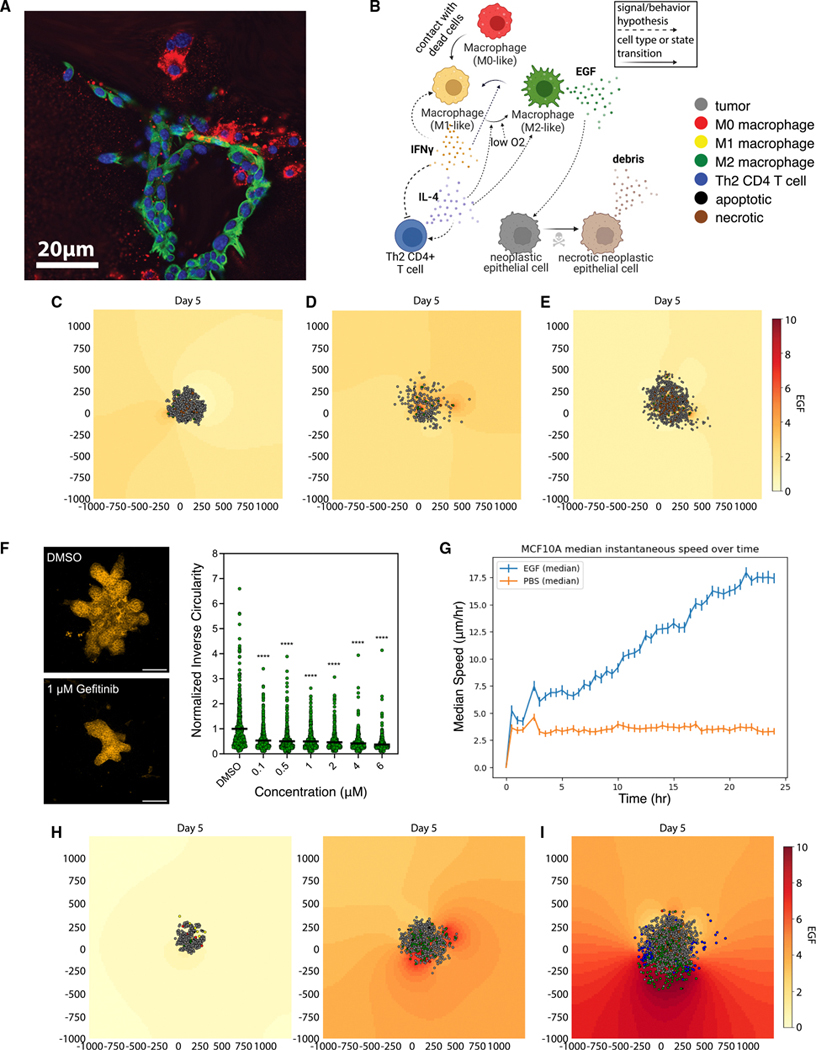
Tumor-associated macrophages in M2-like polarization state assist simulated invasive breast cancer spheroids through EGF signaling (A) Example fluorescence microscopy image showing macrophages (red) proximal to an invading PyMT organoid (green), with DAPI shown in blue indicating cell nuclei, as an additional replicate image of the experiments described originally in DeNardo et al.^84^ (Bar: 20 μm.) (B) Schematic of the tumor-associated macrophage (TAM)-EGF model, including TAMs, CD4^+^ T cells, and neoplastic epithelial cells. (C–E) Endpoint snapshots of simulations in which EGF signaling causes neoplastic cells to increase proliferation (C), increase motility (D), and increase both proliferation and motility (E). (F) EGFR inhibitor inhibits MMTV-PyMT invasion into 3D collagen I in a dose-dependent manner. ****p < 0.0001 (Kruskal-Wallis followed by Dunn’s multiple comparisons test). (G) Stimulated MFC10a breast epithelial cells exhibit increased motility when exposed to EGF, with increasing median migration speed as more cells escape the tumor bulk for unrestricted migration. (H) Endpoint snapshots treated with IFN-γ (left panel) and IL-4 (right panel). (I) Endpoint snapshot with Th2-like CD4^+^ T cells.

Because CD4^+^ helper T cells are a major component of the tumor immune ecosystem that modulates the immune response on tumor progression,^83^ we sought to extend our ABM investigation to uncover mechanisms associated with immune-induced tumor progression by simulating the influence of CD4^+^ helper T cells on epithelial cell behaviors. Notably, DeNardo et al. previously demonstrated that signaling from Th2 CD4^+^ T cells to macrophages can induce pro-tumorigenic effects in the MMTV-PyMT murine breast cancer spheroid model.^84^ Briefly, the study demonstrated that Th2 CD4^+^ T cell signaling promotes changes in macrophage phenotype, making them more likely to produce EGF and therefore stimulate EGFR signaling in tumor cells driven by immunosuppressive macrophages, promoting invasive behavior via EGFR signaling (Figure 5A). We sought to determine if our ABM simulations can reproduce this emergent, seemingly counter-intuitive tumor-promoting behavior arising during immune response.

We first sought to simulate the series of experiments from DeNardo et al.^84^ that used a tumor spheroid co-culture model to evaluate the impact of macrophage phenotypes on invasion. Briefly, we simulated the M1-like macrophages as pro-inflammatory and M2-like macrophages as anti-inflammatory, and we encoded their role in phagocytosing dead cells and secreting pro- and anti-inflammatory factors. In this system, the M2-like macrophages also promoted pro-tumorigenic signaling in the neoplastic cells through the EGF-EGFR signaling pathway (Figure 5B). We used these rules to simulate 5 days of virtual spheroid growth in 38 mmHg oxygenation (physioxia^65^), starting from 200 viable tumor cells seeded randomly, surrounded by macrophages overlaid in co-culture where 10 of each immune cell type (macrophage, T cell) are seeded. As a first step in mirroring the macrophage promotion of tumor invasion, we simulated EGF secretion from M2-like macrophages activating pro-tumorigenic behavior of neoplastic cells through EGFR. Canonically, EGFR signaling is hypothesized to act primarily by promoting tumor progression by inducing neoplastic epithelial cell proliferation, which we term the “grow hypothesis.” We simulated this by specifying a Hill response rule with the signal of EGF modulating cell-cycle entry in malignant epithelial cells, using the hypothesis grammar (Figure 5C; Video S11). However, these simulations did not demonstrate the macrophage-induced invasive tumor structures from DeNardo et al.^84^ Our PDAC simulations demonstrated that enhanced cellular motility was critical to simulate invasion. We modeled EGFR signaling from macrophages as promoting motility in breast cancer cells, a hypothesis supported experimentally.^85,86^ We term this the “go hypothesis” (Figure 5D; Video S12). We simulated this increase in motility using a grammar rule specifying a Hill response function between EGF and malignant epithelial cell motility. The experimentally observed invasive phenotype was recapitulated in models in which EGF induced only changes to motility (Figure 5D) and models where EGF induced both motility and proliferation (Figure 5E; Video S13).

Our simulations led to the hypothesis that EGF-EGFR signaling in breast cancer cells promotes invasive outcomes primarily through modulating malignant epithelial cellular motility. To test these computational predictions experimentally, we treated organoids derived from the same MMTV-PyMT model with the EGFR inhibitor gefitinib. These experiments demonstrated that inhibition of EGF receptor signaling reduced the ability of neoplastic cells to form invasive protrusions (Figure 5F) and only reduced colony formation at extremely high doses of gefitinib treatment (Figure S13). In addition, MCF10A mammary epithelial cells exposed to EGF showed both increased proliferation and increased motility (Figures 5G and S14). Together, these experiments confirmed our computationally driven hypothesis that macrophage-induced invasion arises from EGFR signaling promoting neoplastic cell motility, consistent with the go hypothesis or go and grow hypotheses.

We next sought to integrate the effect of Th2-like CD4 helper T cells that secrete cytokines, which we^84^ and others^87^ have shown skew macrophages toward M2-like phenotypes and induce their proliferation. Mirroring the DeNardo et al. experiments, we performed *in silico* stimulation of the MMTV-PyMT-macrophage co-culture system first with the cytokine IFN-γ and then with IL-4. In our simulations, we observed that IFN-γ completely constrained tumor growth, whereas IL-4 promoted macrophage polarization toward an M2-like phenotype, inducing their proliferation and secretion of EGF into the local environment. The increased EGF levels then promoted tumor proliferation and motility, resulting in an expansion of tumor volume, compared with the IFN-γ condition (Figure 5H). Finally, we layered Th2-like CD4^+^ T cell agents into our model, which acted as cellular sources of IL-4 and promoted invasion similarly to the simulated high IL-4 dose, but with a more irregular shape resulting from a more irregular supply of IL-4 and subsequently EGF around the tumor boundary (Figure 5I). Taken together, these results support a dual role for EGF in promoting invasion. They also demonstrated how the rules-based modeling framework allows for *in silico* testing of cellular hypotheses of experimental phenomena and how it supports distinguishing alternative mechanistic hypotheses that can subsequently be tested experimentally to build toward a systems-level model of emergent, unanticipated cell behavior.

### Leveraging the hypothesis grammar for virtual clinical trials: Using human scRNA-seq data from PDAC to simulate immunotherapy combinations

**Figure 6.**
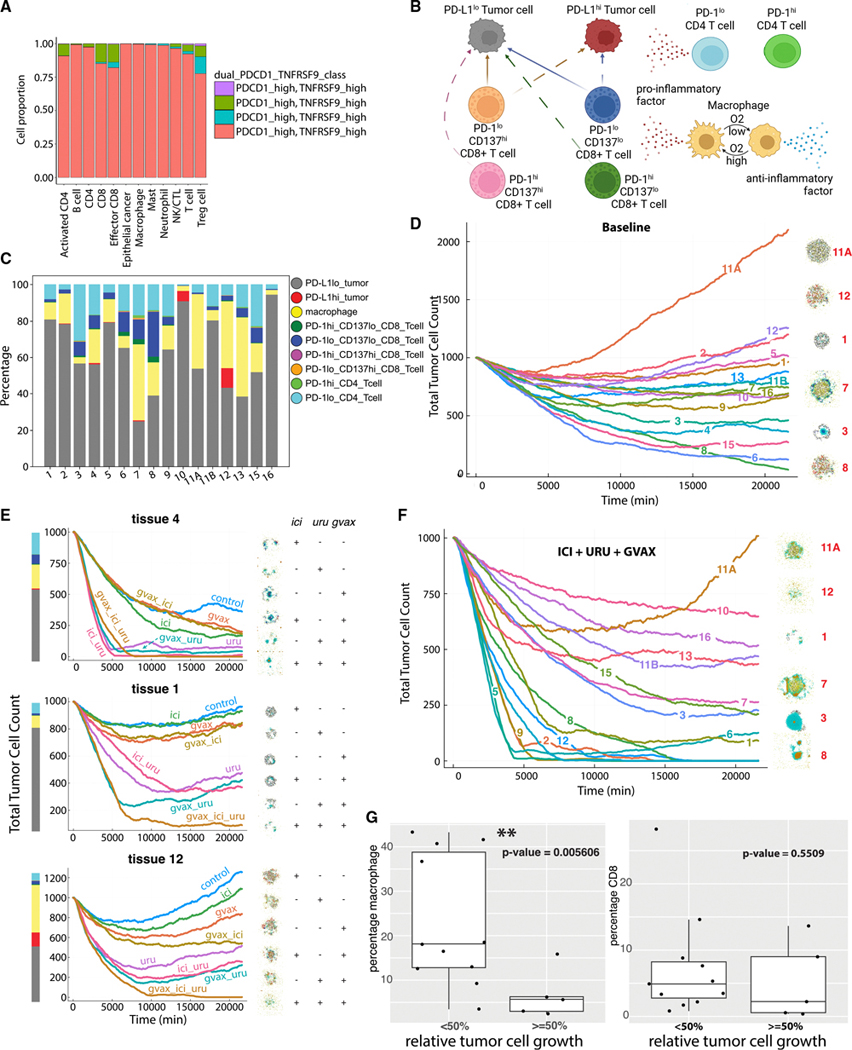
Combination immunotherapies simulated for a cohort of untreated pancreatic adenocarcinomas based on immune cell proportions estimated from scRNA-seq data (A) Expression of marker genes of interest within immune populations in immune-enriched scRNA-seq data in a cohort of PDAC tumors from Steele et al.^92^ (B) Schematic of the combination immunotherapy model. (C) Populations of interest identified for each tumor profiled in the reference scRNA-seq data. (D) Tumor growth curves per each tissue initial conditions without therapy, with images shown at the simulation endpoint for select samples. (E) Growth curves under each therapy condition for three example tissues, with endpoints shown. (F) Tumor growth curves for the triple-therapy treatment condition (ICI + URU + GVAX), images shown at simulation endpoint for select samples. (G) Macrophage and CD8 T cell population relative abundances in tissues, binned by whether the simulation with GVAX + ICI + URU reached the tumor clearance threshold (final/initial × 100% < 50%). Macrophage abundance was significantly higher in tissues whose simulations reached the tumor clearance threshold (*p* = 0.005606), while CD8^+^ T cell abundance did not show a significant difference (*p* = 0.5509).

Encoding the tumor microenvironment (TME) in our ABM can simulate the impact of immune cell composition and perturbations on tumor growth. We sought to adapt this framework to simulate the impact of different immune-targeted therapies in the PDAC microenvironment to develop a virtual simulation of a clinical trial. In particular, we were motivated by a recent neoadjuvant clinical trial^88,89^ that sought to enhance T cell-mediated cytotoxicity in PDAC by adding Urulemab (an anti-CD137 agonist therapy) to a combination of GVAX (an irradiated, granulocyte-macrophage colony-stimulating factor [GM-CSF]- secreting, allogeneic PDAC vaccine)^90^ and Nivolumab (an anti-PD-1 immune checkpoint inhibitor). To generate the model rules reflecting this clinical trial, we used observations from high-throughput transcriptomic data (Figure 6A), our preceding immune cell models, and additional literature-derived hypotheses about the phenotypes of immune cells (Figure 6B). Owing to the nature of these therapies, we implemented rules to model T cell, tumor cell, and macrophage behavior. We used template rules for tumor cells and macrophages, modeling proliferative tumor cells as in previous ABM examples and macrophages as plastic between pro- and anti-inflammatory states but biased toward anti-inflammatory factor secretion. We then developed rules for T cells. To reflect the therapeutics in the trial design, we modeled CD4^+^ and CD8^+^ T cells subtyped based on their PD-1 and CD137 expression. Multi-omics data from the arms of this trial, comparing GVAX monotherapy with the combination with Nivolumab, demonstrated that PD-1 inhibition activates chemokine signaling in CD4^+^ T cells, thereby signaling to CD8^+^ T cells to promote changes in lymphocyte chemotaxis.^91^ Based on these data, we created distinct model rules for T cells based on their PD-1 expression, specifically that CD4^+^ T cells secrete chemokines that attract CD8^+^ T cells (here designated pro-inflammatory factor). We performed further ligand-receptor analysis of cell-cell signaling associated with CD137^hi^ CD8^+^ T cells using scRNA-seq data from untreated PDAC tumors and found that CD137^hi^ CD8^+^ T cells have higher signaling through IFN expression than their CD137^lo^ counterparts.^91^ We modeled this subtype-specific IFN expression by specifying that CD137^+^ cell agents secrete an inflammatory factor substrate into the surrounding environment. Stimulation through the CD137 transmembrane receptor is also known to make CD8^+^ T cells more effective at killing, which we simulated by modeling CD137^hi^ CD8^+^ T cells as having a higher killing rate than their CD137^lo^ counterparts and having the ability to kill tumor cells independent of their PD-L1 status.

We then simulated the effect of therapy on different microenvironments—a key step to creating virtual clinical trials that explore therapeutic response across a variety of tissue conditions. Using the rules to generate virtual clinical trials requires having both a mechanism of action of the therapies in our rules and the immune cell compositions for a cohort of virtual PDAC tumors. We simulated the effect of therapy by modifying the proportion of each T cell subtype consistent with the canonical mechanism of action of that therapy. Specifically, we simulated GVAX treatment by a doubling of all T cell populations in the TME, Nivolumab therapy by turning PD-1^hi^ agents into their PD-1^lo^ counterparts and PD-L1^hi^ agents into PD-1^lo^, and Urelumab by turning all CD137^lo^ agents into CD137^hi^ agents. We kept these cellular interaction rules consistent across all individual simulated patients; the efficacy of a simulated treatment thus depended upon the proportion of immune cell populations in each virtual patient. To simulate clinically relevant immune compartments, we generated an *in silico* cohort of tumors based on the immune cell-type distributions in untreated scRNA-seq data from Steele et al.^92^ To mirror the cellular phenotypes in our model rules, we re-annotated the scRNA-seq data from Steele et al.^92^ as described by Guinn et al.^67^ and Li et al.,^91^ sorting T cells into hi/lo categories based on *TNFRSF9* (CD137) and *PDCD1* (PD-1) gene expression^91^ (Figure 6C). Tumor cells were classified as PD-L1^hi^ and PD-L1^lo^, based on scRNA-seq measurements of *CD274* (PD-L1). In our simulations, we initialized each microenvironment with 1,000 tumor cells, with the proportion of PD-L1^lo^ to PD-L1^hi^ expression from the scRNA-seq data.

We then combined our cell rules and TME composition from the scRNA-seq cohort to demonstrate how our modeling framework can be applied to test hypotheses about the impact of the TME composition on response to different therapies. As a virtual control to test the impact of therapeutics, we also ran these simulations for the baseline TMEs without therapy. The simulations from most patient microenvironments predicted neoplastic cell population growth without treatment after 7 simulated days. We then applied the simulations for each candidate therapy alone or in combination to each microenvironment; these simulations demonstrated inter-patient heterogeneity of treatment effects (Figures 6D–6F, S15 and S16). The baseline tumor growth profiles vary from near elimination (tissue 8) to uncontrolled tumor growth (tissue 11A), with others falling in between (Figure 6D). At endpoint, several simulated tissues displayed local aggregations of T cells, which were enriched in our simulated treatments, similar to the lymphoid aggregates found after immunotherapy treatment in biospecimens from human clinical trials^88,93^ (Figures 6D–6F, S17 and S18).

An advantage of our modeling framework is that it can simulate the temporal dynamics of cellular phenotypes observed in the TME. These temporal simulations provide the opportunity to evaluate the impact of the TME composition on simulated reduction in tumor volume (Figures S19–S26). In our simulations, we observed a statistically significant higher abundance of macrophages between responders and non-responders to triple combination (Figure 6G). While the triple combination converted the most T cells to the best killing state, we found that single or double combinations outperformed the triple combination for several tissue ABMs in the cohort. These simulations led to a new biological hypothesis that macrophage clearing of tumor cells is essential for lymphocyte trafficking and tumor cell killing in PDAC. We note that this hypothesis generated from our mathematical simulations is consistent with clinical observations of increased TREM2+ macrophage signaling to tumor cells in the triple combination.^89^

### Encoding asymmetric cell division in rules enables extension of the hypothesis grammar beyond cancer to simulate formulation of layers in brain development

**Figure 7.**
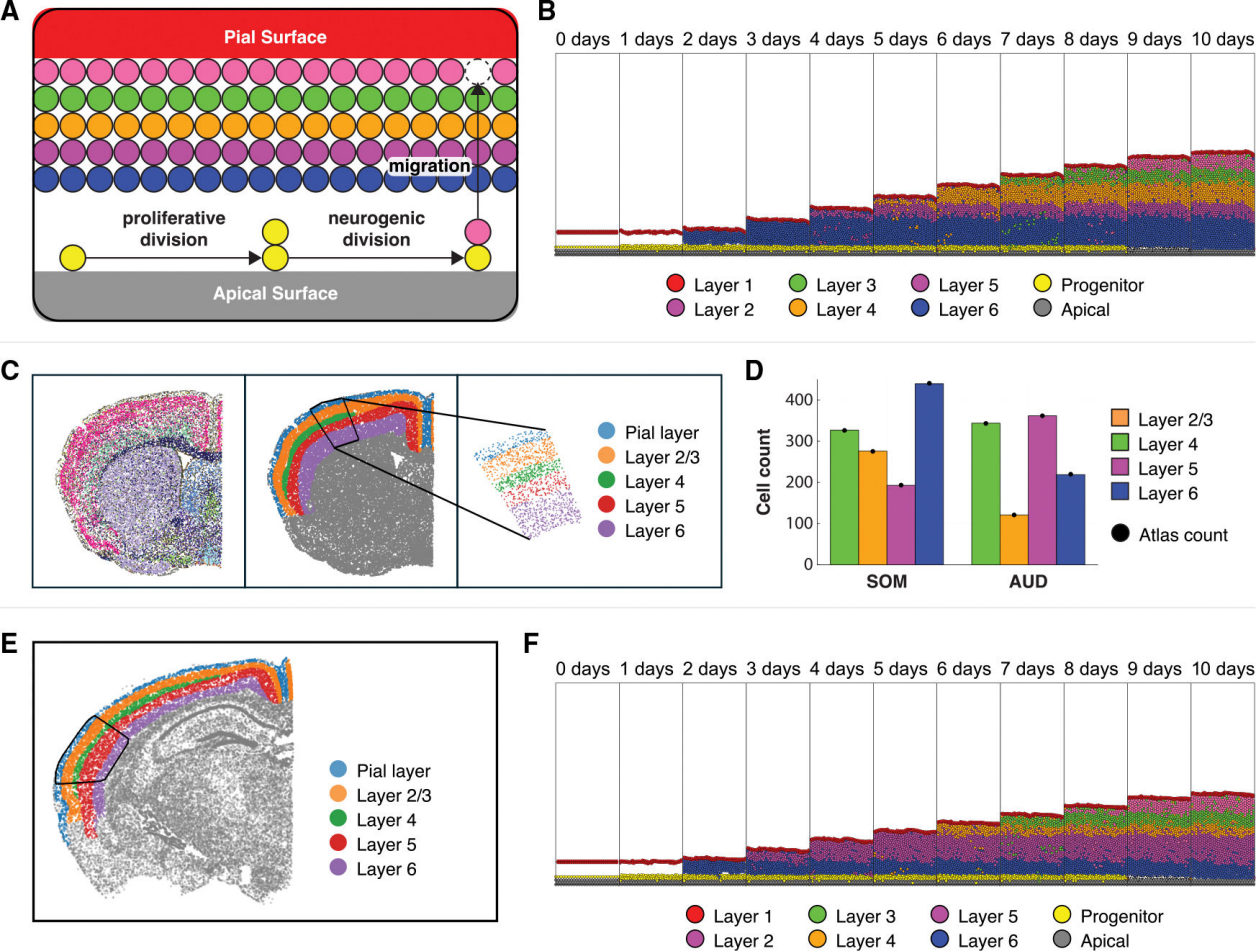
Region-specific laminarization of the cortex (A) Schematic of the formation of the cortical layers in the neuro-development model. (B) Storyboard of the formation of the somatosensory cortex (SOM) after calibration. (C) Extraction of layer counts in the SOM from a single z slice in the Allen Brain Atlas.^53^ (D) Layer counts after calibration for both the SOM and auditory complex (AUD). Black dots represent the counts found from the atlas. (E) Extraction of the layer counts in the AUD from a single z slice in the Allen Brain Atlas.^53^ (F) Storyboard of the formation of the AUD after calibration.

To demonstrate the generalizability of our hypothesis grammar coded in PhysiCell across biological systems, we sought to apply it to simulate layer formation during cortical development in the brain. The laminar organization of different cell types is an archetypal property of vertebrate neural systems. This cytoarchitecture not only replicates across species but across multiple tissues (e.g., retina, hippocampus, cortex) within the central nervous system. Modeling this formation is particularly significant, as disruption and disorganization of the layers is found in both neurodevelopmental and neurodegenerative diseases.^94^ We modeled the formation of this laminar structure by leveraging the rules grammar. While the previous examples in cancer all relied on symmetric cell division, this phenomenon is driven by a combination of symmetric and asymmetric division of progenitors. Specifically, stem cells undergo asymmetric division in which one daughter cell retains its stemness while the other differentiates into a cell fated to a specific layer of the developing brain (Figure 7A). After differentiation, the cells begin migrating toward the pial layer, ending their migration upon contact with the pial layer. The sequence of differentiation into the cortical layers is controlled by the time passed in the neural development using the hypothesis grammar. Additionally, the grammar controls the stem cell division rate, slowing their cycling as time elapses. This in turn increases the overall length of the cell cycle, replicating what is observed *in vivo*. Together with the previously described functionalities, these parameters can generate the cellular diversity and tissue structures prototypical in neural development.

To demonstrate the flexibility of the rules grammar to model neural development, we used the Allen Brain Atlas^53^ to quantify the laminar structure of two regions in the adult mouse brain and calibrate the rules parameters to match each of these regions in turn. We chose the somatosensory cortex (SOM) and the auditory cortex (AUD). Specifically, we used single z slices of the atlas, extracting all cells in that slice from the given region. To quantify the relative abundance of cells within each layer of a given region, we further subset to a rectilinear subspace of the section. Whereas our previous models used spatial data to initialize the model states, here, we fit model parameters to minimize the residual sum of squares of the thickness of the layers at the final time point of the simulation. By fitting the rule parameters to datasets representative of the endpoint of the simulation when the brain regions have fully formed, we are able to successfully produce the laminar structure of both the SOM (representative simulation in Figure 7B, extracted brain atlas data in Figure 7C, and calibrated cell counts in Figure 7D, left) and the AUD (brain atlas data in Figure 7E, compared with a representative simulation in Figure 7F, with calibrated cell counts in Figure 7D, right) in our ABM. These simulations demonstrate that we can use static, spatial multi-omics data to extend beyond model initialization to more complex model calibration for parameter fitting shown here.

## DISCUSSION

The real-world limitations of characterizing the dynamics of cellular and molecular changes in human-focused research, especially for snapshots of spatial multi-omics, do not exist *in silico*. Computational models can guide and supplement lab experiments. For example, the NCI digital twins initiative aims to develop models of patient tumors to predict which therapies will most benefit each individual,^29,31^ by simulating many replicates of their system’s behavior over time and under different sets of conditions. The ability to perform large numbers of replicates and numerous iterations cheaply and easily maximizes the chance of capturing extremely rare critical events. ABMs abstract biological systems to run *in silico* experiments thousands or millions of times, whose parameters and in-built hypotheses are all readily modifiable by the user. The new conceptual framing (a grammar) for specifying cell behavior hypotheses introduced in this study can systemize and facilitate our thinking of how cells interact to drive tissue ecosystems. The grammar made it possible to introduce new capabilities in the PhysiCell ABM framework, thus simplifying the workflow to generate ABMs of multicellular systems.

Previously, custom hand-written code and a high level of technical knowledge were required to implement even basic models. Our hypothesis grammar can encode complex cellular behaviors and responses to signals in single lines of human-readable text. When used in combination with graphical and cloud-based modeling frameworks,^56,95^ the barrier to entry into using ABMs is considerably reduced. In this implementation, it is simple to modulate and apply behaviors to different agents in the system in plain text without requiring writing code or editing machine-readable markup languages. Further, PhysiCell is open-source, community-built ABM software that encodes a vast amount of biological and computational knowledge at baseline; however, everything is completely customizable, extensible, and modifiable. Moreover, this cell behavior grammar affords an opportunity to systematically collect, annotate, curate, and grow our knowledge of cellular behaviors and interactions for use as model templates.^51,96^

We demonstrated a variety of models extending from carcinogenesis and immune response to tumor growth and demonstrating broader extensions to neurodevelopment. Some of these models have all cellular agents following the same rules and fate determined by the actions of those around them. In other models, cell agents act at cross purposes and actively seek to outcompete, evade, or hunt and kill each other. We modeled immune processes such as macrophage plasticity, T cell activation and expansion, antigen recognition, and inflammation. The rules for these examples are all available to be re-run on any user machine, providing sample case studies for new users. These case studies also showcased how ABMs can be applied for *in silico* experimentation of complex multicellular processes, which can prioritize new hypotheses for experimental validation or exploration.

The models in this study also directly translated cellular location and identity from ST data to initialize an ABM. Thus, models can now directly match the tissue structure and transcriptional profile of samples. Spatial relationships between cells and cellular neighborhoods significantly impact outcomes. This strong dependence of many cancer systems and ABM trajectories on initial conditions can complicate model inquiry and impact critical system behaviors and model parameters obtained through inference; by leveraging robust single-cell ST tissue profiles as initial conditions in the digital modeling stage, the hypothesis-driven rules modeling paradigm is grounded in precise referential data but also offers a path to both stronger model inquiry and more confident mathematical inference. These models nonetheless still require annotation of a finite number of agents identified in spatial molecular data, often annotating cells into broad phenotypes and abstracting cellular subtypes. Future work must evaluate the sensitivity of models to the granularity of cellular phenotypes in these high-throughput datasets, accurate inference of parameters in the resulting higher-dimensional models, and curation of best parameter estimates for community reuse of omics-informed ABMs.

The cell-based nature of our mathematical framework can predict the impact of distinct immunotherapeutic combinations on altering the TME. We also showed that these model predictions can be further personalized by inputting baseline cellular abundance measurements, providing a powerful tool for selecting optimal combinations to overcome the immunosuppressive landscape of many solid tumors. Limitations of testing combination therapies experimentally are the large number of experiments required to test ordering, therapies with distinct mechanisms of action, and biological variability of TMEs. The ability of ABMs to simulate systems-level cellular behaviors entirely *in silico* provides an effective means to pre-screen combinations at scale to prioritize therapeutic selection and order of delivery in preclinical and clinical studies. Metrics to benchmark mathematical models both qualitatively and quantitatively against real-world preclinical and clinical studies are essential to fully leverage these models to predict personalized biological conditions. This grammar must also be extended to simulate the pharmacokinetics and pharmacodynamics encoded in more complex quantitative system pharmacology models to fully empower virtual clinical trials.^97^ While our models demonstrated the potential of our software to simulate virtual clinical trials, translating these models to the clinic requires robust calibration and validation of their ability to fully mimic the behavior of human clinical trials. Moreover, the focus of our model on simulating cellular perturbations in local tissue environments limits our predictions to estimating local cellular landscape only, requiring complementary preclinical or clinical studies. We view our framework as ideally suited to prioritize candidate targets for these combinations, still requiring extensive clinical and regulatory evaluation prior to usage as precision medicine tools beyond the scope of this study.

This language framework will be useful to those seeking to build models of multicellular systems, and we are excited to continue to move toward fuller biological completeness and more complete integration with omics data, to increasingly define agent behavior in an automated and a data-driven fashion. These advancements expand the functionality, usability, and compatibility of our approach, empowering interdisciplinary researchers in their computational or systems biology endeavors. These advancements expand the functionality, usability, and compatibility of our approach, empowering researchers across disciplines to unlock the full potential of their single-cell data. Armed with this conceptual framing and tools, they can extrapolate beyond single-cell characterizations for multicellular systems biology and ultimately perform virtual cellular and tissue experiments.

### Limitations of the study

In any computational framework, all required biology must be built from the ground up. The hypothesis grammar for PhysiCell enables many cellular behaviors, but many important aspects remain to be added. In future work, we plan to further refine the hypothesis grammar to expand its usability and flexibility. We are considering incorporating keywords for “wild card” rules (e. g., in all cells, mechanical pressure decreases cycle entry) and other special cases using regular expression-type syntax, as well as extensions (e.g., “low S” or “decreasing S”) that can simplify the examples presented in this paper. Moreover, we plan to add extensions for hysteresis and delayed activation in our responses and for allowing cells to access the properties of contacting cells as signals or inputs to rules (e.g., for delta-Notch signaling or improved antigen recognition). While our software can use multi-omic and spatial molecular data for model initialization and some parameterization, the ability to encode rules directly from inferred regulatory networks and to incorporate uncertainty analyses in the input of these data remains an important area of ongoing research. Moreover, emerging large language models (LLMs) such as ChatGPT may facilitate “translation” of familiar language (e.g., fibrosis) into the smaller set of symbols in the current grammar. The language currently treats all statements as independent (inclusive OR), but we may need additional language operators to signify relationships between rules such as AND or REQUIRES. Other generalizations and improvements to the forms of response curves, consensus process models, and default parameter values are likely to emerge from widespread community use, feedback, and discussion.

Our current grammar is focused on cellular interactions, but it does not yet incorporate gene regulatory networks, although intracellular gene regulatory networks are supported in PhysiCell.^98,99^ While we demonstrated initialization and parameterization from ST data, our software requires further extensions to fully interoperate with omics data analysis ecosystems and emerging high-throughput data modalities. In the models presented here, the connections with the data rely on macroscale summaries of the data such as size of the tumor, cell-type annotations, or thickness of cortical layers. Increasing the depth of connection to the data—spatially, temporally, and phenotypically—will improve the accuracy and predictive power of these models.^100,101^ Further parameter fitting and data assimilation methods are also needed to fully embed experimental data into the models to ensure biologically calibrated ABMs. For example, we note that well-known developmental timings were used to drive the evolution of fate specification in the corticodevelopment example^102,103^ to allow us to focus on key transitions responsible for final cytoarchitecture; future work can integrate Boolean networks^99^ or systems of ODEs to replace time as a proxy signal. A key component of this endeavor is to uncover the roles of all cells in the complex interaction network within any given system and the effect of therapeutic perturbations thereon. In this study, the various models include only a subset of the cell types known to exist in the modeled microenvironments and apply simplified frameworks of pharmacodynamic response. A limitation of our approach is that it relies on cataloguing individual cell types and their behaviors,^51,96^ although future work can leverage artificial intelligence to extend beyond manual cataloging by automating discovery with expert quality control. Additionally, future work will establish a community-informed repository to collect and curate biological hypothesis statements grouped as digital cell lines,^51,96^ enabling users to contribute and share cell behavior statements for future reuse in other models of the same system. Continued community input will expand and refine digital cell templates and phenotypic behaviors to actualize virtual cell laboratories.

## RESOURCE AVAILABILITY

### Lead contact

Further information and requests for resources and reagents should be directed to and will be fulfilled by the lead contact, Paul Macklin (macklinp@iu.edu).

### Materials availability

This study did not create any new materials.

### Data and code availability

- PhysiCell Version 1.14.1^104^ and later includes a full reference implementation of the grammar and grammar-based simulation modeling, and the specific models in the results are available from https://github.com/physicell-models/grammar_samples.
- Raw data from the cell motility experiment in Figure 5G are provided at https://zenodo.org/records/14106341. Spatial transcriptomics data of PDAC tumors are available from GEO: GSE254829. scRNA-seq data from PDAC tumors are available from GEO: GSE155698. Allen Brain Atlas datasets used in this publication are available from https://knowledge.brain-map.org/data/5C0201JSVE04WY6DMVC/summary. All other datasets are available from the authors upon request. The simulation sensitivity analysis data are available at https://doi.org/10.5281/zenodo.14590311.

## STAR★METHODS

### METHOD DETAILS

#### Simulation Methods

##### PhysiCell agent-based modeling framework

PhysiCell^47^ is an open source, agent-based modeling framework written in C++ that can run on a broad variety of desktop platforms, in the cloud,^111^ and on high performance computing resources.^38–40,112^ PhysiCell simulates each cell as an agent with lattice-free position and volume, individual birth and death rates, and motion driven by the balance of mechanical forces and biased random migration. In more recent versions of PhysiCell, agents can also interact with built-in models of phagocytosis, effector attack, fusion, and elastic cell-cell adhesion. PhysiCell is coupled to a reaction-diffusion solver (BioFVM^113^) that models secretion and uptake (consumption) of diffusible factors by individual cell agents at their individual positions, as well as diffusion and decay of these substrates through extracellular spaces. PhysiCell bundles its key cell behavioral parameters as a *phenotype object* for simpler representation. Modelers simulate biological hypotheses by writing custom C++ functions that dynamically vary the cell agent’s phenotype parameters based on conditions at the cell’s position, such as contact with other cells, mechanical pressure, and concentrations and gradients of signaling factors. This paper extends PhysiCell with built-in functions that parse rules written with our grammar to operate on cell phenotypes without writing C++ code.

##### Installation instructions

PhysiCell Version 1.14.1^104^ and later includes a full reference implementation of the grammar and grammar-based simulation modeling, and the specific models in the results are available from https://github.com/physicell-models/grammar_samples. To get a list of all the example models:

make list-user-projects

To load and compile an example named **myproject**, use

make load PROJ=myproject && make

Similar to our prior work to create cloud-based training materials^114^ and cloud-based model dissemination,^111^ and inspired by other recent advances on “zero-install” models,^115^ we have created a cloud-based version^56,116^ of PhysiCell based on the nanoHUB platform.^105^ This cloud implementation allows scientists to create, execute, visualize, and explore grammar-based models interactively in a web browser, without need for programming expertise or software setup. (See documentation and training materials in Methods S1) The cloud-hosted model is available at https://nanohub.org/tools/pcstudio. Alternatively, scientists can download the latest release of the PhysiCell Studio^56^ desktop application at https://github.com/PhysiCell-Tools/PhysiCell-Studio/releases. Assuming an executable model has been compiled, the Studio allows interactive creation and editing of rules, running a simulation, and visualizing results. Refer to the Studio user guide at https://github.com/PhysiCell-Tools/Studio-Guide/blob/main/README.md for more information.

Self-guided, hands-on training courses are available at https://physicell.org/Training.html. See further details in Methods S1.

#### Hypothesis grammar

##### Cell behaviors

To build this grammar, we require clear abstractions of key cell behaviors that frequently occur in multicellular observations and corresponding reference models. In this context, a ***cell behavior*** is a cell-scale process, such as cycling, death, or phagocytosis. Generally, each behavior can be represented by a small number of continuous phenotypic parameters, describing the rate, magnitude, or frequency of the behavior. In earlier work, Sluka et al. developed the Cell Behavior Ontology (CBO)^117^ as a controlled vocabulary of individual cell behaviors. More recently, we worked with a multidisciplinary coalition to extend and structure behaviors from the CBO and other sources into MultiCellDS^96^ (multicellular data standard). In particular, this work defined a *cell behavioral phenotype* that collects of biophysical characterizations of a cell’s behavior, organized hierarchically by function: cycling, death, volume, mechanics, secretion (including uptake), and motility. Since releasing MultiCellDS as a preprint, we have tested this approach to cell behavior through a variety of agent-based simulation and modeling projects.^38–40,47,54,58,99,114,118–122^ Based upon recent immunologic modeling work,^38,118–121^ we extended phenotype to include cell-cell interactions (phagocytosis, effector attack, and fusion), as well as transitions between cell types (e.g., differentiation, transdifferentiation, and other state changes that persist even when exogenous signals are removed). See Methods S1 for a full description of these cell behaviors, including reference model implementation details in the PhysiCell framework.

##### Signals

Signals are (typically exogeneous but sometimes internal) stimuli or information that can be interpreted by a cell to drive behavioral or state changes. In the context of mathematical modeling, signals are inputs to constitutive laws or agent rules. We broadly surveyed mathematical and biological models from cancer biology,^36,61,123–132^ tissue morphogenesis,^123,133–137^ immunology,^36,118–120,138,139^ and microbial ecosystems,^140,141^ to generalize classes of inputs to cell behavioral rules, generally including chemical factors, mechanical cues, cell volume (e.g., for volume-based cycle checkpoints), physical contact with cells, live/dead status, current simulation time (for use in triggering events), and accumulated damage (e.g., from effector attack^142–144^). See Methods S1 for a full description.

##### Behavioral statements

For any cell type T, we construct simple statements that relate changes in a single behavior B to a signal S: “In T, S increases/decreases B [with optional arguments].” Here B is a well-defined biophysical parameter in our dictionary of behaviors (see STAR Methods and Methods S1), S is a well-defined biophysical variable in our dictionary of signals, and optional arguments further specify the mathematical behavior of the responses. For example:

In MCF-7 breast cancer cells, cisplatin increases apoptosis.

In naïve T cells, IL-10 decreases transition to CD8^+^ T cells.

A full description of the grammar, optional arguments, and examples can be found in Methods S1.

##### Mathematical representation: individual rules

With clearly defined behaviors and signals and the grammar to connect them, we can uniquely map human-interpretable cell hypothesis statements onto mathematical expressions that make the grammar both human interpretable and computable. Each individual rule modulates a single behavioral parameter b as a function of a signal s. Given a response function R, we then mathematically represent the individual rule as a function b(s):

(1) $$b(s)=b_{0}+\left(b_{M}-b_{0}\right)R(s),$$

where $b_{0}$ is the base value of the parameter in the absence of signal, and $b_{M}$ is the maximally changed value of the parameter with large signals. By default, we use sigmoidal (Hill) response functions R, due to their extensive use in signaling network models and pharmacodynamics, as well as their smooth variation between 0 (at no response) and 1 (at maximum response). However, capped linear response functions (varying between 0 and 1) and step functions are also possible (Figure S2). See Figure 1C for a typical rule. Full mathematical details and additional detailed examples are available in Methods S1.

##### Generalized mathematical representation: multiple rules

Our full mathematical formulation allows new hypotheses to be directly added to models without modifying prior hypotheses, making our framing extensible and scalable as new knowledge is acquired. Suppose that a behavior B (with corresponding behavioral parameter b) is controlled by multiple rules subject to promoting (up-regulating) signals u and inhibiting (down-regulating) signals d:

- $u_{1}$ increases B (with half-max $u_{1}^{*}$ and Hill power $p_{1}$)
- $u_{2}$ increases B (with half-max $u_{2}^{*}$ and Hill power $p_{2}$)
  …
- $u_{m}$ increases B (with half-max $u_{m}^{*}$ and Hill power $p_{m}$)
- $d_{1}$ decreases B (with half-max $d_{1}^{*}$ and Hill power $q_{1}$)
- $d_{2}$ decreases B (with half-max $d_{2}^{*}$ and Hill power $q_{2}$)
  …
- $d_{n}$ decreases B (with half-max $d_{m}^{*}$ and Hill power $q_{n}$)

Here, let $b_{M}$ be the maximum value of the behavior parameter b (under the combined influence of the up-regulating signals u), let $b_{0}$ be its base value in the absence of signals, and let $b_{m}$ be its minimum value (under the combined influence of the down-regulating signals d).

Similar to prior multi-variate response functions,^145,146^ we define the total up response as:

(2) $$U=H_{M}\left(u;u_{\text{half}},p\right)=\frac{{\left(\frac{u_{1}}{u_{1}^{*}}\right)}^{p_{1}}+{\left(\frac{u_{2}}{u_{2}^{*}}\right)}^{p_{2}}+\ldots +{\left(\frac{u_{m}}{u_{m}^{*}}\right)}^{p_{m}}}{1+{\left(\frac{u_{1}}{u_{1}^{*}}\right)}^{p_{1}}+{\left(\frac{u_{2}}{u_{2}^{*}}\right)}^{p_{2}}+\ldots +{\left(\frac{u_{m}}{u_{m}^{*}}\right)}^{p_{m}}}$$

and the total down response as:

(3) $$D=H_{M}\left(d;d_{\text{half}},q\right)=\frac{{\left(\frac{d_{1}}{d_{1}^{*}}\right)}^{q_{1}}+{\left(\frac{d_{2}}{d_{2}^{*}}\right)}^{q_{2}}+\ldots +{\left(\frac{d_{n}}{d_{n}^{*}}\right)}^{q_{n}}}{1+{\left(\frac{d_{1}}{d_{1}^{*}}\right)}^{q_{1}}+{\left(\frac{d_{2}}{d_{2}^{*}}\right)}^{q_{2}}+\ldots +{\left(\frac{d_{n}}{d_{n}^{*}}\right)}^{q_{n}}}.$$

We combine the overall response of the behavioral parameter via bilinear interpolation in the nonlinear up- and down-responses U and D:

(4) $$b(u,d)=(1-D)\cdot \left[(1-U)\cdot b_{0}+U\cdot b_{M}\right]+D\cdot b_{m}$$

Notice that:

- In the presence of a single up-regulating signal u (or a single down-regulating signal d) only, b(u,d) reduces to a Hill response curve b(u) (or b(d)) used in systems biology and pharmacodynamics studies.
- Generally, the combined up-regulating signals sets a “target” value of the parameter, which can then be inhibited by the combined down-regulating signals.
- Both U and D vary between 0 and 1 representing the extent of up- and down-regulating signals, respectively. This means that larger values of D represent larger decreases in behavior b.

Note also that adding and removing individual rules does not require alteration to the remaining rules. In this release, we use multivariate Hill response functions for clarity, but mixed linear and Hill responses could be used in the future. The PhysiCell implementation of this generalized response, additional mathematical details, and expanded examples can be found in Methods S1. Sample multivariate response functions are in Figures S1 and S3.

###### PhysiCell rules implementation and parameterization.

To implement these rules in PhysiCell, users generate a CSV file in which each row is an individual rule and the columns correspond to specific elements of the grammar. The structure of such a row is as follows:

$$\underset{\text{cell type}}{\underset{︸}{\text{tumor}}},\underset{\text{signal}}{\underset{︸}{\text{oxygen}}},\underset{\text{response}}{\underset{︸}{\text{increases}}},\underset{\text{behaior}}{\underset{︸}{\text{cycle entry}}},\underset{\text{maxresponse}}{\underset{︸}{0.0005}},\underset{\text{half-max}}{\underset{︸}{5.0}},\underset{\text{Hill power}}{\underset{︸}{4}},\underset{\text{applies to dead?}}{\underset{︸}{0}}$$

The graphical user interface (GUI) provided by PhysiCell Studio^56,95,116^ simplifies the creation of this CSV in alignment with the framework. Within this GUI, users can also interactively visualize all the rules to assess their sensitivity to different input signals and parameter values.

Our toolset also includes a Python package to analyze PhysiCell models, including sensitivity analysis, calibration, model selection, and validation. These uncertainty quantification (UQ) tasks are critical for understanding how biological and mathematical variability influence model behavior. Importantly, the addition of the grammar framework enables us to offer this to end users without requiring bespoke C++ code or XML parsers. More details on these parameter tuning and parameter sensitivity tools are described in detail in Methods S1. To help drive reproducibility, we generate and save a full description of all rules in HTML and text formats after initial parsing.

#### Experimental details

##### Macrophage co-culture with 3D mammary organoids derived from MMTV-PyMT tumors

A previously unpublished replicate image of co-culture of organoids with tumor-associated macrophages is used as the basis of the qualitative behavior of our ABM of macrophage-induced invasion of tumor cells. Experimental methods for the data generated in this figure are described in the original DeNardo et al. publication^84^ describing these findings as follows “Primary nMEC and pMEC pools were established by organoid centrifugation as previously described.^152^ Briefly, mammary tissue biopsies were harvested from 76-day-old PyMT female or 12-week-old virgin negative littermates and digested with collagenase A 2.0 mg/ml (Roche) and DNase 2.0 units/ml (Roche) for 2 hr. Organoids were then isolated by differential centrifugation and grown in culture conditions as previously described.^152^ Primary nMECs were used within two passages and primary pMEC cells were used within ten passages. Three-dimensional organotypic cultures were established as previously described.^153^ Cultrex basement membrane extract (BME; R&D Systems) was utilized to limit endotoxin levels. Co-cultures with primary leukocytes were established only after stable organoid structures had formed (approximately 3 weeks for nMEC, 2 weeks for pMEC). Leukocytes were overlaid in medium containing 0.5% BME. Formation of invasive acini was assessed every 12 hr for 3 days. The cytokines IL-4 (20 ng/ml), IL-13 (20 ng/ml), IL-10 (10 ng/ml), IFNg (5.0 ng/ml) (PeproTech), or LPS (1.0 mM/ml) were added to co-cultures 12 hr after leukocytes overlay. Inhibitors PD153035 (0.1 mM, Calbiochem) or BIBX1382 (10 nM, Calbiochem) were added 1.0 hr prior to the addition of leukocytes. All experiments were repeated two or three times with separate pMEC pools and individual experiments were run at least in triplicate.

##### Invasion and colony formation assays of gefitinib treated organoids derived from MMTV-PyMT tumors

Primary mammary tumor organoids were isolated from female MMTV-PyMT mice (002374; Jackson Laboratory) using sequential digestion and purification steps as previously described.^154^ Briefly, tumors were dissected, divided with a scalpel, and shaken in a collagenase digestion solution for 1 hour at 37 °C. Following digestion, a series of differential centrifugations were used to separate epithelial organoids from stromal cells, with resulting organoids between 100–250 cells in size. For invasion assays, organoids were embedded at a density of 1.2 per μL into a collagen I extracellular matrix (354236; Corning) in glass bottom imaging plates (662892; Grenier). The ECM was then polymerized for 1 hour at 37 °C, after which DMEM/F-12 media (10565-018; Thermo Fisher) containing 1.0% ITS (51500-056; Thermo Fisher), 1.0% Pen-Strep (P4333; Sigma Aldrich), and 2.5 nM FGF2 (F0291; Sigma Aldrich) was added. Compounds dissolved in DMSO were added after overnight incubation, and assays were then incubated for 96 hours at 37 °C with 5.0% CO2. Cultures were fixed in 4.0% paraformaldehyde (15714-S; Electron Microscopy Sciences) on day 5 and then imaged on laser scanning confocal microscope equipped with a tunable GaAsp detector, 2k resonant scanner, and LUA-S6 laser unit (AXR; Nikon Instruments). Invasion was assessed by calculating the inverse circularity of each organoid using Nikon NIS-elements software and results are normalized per biological replicate.

For colony formation assays, organoids were further digested to cancer cell clusters (2–10 cells in size) using 1X TryPLE (12604-013; Thermo Fisher). Clusters were then isolated through differential centrifugations as previously described^154^ and embedded at a density of 100 per μL in Matrigel (354230; Corning). After the Matrigel had polymerized, media was added and compounds in DMSO were dosed the following day using a D300e Digital Dispenser (Tecan). The assay was then incubated at 37 °C with 5.0% CO2 and fixed after 96 hours in 1.0% paraformaldehyde. To determine colony formation, the entire ECM was imaged in 3D, maximum intensity projections were generated, and colonies were counted using custom ImageJ and Python scripts. Percent colony formation was calculated as the number of colonies in each treatment condition normalized to the vehicle control.

##### Growth and motility of EGF treated MCF10A cells

As described previously,^155^ MCF10A cell culture and experimental procedures were conducted based on established methodologies.^156^ For routine maintenance and passaging, cells were cultured in a growth medium composed of DMEM/F12 (Invitrogen, #11330-032) supplemented with 5% horse serum (Sigma, #H1138), 20 ng/ml EGF (R&D Systems, #236-EG), 0.5 μg/ml hydrocortisone (Sigma, #H-4001), 100 ng/ml cholera toxin (Sigma, #C8052), 10 μg/ml insulin (Sigma, #I9278), and 1% Penicillin/Streptomycin (Invitrogen, #15070-063). For experiments involving EGF perturbation, a growth factor-free medium was prepared using DMEM/F12, 5% horse serum, 0.5 μg/ml hydrocortisone, 100 ng/ml cholera toxin, and 1% Pen/Strep.

Cells were cultured to 50–80% confluency before being detached with 0.05% trypsin-EDTA (Thermo Fisher Scientific, #25300-054). Subsequently, 20,000 cells were seeded into 24-well plates (Thermo Fisher Scientific, #267062) coated with collagen-1 (Cultrex, #3442-050-01) in growth medium. After six hours, the cells were rinsed with PBS, and the medium was replaced with growth factor-free medium. Following an 18-hour period of growth factor deprivation, cells were treated with either PBS or 10 ng/ml EGF (R&D Systems, #236-EG).

Phenotypic responses to EGF treatment were assessed through live-cell imaging using the Incucyte S3 microscope (Essen BioScience, #4647), which captured images every 30 minutes over a 24-hour period. The dataset includes an Excel spreadsheet that documents the experimental conditions for each imaged well.

##### PDAC patient-derived tumor spheroid CAF co-culture invasion assay

###### Samples Acquisition.

Patient-derived organoids (PDOs) and cancer-associated fibroblasts (CAFs) were isolated from freshly resected pancreatic ductal adenocarcinoma (PDAC) tumor specimens obtained during pancreatectomy procedures at Johns Hopkins University Hospital. All specimens were processed within 24 hours of surgical resection as previously described for pancreatic cancer organoids.^157^ Written informed consent was obtained from all patients prior to sample collection.

###### Organoid Generation.

PDAC tissue samples were rinsed, minced, and digested in a digestion medium containing Dispase (Gibco 17105-041) and Collagenase Type II (Gibco 17101-015) at 37°C for 2–3 hours with 200 rpm shaking. The digested suspension was centrifuged (1500 rpm, 5 minutes, 4°C) and washed multiple times with wash media (DMEM/F-12 supplemented with 1.25 mL Primocin, 5 mL 1M HEPES, 5 mL 100X Glutamax, and 2.5% FBS). Tumoral clusters and single cells were separated from stromal debris and CAFs through differential centrifugation. Organoids were embedded in collagen I gel prepared by mixing collagen I (Corning, rattail, 354236) with 10× DMEM and 1N NaOH to achieve a final concentration of 3.34 mg/mL. The collagen solution was incubated at 37°C for 60 minutes to allow polymerization before overlaying with growth media containing DMEM/F-12, Primocin, HEPES, Glutamax, EGF (5 ng/mL), insulin (5 μg/mL), cholera toxin (10 ng/mL), and BSA (0.075%). CAF-containing supernatants were also cultured separately in T75 flasks with RPMI containing 10% FBS. PDO growth was monitored over two weeks to reach an appropriate size, and cultures that failed to expand were discarded.

##### CAF Media Treatment and Invasion Assays.

CAFs were cultured in 2D (T75 flask) and 3D (Matrigel in 24-well plates) environments to induce differentiation into inflammatory (iCAF) and myofibroblast (myCAF) subtypes, respectively. Fibroblast differentiation was validated using qPCR with iCAF and myCAF markers. Once the cells reached 70–80% confluence, the media were replaced with RPMI containing 1% FBS. Conditioned media were collected after 36 hours and used for all 15 PDO cultures. CAF-conditioned media were mixed with PDO growth media at a 1:1 ratio and RPMI with 1% FBS was used as a control. To assess invasion, images of invasive and non-invasive organoids were captured using a Nikon Ti-E inverted microscope at ×10 magnification 72 hours after conditioned media addition. Organoid invasion was quantified by calculating the percentage of PDOs invading collagen fibers and analyzing invasive organoids’ circularity using ImageJ software.

##### Cell motility tracking of PDAC cells and fibroblasts in varying ECM densities, alone and in co-culture

We embedded cells from the hT231 human cancer associated fibroblast cell line and the Panc10.05 pancreatic cancer cell line,^158^ either separately or in co-culture, into 3D collagen-I hydrogel and imaged individual cells at five-minute intervals for eight hours. The cell tracking protocol was performed as previously described.^68^ Briefly, the Panc 10.05 and hT231 CAF co-cultures were prepared in type I collagen-based gels that were polymerized for 1hr in a 37-degree incubator (during which time the gels turned from a liquid to form a stable solid). At the end of the 1hr polymerization time, the gels are deemed solid/stable and are all gently hydrated with media to keep them porous and feed the cells with nutrients, then placed in the cell culture incubator for approximately 2 hours prior to imaging. The gels (monocultures and co-cultures both) were then loaded onto our microscope and imaged soon after (within 2–3 hours of hydrating), then were run in an overnight movie that elapsed a total time of about 12–16 hours. The trajectories in the .csv files quantified cell movements over either 2.5hr (30 frame)-long or 8hr (96 frame)-long trajectories within this 12-hour movie.

##### Calibration of migration speed in ABM from motility assays

The 3D positions (x, y, z) of each cell—as recorded by the microscope at each timestep—were analyzed, and the motility of each cell was fitted to a trajectory using the anisotropic random walk model described previously,^68,159^ which yields metrics such as average speed (μm/hr) for each condition.A protocol for statistical analysis of cell migration in 3D was used to calibrate an anisotropic persistent random walk model.^160^ This was performed at each collagen density used in the 2.5h motility assays. The average speed parameter was taken from this analysis as a function of the collagen density. The combined Hill response (see Results) is fit to this data. ECM density is used as the increasing signal and the decreasing signal. MATLAB’s fmincon was used to minimize the sum of square residuals and thus parameterize the Hill responses. The code is available in the GitHub repository associated with this article at https://github.com/PhysiCell-Models/grammar_samples/tree/main/experimental_data_analysis/PancCAFAnalysis.

### QUANTIFICATION AND STATISTICAL ANALYSIS

#### Model initialization from simulated distributions of cellular states and from multi-omics data

Another critical model input is the initialization of the cell types present in an ABM simulation and the initial positions of these cell agents. PhysiCell allows users to initialize cells randomly in the environment or by supplying a user-created file with cell locations. An advantage of the hypothesis grammar is that cellular agents are given human-interpretable names, providing a one-to-one mapping between the agents and cellular labels defined in classical single-cell and spatial molecular analyses. To leverage this mapping and enable data-driven model initialization, we use cell type annotations from bioinformatics datasets to set the relative abundances of the cell types included in our models. If the data also includes spatial coordinates, we use affine linear transformations to position the cells in the simulation domain. By default, the cells are placed to fill the simulation domain while preserving the aspect ratio of the data.

#### Spatial transcriptomics data of PDAC tumors

We selected two resected pancreatic lesions were subjected to the commercial Visium spatial transcriptomics (ST) sequencing FFPE protocol generated in Bell et al.^72^ to initialize ABMs in this study. Slides were stained with H&E and imaged prior to RNA extraction, and image analysis was performed in parallel with transcriptomic analysis. An artificial intelligence method for annotation of pancreatic tumor tissue regions called CODA^106^ was used to annotate acinar cells, islet cells, smooth muscle cells, and the distribution of collagen. This method was also used to distinguish normal ductal, neoplastic, and tumor cells from the H&E imaging, which were further visually confirmed by a pathologist (E.D.T.). Spots with greater than 70% purity of ductal cells were further annotated to assign agent types for the associated tumor and normal cells in each spot. For this annotation, we used our transfer learning method ProjectR^107^ version 1.8.0 to distinguish proliferative signaling (modeled as an epithelial phenotype) from co-occurrence of EMT and inflammatory signaling (modeled as the mesenchymal phenotype) as defined in CoGAPS non-negative matrix factorization analysis of in scRNA-seq data PDAC tumors using methods described previously.^72,108,109^ To locate fibroblasts, Seurat version 4.1.0 was used to compute module scores from a pan-CAF gene signature as described previously.^72^ ABM simulations were initialized from these cellular states. In addition, ECM density was initialized using a heuristic from image-derived collagen and cancer-associated fibroblast annotations from the H&E imaging from CODA,^106^ and the bounding cells were assumed to contain a similarly dense collagen matrix, forming a niche around the known sample and abstractly reflecting the character of the solid pancreatic tissue. In the ABM, other pancreatic cells in the spatial transcriptomics data were approximated as steady state (no net proliferation, death, motility, or secretion) and were assumed to be essentially inert with regards to carcinogenesis, here primarily modeled in their role as structure and scaffolding within which the other cell types interact and representing acinar cells, islet cells, and smooth muscle cells. The spatial transcriptomics data^147,148^ are available from GEO as GSE254829.

#### Deriving a metric to quantify the invasiveness of in silico tumor spheroids over time from in silico simulation (PhysiCell model output)

Previous studies have quantified the invasiveness of tumor spheroids using microscopy images by assessing the perimeter (contour) of the spheroid and counting cells migrating beyond a defined booundary.^149,150^ These methods were adapted to enable comparison between output from *in silico* PhysiCell models. To quantify invasiveness, the radial distance from the perimeter of the boundary of the simulated tumor volume to the centroid of its contour was calculated. Projections of the spheroid’s perimeter extending beyond the median radial distance measured at the initial time point were counted as invasive projections. The total number of invasive projections served as a measure of spheroid invasiveness. Results from simulation experiments of tumor spheroids in PhysiCell were processed and quantified using a custom analytic pipeline^151^ written in Python (version 3.11). The positions of cells in the ABM at each time point were imported from xml output files produced by PhysiCell as MultiCellDS (MCDS) data structures in Python and used to populate an empty array as individual points. Points were then dilated in the shape of a disk with a gradient of intensity. The simulated outputs contain both images of the secreted factors from fibroblasts, abstracted as an extracellular matrix (ECM) variable, and tumor cells. The locations of the ECM were then imported from the MCDS object, and the amount of ECM present at each voxel in the simulation space was used to derive a contour by projecting onto a 2D mesh. The ECM contour was added to the dilated cells and flattened into a 2D array. Arrays were processed analogously to images of tumor volume, by binarization with an Otsu threshold and segmented to generate a mask. The morphology of mask contours was then quantified to determine the number of invasive projections and determine spheroid invasiveness.

#### Single-cell RNA-seq of PDAC tumors as a reference dataset to initialize estimates of immune populations in ABM simulations of treatment effects

Simulations of different treatment effects on PDAC in Figure 6 use immune-enriched single-cell RNA-seq data from Stelle et al.^92^ (GSE155698) and with preprocessing to further define immune cell subtypes in reference tumors as described previously.^67,91^ Briefly, to determine the by-tissue cell counts to initialize our simulations, the single-cell RNA sequencing data was preprocessed, clustered, and annotated using the Seurat R package.^110^ Cell identity clusters of interest (“Activated_CD4”, “B cell”, “CD4”, “CD8”, “Effector_CD8”, “Epithelial_cancer”, “Macrophage”, “Mast”, “Neutrophil”, “NK/CTL”, “T cell”, “Treg cell”) were then thresholded based on median normalized expression of genes of interest (here CD274/PD-L1, PDCD1/PD-1, TNFRSF9/CD137) and the number of cells falling into lo/hi categories were reported as described previously.^91^ The pre-treatment cell numbers for each immune population were adopted as the baseline count of that agent type, while the number of tumor cells was artificially adjusted to equal 1000, with the relative number of PD-L1^hi^ vs PD-L1^lo^ tumor cells determined by the ratio found in that tissue in the dataset.^92^ Cell-cell communication analysis was performed using the Domino package as described previously by Li et al.^91^ to extend the model rules for immune cells to account for their function dependent on CD137 status. We further estimate the relationship of the baseline TME composition on the ABM-simulated therapeutic response with a Pearson correlation computed with the R package ggpubr.

#### Allen Brain Atlas^53^ data as a reference dataset to calibrate parameters of an ABM of cortical development

The Allen Brain Atlas^53^ was used to find z-slices of cortical regions in the mouse brain and the cellularity in each layer to calibrate the model. We selected z-slices that contained sufficient cellularity in the two chosen regions of interest, the somatosensory and auditory cortices. Selecting from each z-slice a rectilinear subset within the target region, we used the Allen Brain Atlas layer annotations to quantify the thickness of each layer.

### EXPERIMENTAL MODEL AND STUDY PARTICIPANT DETAILS

This study involved no human subjects, and it developed agent-based simulations that form *in silico* simulations of tumors. All human genomics datasets were taken from prior studies. The PyMT mouse model and MCF10A were used for breast cancer, and patient derived organoids, Panc10.05, and hT231 cell lines were used for pancreatic cancer experimental models. Details of all of these and protocols are described in the experimental methods section of STAR Methods.

## Supplementary Material

1

2

3

4

5

6

7

8

9

10

11

12

13

14

15

16

Supplemental information can be found online at https://doi.org/10.1016/j.cell.2025.06.048.

## Figures and Tables

**Table:** KEY RESOURCES TABLE

REAGENT or RESOURCE	SOURCE	IDENTIFIER
Biological samples		

PDAC tumor specimens	Johns Hopkins Hospital	Not applicable
Patient-derived organoids (PDOs)	This Study	Not applicable
Cancer-associated fibroblasts (CAFs)	This Study	Not applicable

Chemicals, peptides, and recombinant proteins		

Gefitinib	MedChemExpress	Cat#HY-50895
PD153035 inhibitor	Calbiochem	23-449
IBIBX1382 inhibitor	Calbiochem	324832
IL4	PeproTech	214-14
IL13	PeproTech	210-13
IL10	PeproTech	AF-210-10
IFNγ	PeproTech	315-05
LPS	Invitrogen	00-4976
Collagenase A	Roche	11088793001
DNase I	Roche	10104159001
Cultrex BME	R&D Systems	3431-005-01
Recombinant Human EGF Protein, CF	R&D Systems	236-EG
Rat tail collagen type I	Corning	354236
Dispase	Gibco	Cat #17105-041
Collagenase Type II	Gibco	Cat #17101-015
Collagen I (rat-tail)	Corning	Cat #354236
Human EGF	Sigma-Aldrich	E9644
Insulin	Gibco	Cat #12585
Cholera toxin	Sigma-Aldrich	C8052
Bovine Serum Albumin (BSA)	Sigma-Aldrich	A1595
Matrigel	Corning	Cat #: 354236

Critical commercial assays		

Cell tracker	Invitrogen	C7025

Deposited data		

Live-cell imaging from EGF or PBS treated MCF10A cells	Zenodo	https://zenodo.org/records/14106341
Sensitivity analysis simulation data	Zenodo	https://doi.org/10.5281/zenodo.14590311

Experimental models: Cell lines		

Panc10.05	ATCC.org	CRL-2547
hT231	Lab of Dr. David Tuveson	N/A
Mouse: FVB/N-Tg(MMTV-PyVT)634Mul/J	The Jackson Laboratory	Cat#002374;RRID: IMSR_JAX:002374
Cell Line: MCF10A	Gift from Gordon Mills (OHSU)	N/A

Software and algorithms		

NIS-Elements	Nikon Instruments	https://www.microscope.healthcare.mkon.com/products/software/nis-elements
ImageJ	NIH	https://imagej.net/ij/
Baxtor Algorithm	https://doi.org/10.1109/TMI.2014.2370951	N/A
CellPose 2.0	https://doi.org/10.1038/s41592-022-01663-4	N/A
PhysiCell	Ghaffarizadeh et al. (2018)^47^	http://physicell.org/
PhysiCell Studio	Heiland et al.^56^	https://nanohub.org/tools/pcstudio
nanoHUB platform	Madhavan et al.^105^	https://nanohub.org/
Computational model in C++	This paper	https://github.com/physicell-models/grammar_samples
Migration speed calibration code	This paper	https://github.com/PhysiCell-Models/grammar_samples/tree/main/experimental_data_analysis/PancCAFAnalysis
Uncertainty quantification software	This paper	https://github.com/heberlr/UQ_PhysiCell
Spheroid analysis software	This paper	https://github.com/emcramer/abm-spheroid-invasiveness/releases/tag/v0.1.0-beta
CODA	Kiemen et al.^106^	https://doi.org/10.1038/s41592-022-01650-9
ProjectR transfer learning software	Sharma et al.^107^	https://doi.org/10.1093/bioinformatics/btaa183
CoGAPs non-negative matrix factorization for scRNA-seq data	Bell et al.^72^, Johnson et al.^108^, Kinny-Köster etal.^109^	https://doi.org/10.1038/s41596-023-00892-x
Seurat 4.1.0	Hao et al.^110^	https://github.com/satijalab/seurat/releases

## Highlights

- Democratize computing by encoding complex multicellular dynamics in plain language
- Framework digitizes tumor microenvironment dynamics, neurodevelopment, and more
- Personalize, parameterize, and validate virtual cell lab experiments using multi omics
- *In silico* hypothesis testing links theory to data accelerating biological discovery
