# Supplementary material for: Human interpretable grammar encodes multicellular systems biology models to democratize virtual cell laboratories
Source: Cell. Author manuscript; Available in PMC 2026 Mar 24. (PMC13012569; doi:10.1016/j.cell.2025.06.048)
Supplement: 14 [file NIHMS2100468-supplement-14.pdf]

Human interpretable grammar encodes multicellular systems biology models to democratize virtual cell laboratories: Methods S1

Jeanette A.I. Johnson[1,2+], Daniel R. Bergman[1,2,3,4,5,6+], Heber L. Rocha[7], David L. Zhou[8], Eric Cramer[9], Ian C. Mclean[9], Yoseph W. Dance[10,11], Max Booth[1], Zachary Nicholas[8,12], Tamara Lopez-Vidal[1], Atul Deshpande[1,2,13,14], Randy Heiland[7], Elmar Bucher[7], Fatemeh Shojaeian[15], Matthew Dunworth[16], André Forjaz[17], Michael Getz[7], Inês Godet[18], Furkan Kurtoglu[7], Melissa Lyman[1,2], John Metzcar[7,19], Jacob T. Mitchell[1,2,12], Andrew Raddatz[20], Jacobo Solorzano[21], Aneequa Sundus[7], Yafei Wang[7], David G. DeNardo[22], Andrew J. Ewald[1,2,16,23], Daniele M. Gilkes[1,11,17], Luciane T. Kagohara[1,2], Ashley L. Kiemen[1,2,15], Elizabeth D. Thompson[15], Denis Wirtz[1,2,15,17,24], Laura D. Wood[1,15], Pei-Hsun Wu[2,17], Neeha Zaidi[1,2], Lei Zheng[1,2,25,26], Jacquelyn W. Zimmerman[1,2], Jude M. Phillip[1,10,11,17,27], Elizabeth M. Jaffee[1,2], Joe W. Gray[9,28], Lisa M. Coussens[28,29], Young Hwan Chang[9,28], Laura M. Heiser[9,28], Genevieve L. Stein-O'Brien[1,2,8,11,30,31*], Elana J. Fertig[1,2,4,5,6,10,32,33*], Paul Macklin[7,*,**]

[1] Department of Oncology, Sidney Kimmel Comprehensive Cancer Center, Johns Hopkins University. Baltimore, MD USA.
[2] Convergence Institute, Johns Hopkins University. Baltimore, MD USA.
[3] Department of Pharmacology, Physiology, and Drug Development, University of Maryland School of Medicine. Baltimore, MD USA.
[4] Institute for Genome Sciences, University of Maryland School of Medicine, Baltimore, MD USA.
[5] Marlene & Stuart Greenbaum Comprehensive Cancer Center, University of Maryland School of Medicine. Baltimore, MD USA.
[6] University of Maryland Institute of Health Computing, University of Maryland School of Medicine. Baltimore, MD USA.
[7] Department of Intelligent Systems Engineering, Indiana University. Bloomington, IN USA.
[8] Department of Neuroscience, Johns Hopkins University. Baltimore, MD USA.
[9] Department of Biomedical Engineering, Oregon Health & Science University. Portland, OR USA.
[10] Department of Biomedical Engineering, Johns Hopkins University. Baltimore, MD USA.
[11] Institute for NanoBioTechnology, Johns Hopkins University, Baltimore, MD 21218, USA.
[12] Department of Genetic Medicine, Johns Hopkins University. Baltimore, MD USA.
[13] Department of Electrical and Computer Engineering, Johns Hopkins University. Baltimore, MD USA.
[14] Data Science and AI Institute, Johns Hopkins University, Baltimore, MD USA.
[15] Department of Pathology, Johns Hopkins University. Baltimore, MD USA.
[16] Department of Cell Biology, Johns Hopkins University School of Medicine. Baltimore, MD, USA.
[17] Department of Chemical and Biomolecular Engineering, Johns Hopkins University. Baltimore, MD USA.
[18] Memorial Sloan Kettering Cancer Center. New York, NY USA.
[19] Department of Informatics, Indiana University. Bloomington, IN USA.
[20] Department of Biomedical Engineering, Georgia Institute of Technology, Emory University. Atlanta, GA USA.
[21] Centre de Recherches en Cancerologie de Toulouse. Toulouse, France.
[22] Department of Medicine, Washington University Saint Louis. St. Louis, MO USA.
[23] Giovannis Institute for Translational Cell Biology, Johns Hopkins University School of Medicine. Baltimore, MD, USA.
[24] Department of Materials Science and Engineering, Johns Hopkins University. Baltimore, MD USA.
[25] Mays Cancer Center, University of Texas Health. San Antonio, TX USA.
[26] MD Anderson Cancer Center. San Antonio, TX USA.
[27] Translational Tissue Engineering Center, Johns Hopkins University School of Medicine, Baltimore, MD, USA.
[28] Knight Cancer Institute, Oregon Health & Science University, Portland, OR USA.
[29] Department of Cell, Developmental and Cancer Biology, Oregon Health & Science University. Portland, OR USA.
[30] Department of Neurology, Johns Hopkins University School of Medicine. Baltimore, MD, USA;
[31] Kavli Neuroscience Discovery Institute, Johns Hopkins University. Baltimore, MD, USA;
[32] Department of Applied Mathematics and Statistics, Johns Hopkins University. Baltimore, MD, USA.
[33] Department of Medicine, University of Maryland School of Medicine. Baltimore, MD USA.

[+]Contributed equally
[*]Corresponding authors: gsteinobrien@jmhi.edu, EJFertig@som.umaryland.edu , macklinp@iu.edu
[**]Lead Contact

FURTHER DETAILS ON THE GRAMMAR

Cell behaviors, reference models, and reference parameter values

To build this grammar, we require clear abstractions of key cell behaviors that frequently occur in multicellular observations and corresponding reference models. In this context, a cell *behavior* is a cell-scale process, such as cycling, death, or phagocytosis. Generally, each behavior can be represented by a small number of continuous phenotypic parameters, describing the rate, magnitude, or frequency of the

behavior. In earlier work, Sluka et al. developed the Cell Behavior Ontology (CBO)[1] as a controlled vocabulary of individual cell behaviors. More recently, we worked with a multidisciplinary coalition to extend and structure behaviors from the CBO and other sources into MultiCellDS[2] (multicellular data standard). In particular, this work defined a ***behavioral cell phenotype*** that collects biophysical characterizations of a cell's behavior, organized hierarchically by function: cycling, death, volume, mechanics, secretion (including uptake), and motility. Since releasing MultiCellDS as a preprint, we have tested this approach to cell behavior through a variety of agent-based simulation and modeling projects[3-17]. Based upon recent immunologic modeling work[4,5,8,9,14,16], we extended phenotype to include cell-cell interactions (phagocytosis, effector attack, and fusion), as well as transformations between cell types (e.g., differentiation, transdifferentiation, and other persistent state changes that persist even when exogenous signals are removed).

Here, we fully describe the grammar's supported cell behaviors, as well as their ***reference implementation*** in PhysiCell and corresponding biophysical parameters.

Cycling

As introduced in prior work[2,13,18], a cell cycle model is a sequence of phases (with indices $i = 0,1,\ldots,n$) and transition rates (or exit rates) $\{r_i\}$ connecting phase i to the next phase i+1. By convention, exit from the first phase (with index 0) is called ***cycle entry***, and cells divide upon exiting last phase (with index n) to return to the 0th phase. In the reference implementation, cell transitions between phases are probabilistic, based upon the transition rate; if a cell is in phase i at time t, then the probability of advancing to phase $i+1$ between the current time t and a future time $t + \Delta t$ is given by

$$P(\text{exit phase } i) = r_i \Delta t. \tag{1}$$

Note that the mean time spent in the ith phase is $1/r_i$.

For example, a four-phase model ("flow cytometry separated") consists of phases G_0/G_1, S, G_2, and M, cycle entry is a transition from G_0/G_1 (Phase 0) to S (Phase 1), and division occurs at the end of M (Phase 3). In the reference implementation[13], at division daughter cells are assigned half the volume of the parent cell and positioned randomly such that (1) they both fit within the parent cell's volume, and (2) the daughter cells have the same center of volume as their parent cell. In the current grammar, the dictionary of cell cycling behaviors and their corresponding parameters are presented below (***Cell cycle and asymmetric division terms***). Future versions of the grammar may include finer grained control on placement of daughter cells after division.

dictionary symbol	synonyms	controllable phenotype parameter
exit from cycle phase 0	cycle entry	r_0
exit from cycle phase 1		r_1
exit from cycle phase 2		r_2
exit from cycle phase 3		r_3
exit from cycle phase 4		r_4
exit from cycle phase 5		r_5
asymmetric division to X		$p_{\mathrm{AD},i,j}$

Cell cycle and asymmetric division terms. Cell cycle and asymmetric division terms in the behavior dictionary, along with corresponding controllable parameters in the PhysiCell reference implementation. Reference values are given in the discussion below, because they depend in part upon how the cell cycle is represented.

Supported cell cycle models and reference parameter values

PhysiCell supports a variety of built-in cycle models, based upon and extending community-defined work[2]. In the PhysiCell method paper[13], we tuned each of these models to give consistent cell division rates (and matching sub-population models, such that less detailed models coarse-grained more detailed models) for MCF-10A cells with a 0.0432 hr^{-1} net birth rate (and a dead cell fraction of approximately 2% reported for MCF10A cells[19]), chosen as a representative mammalian epithelial cell line with frequent but moderate division. Below, we give the fitted reference parameter values for each of these supported models; see the supplementary material and provided source code in Ghafarrizadeh et al. (2018)[13] for further detail, analyses, and references.

FLOW CYTOMETRY SEPARATED MODEL

This model is designed for matching to more detailed flow cytometry that can independently count cells in G_0/G_1, S, G_2, and M phases. Cells begin in the G_0/G_1 phase (Phase 0), which has stochastic (random) duration; "cycle entry" controls exit from this variable phase to enter S phase (Phase 1) by changing it exit rate r_0, which relates to its mean duration $T_0 = 1/r_0$. S phase cells progress to G_2 phase (Phase 2), and G_2 cells proceed to M phase (Phase 3). Upon completion of M phase, the cell divides into two identical daughter cells in G_0/G_1 as described above and in Ghaffarizadeh et al. (2018)[13]. In the reference implementation, S, G_2, and M phases are taken to have fixed duration.

The default parameter values for this model are:

T_0 (mean duration of G_0/G_1):	4.98 hours (fitted)	$r_0 = 0.00335$ min^{-1}.
T_1 (duration of S):	8 hours	$r_1 = 0.00208$ min^{-1}.
T_2 (duration of G_2):	4 hours	$r_2 = 0.00417$ min^{-1}.
T_3 (duration of M):	1 hours	$r_3 = 0.0167$ min^{-1}.

Note that the total cell cycle duration $T_0 + T_1 + T_2 + T_3 = 17.98$ hours, which is consistent with a typical 18-24 hour estimate for mammalian cells[20].

FLOW CYTOMETRY MODEL

This model is designed for matching to more typical flow cytometry that can independently count cells in G_0/G_1, S, and G_2/M phases. Cells begin in the G_0/G_1 phase (Phase 0), which has stochastic (random) duration; "cycle entry" controls exit from this variable phase to enter S phase (Phase 1) by changing its exit rate r_0, which relates to its mean duration $T_0 = 1/r_0$. S phase cells progress to G_2/M phase (Phase 2). Upon completion of G_2/M phase, the cell divides into two identical daughter cells in G_0/G_1 as described above and in Ghaffarizadeh et al. (2018)[13]. In the reference implementation, S and G_2/M phases are taken to have fixed duration.

The default parameter values for this model are:

T_0 (mean duration of G_0/G_1):	5.15 hours (fitted)	$r_0 = 0.00324$ min^{-1}.
T_1 (duration of S):	8 hours	$r_1 = 0.00208$ min^{-1}.
T_2 (duration of G_2/M):	5 hours	$r_2 = 0.00333$ min^{-1}.

Note that the total cell cycle duration $T_0 + T_1 + T_2 = 18.15$ hours, which is consistent with a typical 18-24 hour estimate for mammalian cells[20].

KI67 ADVANCED MODEL

As noted in Ghaffarizadeh et al. (2018)[13] and Macklin et al. (2012)[21], this cycle model is designed for fitting to data stained for Ki67 (a frequently used nuclear cell cycle marker), with further adjustments to

account for the fact that Ki67 is positive both in cells *about* to divide and in cells that have *recently* divided, thus requiring additional attention in fitting. Cells begin in the $Ki67^-$ phase (Phase 0), which has stochastic (random) duration; "cycle entry" controls exit from this variable phase to enter $Ki67^+$ pre-mitotic phase (Phase 1) by changing its exit rate r_0, which relates to its mean duration $T_0 = 1/r_0$. Pre-mitotic $Ki67^+$ phase cells progress to divide into two identical daughter cells in the post-mitotic $Ki67^+$ phase (Phase 2), before returning to the $Ki67^-$ phase. In the reference implementation, $Ki67^+$ (pre-mitotic) and $Ki67^+$ (post-mitotic) are taken to have fixed duration.

The default parameter values for this model are:

T_0 (mean duration of $Ki67^-$):	3.62 hours (fitted)	$r_0 = 0.00460$ min^{-1}.
T_1 (duration of $Ki67^+$ (pre-mitotic)):	13 hours	$r_1 = 0.00128$ min^{-1}.
T_2 (duration of $Ki67^+$ (post-mitotic)):	2.5 hours	$r_2 = 0.00667$ min^{-1}.

Note that the total cell cycle duration $T_0 + T_1 + T_2 = 19.12$ hours, which is consistent with a typical 18-24 hour estimate for mammalian cells[20].

KI67 BASIC MODEL

As noted in Ghaffarizadeh et al. (2018)[13] and Macklin et al. (2012)[21], this cycle model is designed for fitting to data stained for Ki67 (a frequently used nuclear cell cycle marker), with further adjustments to account for the fact that Ki67 is positive both in cells *about* to divide and in cells that have *recently* divided, thus requiring additional attention in fitting. However, this cycle model has been slightly simplified to combine pre- and post-mitotic $Ki67^+$ cells. Cells begin in the $Ki67^-$ phase (Phase 0), which has stochastic (random) duration; "cycle entry" controls exit from this variable phase to enter $Ki67^+$ phase (Phase 1) by changing its exit rate r_0, which relates to its mean duration $T_0 = 1/r_0$. $Ki67^+$ phase cells progress to divide into two identical daughter cells in the quiescent $Ki67^-$ phase (Phase 0). In the reference implementation, $Ki67^+$ is taken to have fixed duration.

The default parameter values for this model are:

T_0 (mean duration of $Ki67^-$):	4.59 hours (fitted)	$r_0 = 0.00363$ min^{-1}.
T_1 (duration of $Ki67^+$):	15.5 hours	$r_1 = 0.00108$ min^{-1}.

Note that the total cell cycle duration $T_0 + T_1 = 20.1$ hours, which is consistent with a typical 18-24 hour estimate for mammalian cells[20].

CYCLING QUIESCENT MODEL

This model is identical to the "Ki67 Basic" model, except that $Ki67^-$ cells are referred to as "quiescent", and $Ki67^+$ cells are referred to as "cycling." It uses the same reference parameters as the Ki67 Basic model.

LIVE MODEL

As noted in Ghaffarizadeh et al. (2018)[13], this cycle model is for basic data that merely measure cell populations and fit the overall population division rate. Cells begin in the "live" phase (Phase 0), which has stochastic (random) duration; "cycle entry" controls exit from this variable phase to enter another "live" phase (Phase 0) by changing its exit rate r_0, which relates to its mean duration $T_0 = 1/r_0$. Live cells divide into two identical daughter live cells at the end of this phase. In the reference implementation, no phases are taken to have fixed duration.

The default parameter values for this model are:

T_0 (mean duration of cell cycle):	23.1 hours (fitted)	$r_0 = 0.00072$ min^{-1}.
T_1 (duration of $Ki67^+$):	15.5 hours	$r_1 = 0.00108$ min^{-1}.

Note that the total cell cycle duration T_0 = 23.1 hours, which is consistent with a typical 18-24 hour estimate for mammalian cells[20], and directly fitted to reported values for MCF10A cells as noted in Ghaffarizadeh et al. (2018)[13].

Asymmetric division

We allow for a simple model of asymmetric division in which one, and only one, of the two daughter cells can be of a different cell type than the parent cell. These are represented as a table of probabilities $p_{\mathrm{AD},i,j}$, for one daughter cell from a division of cell type i to be of cell type j immediately after division. The one dictionary symbol is *asymmetric division to X* without synonyms and with parameters given by $p_{\mathrm{AD},i,j}$. When deciding the fate of the candidate daughter cell, if the probabilities sum to less than 1, the remainder of the probability is assigned to symmetric division, i.e., keeping the type of the parent. If they sum to more than 1, then an error is thrown. See also the descriptions above in ***Cell cycle and asymmetric division terms***.

Default parameters

Asymmetric division is off by default, and therefore there are no default parameters for this behavior.

Death

In the initial grammar, we support apoptosis (as a primary form of non-immunogenic cell death) and necrosis (as a key form of immunogenic cell death)[22]. We note that at a cellular and multicellular perspective, death is not merely a discrete event, but rather entry into a cascade of processes[22-27]; as noted in prior modeling and analysis[13,21,22], every model of death has a nonzero, finite duration on the order of hours (for apoptosis) to days or weeks (for necrosis). In the reference implementation[13], live cells have an apoptotic death rate d_A, such that the probability that a cell becomes apoptotic between the current time t and a future time $t+\Delta t$ is given by

$$P(\text{cell becomes apoptotic}) = d_A \Delta t. \tag{2}$$

Once the cell becomes apoptotic, it shrinks until being removed from the simulation.

Similarly, live cells have a necrotic death rate d_N, such that the probability that a cell becomes necrotic between the current time t and a future time $t+\Delta t$ is given by

$$P(\text{cell becomes necrotic}) = d_N \Delta t \tag{3}$$

In the reference necrosis model, cells initially swell until reaching a bursting volume (by default, 200% of the cell's volume at the start of necrosis), and then gradually shrink over the course of days.

Full details on the reference parameter values for apoptosis and necrosis (including rates of cell volume change and durations) are found in prior work[13,21]. In the current grammar, the dictionary of behaviors and their corresponding parameters are presented below (***Death terms***).

dictionary symbol	synonyms	controllable phenotype parameter	reference or recommended values
apoptosis		d_A	0.0000532 min^{-1}
necrosis		d_N	maximum rate of 0.00283 min^{-1}

Death terms. Death terms in the behavior dictionary, along with corresponding controllable parameters in the PhysiCell reference implementation.

Reference parameter values

The durations of apoptosis (degradation and shrinkage over approximately 8.6 hours) and necrosis (approximately 6 hours of oncotic swelling, followed by slower fluid loss, nuclear degradation over days to

weeks) were estimated in depth in earlier work[22] and in Ghaffarizadeh et al. (2018)[13]; see the supplementary information for detailed analyses and further references.

As noted in Macklin et al. (2012)[21] and Hyun and Macklin (2013)[28], these time scale estimates can be combined with the known apoptotic fraction (2% for MCF10A cultures as noted above) to fit an apoptotic death rate *d*A based upon the improved calibration protocol of Hyun and Macklin (2013)[28]. Based upon this calibration fitting, Ghaffarizadeh et al. (2018) obtained a reference apoptotic death rate:

$$d_{\mathrm{A}} = 0.00319\ \mathrm{hr}^{-1} = 0.0000532\ \mathrm{min}^{-1}.$$

For (non-glycolytic) cells under very low oxygen conditions, their survival duration was estimated[13] to be on the order of 6 hours, giving a default (maximum) necrosis rate (when not inhibited by oxygen) of:

$$d_{\mathrm{N}} = 0.17\ \mathrm{hr}^{-1} = 0.00283\ \mathrm{min}^{-1}$$

Secretion, uptake, and generalized chemical export

Cells secrete and uptake (consume) chemical factors that diffuse in the extracellular microenvironment, with a fixed mathematical form first introduced in BioFVM[29] and later incorporated in PhysiCell[13]. For any diffusible substrate ρ, BioFVM solves the reaction-diffusion equation in Equation (4):

$$\frac{\partial \rho}{\partial t} = D\nabla^2\rho - \lambda\rho + \sum_{\text{cells } i}\left(\delta(\boldsymbol{x}-\boldsymbol{x}_i)V_i\left[\overbrace{S_i(\rho_i^*-\rho)}^{\text{secretion}} - \overbrace{U_i\rho}^{\text{uptake}}\right] + \delta(\boldsymbol{x}-\boldsymbol{x}_i)\overbrace{E_i}^{\text{export}}\right) \quad (4)$$

Here, $\boldsymbol{x}_i$ is the ith cell's position, $\delta(\boldsymbol{x})$ is the Dirac delta function that mathematically focuses the cell-based source/sink at its center, V_i is the volume of the ith cell, S_i is its secretion rate of the substrate (with dimensions 1/time), ρ^*_i is the "target value" of its secretion (i.e., secretion slows as ρ approaches this value), and U_i is its uptake or consumption rate (also with dimensions 1/time). When these fixed secretion and uptake forms are insufficient to match experiments or advanced modeling forms, we provide a generic *net export* term E_i (with dimensions substrate/cell/time). Because the microenvironment can have multiple diffusing substrates, the grammar can automatically expand to reference secretion, uptake, and export parameters for each one, therefore defining a family of symbols as summarized below (***Secretion, uptake, and export terms***).

dictionary symbol	synonyms	controllable phenotype parameter	reference or recommended values
X secretion		S_i	0 by default<br>recommend 10 min^{-1} for secreting cells
X secretion target	*X* secretion saturation density	ρ_i^*	1 (for scaled or nomdimensionalized substrates)<br>max value (for non-scaled substrates)
X uptake		U_i	0 by default; for uptaking cells, determine by diffusion length scale in dense or packed tissue
X export		E_i	0 by default; determine by mass balance when needed (e.g., flux balance models)

Secretion, uptake, and export terms. Secretion, uptake, and export terms in the behavior dictionary, along with corresponding controllable parameters in the PhysiCell reference implementation. *X* denotes the name of any diffusible substrate (e.g., oxygen).

Reference parameter values

Parameters governing the transport of chemical factors are estimated based upon known diffusion coefficients and estimates of diffusion length scales. For example, we previously[21,29] considered the oxygen diffusion length scale $L = \sqrt{D/U}$ in densely cell-packed tissues to be approximately 100 µm and the diffusion coefficient $D = 10^5$ µm^2/min, thus giving an cellular oxygen uptake rate $U = 10$ min^{-1} to match the

expected diffusion length scale. When needed, we use decay rates on the order of 1% or less of the cell uptake rate.

When modeling secreted diffusible substrates, we choose a secretion rate strong enough such that the non-concentration is near the maximum measured value (1 for scaled or non-dimensionalized substrates). In day-to-day use, setting $S = 10\ \mathrm{min}^{-1}$ tends to meet this criterion. For non-secreted substrates, the reference value is 0. Similarly, the target density ρ^* is set the maximum concentration (1 for scaled or non-dimensionalized substrates).

We typically do not use the export parameter E and so the reference value of these is 0, but they can be set based upon direct measurements (e.g., in metabolic models[30,31]).

(Biased) migration and chemotaxis

As previously introduced in PhysiCell[13], cell migration (or motility) is represented as a biased random walk: the cell's migration direction $\boldsymbol{d}_{\mathrm{mot}}$ is a combination of a random (unit vector) component $\boldsymbol{\xi}$ and a non-random directed component (a migration bias direction) $\boldsymbol{d}_{\mathrm{bias}}$, which is normalized to be a unit vector as in Equation (5):

$$\boldsymbol{d}_{\mathrm{mot}} = \frac{b\,\boldsymbol{d}_{\mathrm{bias}} + (1-b)\boldsymbol{\xi}}{\|b\,\boldsymbol{d}_{\mathrm{bias}} + (1-b)\boldsymbol{\xi}\|} \tag{5}$$

Here, $0 \le b \le 1$ is the cell's *migration bias*: if $b = 1$, then migration occurs completely along the bias direction $\boldsymbol{d}_{\mathrm{bias}}$, while $b = 0$ corresponds to purely Brownian motion. We then obtain the *migration velocity* $\boldsymbol{v}_{\mathrm{mot}}$ by multiplying the direction by the *migration speed* (s):

$$\boldsymbol{v}_{\mathrm{mot}} = s\,\boldsymbol{d}_{\mathrm{mot}}. \tag{6}$$

In our formulation, the cell's migration speed (and hence migration velocity) can change dynamically based upon any further modeling rules, even if the migration direction does not. Cell migration has a *persistence time* T_{persist}: between the current time t and a future time $t + \Delta t$, the probability of choosing a new migration direction (by re-evaluating Equation (6)) is given by

$$P(\text{change migration direction}) = \frac{\Delta t}{T_{\mathrm{persist}}}. \tag{7}$$

Note that this gives a mean time of T_{persist} between direction changes.

Chemotaxis is modeled by setting the migration bias direction along the gradient of one or more chemical substrates in the microenvironment. If available chemical substrates are $c_0, c_1, \ldots, c_m$, then we set

$$\boldsymbol{d}_{\mathrm{bias}} = \omega_0 \nabla c_0 + \cdots + \omega_m \nabla c_m, \tag{8}$$

where ω_i is a weighting (*chemotactic response*) for each chemical gradient: $\omega_i < 0$ for migration against the gradient (along $-\nabla c_i$), $\omega_i > 0$ for migration along the gradient, and $\omega_i = 0$ if the gradient makes no contribution. Because the microenvironment can have multiple diffusing substrates, the grammar can automatically expand to a family of chemotactic response terms. See ***Migration terms*** below.

dictionary symbol	synonyms	controllable phenotype parameter	reference or recommended values
migration speed		s	1 μm/min
migration bias		b	0.5
migration persistence time		T_{persist}	5 min
chemotactic response to *X*	chemotactic sensitivity to *X*	ω_i	0

Migration terms. Migration terms in the behavior dictionary, along with corresponding controllable parameters in the PhysiCell reference implementation. *X* denotes the name of any diffusible substrate (e.g., oxygen).

Reference parameter values

To estimate parameters for migration and chemotaxis, experimental data or literature values are essential. Migration bias b can be derived from cell trajectories by comparing displacement in the bias direction versus total displacement[32]. Values typically range from 0.5 to 1.0 for strong directional cues, and approach 0 for random motion. The reference value is $b = 0.5$ for moderately directed migration (e.g., along chemotactic cues). In future work, this can be further correlated with directionality (displacement divided by total distance traveled) and theoretical estimates for Brownian walks[33].

The migration speed s can be directly measured by cell-tracking experiments (ideal), or estimated from average cell displacement over time in controlled experiments. For migratory mammalian epithelial cells (e.g., cancer cells[34-36]: 0.2-2 micron/min) with reported migration speeds of on the order of 0.1 to 1 μm/min, we use $s = 1$ μm/min as the reference value. More specialized migratory mammalian cells (e.g., fibroblasts[37,38]: 0.2 to 1.5 μm/min; macrophages[39]: 1-10 μm/min; and T cells[35,39-41]: 7-30 μm/min) have reported speeds on the order of 0.1-10 μm/min, we also use s =1 μm/min as the default value. Future specific "digital cell lines"[2,42] for leukocytes may want to use a higher default speed of 10 μm/min.

The persistence time T_{persist}, reflecting intervals between directional changes, is estimated from cell trajectories by analyzing time between significant directional shifts, or direct observation of cell tracks. The literature reports variations in cell migration direction (i.e., persistence times) on the order of 2-15 minutes (fibroblasts[37,38]: 5-15 min, T-cells[5]: 2-3 min; several cell lines[43] migrating in 3D at 1-10 μm/min: 1-10 min). Thus, we use a default persistence time of 5 min.

Chemotactic response coefficients ω_i are estimated using assays like microfluidics, with positive values indicating chemoattraction and negative for chemorepulsion. When modeling chemotaxis along a single gradient, set its corresponding sensitivity to 1 (-1 for chemorepulsion) and all other sensitivities to 0. By default, all chemotactic sensitivities are set to 0.

Cell-cell adhesion

Two models of cell-cell adhesion are supported: a looser cell-cell adhesion using potential functions (as in prior mathematical models[44-49]), and more persistent elastic springs[6,50,51]. In the reference PhysiCell implementation for potential-based adhesions, if cells i and j are adhered and within interaction distance, then the contribution to the velocity of cell i is given by

$$\sqrt{A_{ij}\,\alpha_i \cdot A_{ji}\alpha_j}\left(1-\frac{\|\boldsymbol{x}_j-\boldsymbol{x}_i\|}{R_{A,i}R_i+R_{A,j}R_j}\right)^{n+1}\frac{(\boldsymbol{x}_j-\boldsymbol{x}_i)}{\|\boldsymbol{x}_j-\boldsymbol{x}_i\|}, \tag{9}$$

Where α_i is cell i's adhesive strength, A_{ij} is the (relative) adhesive affinity of cell i to cell j (which for example could be modeled based upon adhesive receptor expressions of cells i and j), $\boldsymbol{x}_i$ is the position of cell i, and $R_{A,i}$ is cell i's relative maximum adhesion distance (the largest distance it can extend for adhesion, as a multiple of its radius), R_i is the cell's radius, and n is an integer power (typically 1) chosen for the smoothness of the force's behavior as $r \to R_A$ [13]. Notice that this adhesion only depends upon the

relative distance between cells, and not their prior history. If the cells are identical, the coefficient reduces to $A_{ii}\alpha_i$. See ***Cell-cell adhesion and repulsion terms*** below.

To model cell adhesions that form more readily than they break, we also support an elastic cell-cell adhesion model. Cell i forms an elastic adhesion to cell j between the current time t and a future time $t + \Delta t$ at an attachment rate $r_{Ai,j}$ (and thus probability $r_{A,i,j}\Delta t$), provided that:

- The distance $\|\boldsymbol{x}_j - \boldsymbol{x}_i\|$ between the cells does not exceed the maximum adhesion interaction distance, and
- Neither cell i nor cell j has exceeded their individual maximum number of cell adhesions ($n_{M,i}$ and $n_{M,j}$).

We calculate this cell-cell attachment rate $r_{A,i,j}$ as

$$r_{A,ij} = r_{A,i}\, A_{ij}, \tag{10}$$

where $r_{A,i}$ is cell i's rate of forming elastic attachments and A_{ij} is the adhesive affinity of cell i to cell j. (Notice that cell i can form attachments to cell j, and cell j can form attachments to cell i independently with independent rates.) Analogously, cell i can detach from cell j at rate $u_{D,i}$, so that during any time interval t to $t + \Delta t$, the cell i can detach from cell j with probability $u_{D,i}\Delta t$. When the cells are elastically adhered, the adhesion contributes to cell i velocity via:

$$\sqrt{A_{ij}\, \epsilon_i \cdot A_{ji}\, \epsilon_j} \cdot \left(\boldsymbol{x}_j - \boldsymbol{x}_i\right), \tag{11}$$

where ϵ_i is cell i's elastic constant. Notice that if cells i and j are identical, then the coefficient simplifies to $A_{ii}\epsilon_i$. The full parameter list for basic and elastic cell-cell adhesion (and cell repulsion) is found below (***Cell-cell adhesion and repulsion terms***).

dictionary symbol	synonyms	controllable phenotype parameter	reference or recommended values
cell-cell adhesion		α_i	0.4 (μm/min)
cell-cell adhesion elastic constant		ϵ_i	0.01 min^{-1}
adhesive affinity to *X*	adhesive affinity to cell type *N*	A_{ij}	1
relative maximum adhesion distance		$\frac{R_{A,i}}{R_i}$	1.25 (often increased to 1.5)
cell attachment rate		$r_{A,i}$	0 (recommend 1 min^{-1} when enabled)
cell detachment rate		$u_{D,i}$	0 (recommend 1 min^{-1} when enabled)
maximum number of cell attachments		$n_{M,i}$	12 (for 3D); 6 (for 2D)
cell-cell repulsion		β_i	10 (μm/min)
movable	is_movable; is movable	m	true

Cell-cell adhesion and repulsion terms. Cell-cell adhesion and repulsion terms in the behavior dictionary, along with corresponding controllable parameters in the PhysiCell reference implementation. Here, *X* denotes any cell type, and *N* denotes its integer type or index within the simulation.

Reference parameter values

PhysiCell defaults to "classic" adhesion based upon adhesive and "repulsive" potentials[13,21] (see also the next section), with elastic spring-based adhesion set off (i.e., the attachment rates $r_{A,i}$ and detachment rates $u_{D,i}$ are 0). After mathematical analyses, cell-cell interaction mechanics were cast in an "inertialess"

formulation where forces quickly come to equilibrium to give a quasi-steady velocity; see Macklin et al. (2012)[21], Ghaffarizadeh et al. (2018)[13], and references therein. Based upon analyses of prior experiments on cell mechanical relaxation[52,53], and balancing the relaxation force with cell-cell adhesion for cells in a mostly (90%) confluent tissue with a maximum interaction distance of 1.25 cell radii[54,55] as in Hyun and Macklin (2019)[28], we set default parameters for cell-cell adhesion at α = 0.4 µm/min 0.4, and cell-cell repulsion at β = 10 µm/min. By default, all cells (with nonzero adhesion) are assumed to have equal affinity so the default is A_{ij} =1 for all i and j.

To estimate recommended values for the elastic adhesion models, we note that prior work used an elastic constant of 0.01 min^{-1} to maintain comparable equilibrium cell-cell spacings as the potential-based adhesion, and to also maintain strong adhesions for migrating cells. In epithelial tissues, the (cadherin) cell adhesion turnover is on the order of minutes to tens of minutes[56], so we recommend setting the attachment rates $r_{A,i}$ and detachment rates $u_{D,i}$ to 1 min^{-1}. For 3-D simulations, we recommend setting the maximum number of attachments (n_M) to 12 for a packed tissue; set this value to 6 (or less) in 2D. When using spring-based adhesion, we recommend setting the potential-based adhesion parameter (α) to zero to avoid "double-counting" adhesion.

Resistance to deformation and movement

PhysiCell[13], as many agent-based simulation frameworks[44-48], uses cell "repulsion" to model resistance to deformation, compression, and cell overlap. In the reference PhysiCell implementation for potential-based repulsion, if cells i and j are in contact, then the contribution to the velocity of cell i is given by

$$-\sqrt{\beta_i \cdot \beta_j}\left(1-\frac{\|\boldsymbol{x}_j-\boldsymbol{x}_i\|}{R_i+R_j}\right)^{n+1}\frac{(\boldsymbol{x}_j-\boldsymbol{x}_i)}{\|\boldsymbol{x}_j-\boldsymbol{x}_i\|}, \tag{12}$$

where R_i is cell i's radius and β_i is its cell-cell repulsion coefficient as defined in prior work[13,21,28]. In cases where a cell should be regarded as a rigid object or obstacle (e.g., if rigidly adhered to an underlying matrix), it can be flagged as *unmovable* using a "movable" parameter m. If m is false ($m = 0$), then the cell can impart adhesive and repulsive forces on other cells, but they cannot impose reciprocal forces that contribute to the cell's movement. The full parameter list for cell-cell adhesion and repulsion is found above in ***Cell-cell adhesion and repulsion terms***.

Reference parameter values

See the discussion in the prior section on cell-cell adhesion.

Cell Transition or Transformation (changing type)

Many biological systems require cell type transformations from one type to another, particularly via differentiation, transdifferentiation, and mutation. Mathematically, these all can be represented as a transition or transformation (type change) rate $r_{T,ij}$ from type i to type j, with the probability of a type change between the current time t and a future time $t + \Delta t$ given by

$$r_{T,ij}\,\Delta t. \tag{13}$$

In the simplest reference implementation, when a cell transforms, it retains its state variables (volume, position, internalized substrates, and conserved custom variables), and it overwrites all other phenotype parameters from the new cell type. These parameters are summarized below (***Key cell-cell interaction terms***).

Reference parameter values

The transformation or transition between phenotypes is determined by the specifics of each developed model, and by default, no transitions occur between phenotypes; all these parameters are set to 0 by default.

Fusion

As a simple reference model of cell fusion, if cell i is in contact with cell j, the cells can fuse between the current time t and a future time $t + \Delta t$ with probability

$$r_{F,ij}\ \Delta t, \tag{14}$$

where $r_{F,ij}$ is the fusion rate for the cell i to the type of cell j. In our reference model, when cells i and j fuse, the newly fused cell:

- is placed at the center of volume of the pre-fused cells i and j
- combines the volumes of the cells i and j
- combines the number of nuclei in cells i and j (if tracked)
- combines all internalized substrates in cells i and j (if tracked)

At the present time, there is no community consensus on what *phenotype parameters* should be acquired by the newly fused cell; future versions of the reference model may take a (volume-weighted) average of the pre-fused cells' phenotypes. Moreover, community consensus is required to determine if the fused cell should shrink towards a typical single cell size or retain its larger size in the long term. Behavioral parameters that can be modulated by our grammar are summarized below (***Key cell-cell interaction terms***).

dictionary symbol	synonyms	controllable phenotype parameter	reference or recommended values
transition to *X*	transition to cell type *N*<br>transform to *X*<br>transform to cell type *N*	$r_{T,ij}$	0
fuse to *X*	fuse to cell type *N*	$r_{F,ij}$	0 (recommend 0.03 min^{-1} for epithelial fusion)
phagocytose apoptotic cell	phagocytosis of apoptotic cell<br>phagocytosis of apoptotic cells	$r_{PA,i}$	0.1 min^{-1}
phagocytose necrotic cell	phagocytosis of necrotic cell<br>phagocytosis of necrotic cells	$r_{PN,i}$	0.017 min^{-1}
phagocytose other dead cell	phagocytosis of other dead cell<br>phagocytosis of other dead cells	$r_{PO,i}$	0.1 min^{-1}
phagocytose *X*	phagocytose cell type *N*<br>phagocytosis of *X*	$r_{PL,ij}$	0
attack *X*	attack cell type *N*	$r_{A,ij}$	0 (recommend 0.1 min^{-1} for cytotoxic T cells)
immunogenicity to *X*	immunogenicity to cell type *N*	$g_{A,ij}$	1
attack damage rate		r_{AD}	1 min^{-1}
attack duration		T_{attack}	0 (recommend 15 min for cytotoxic T cells)
damage rate		r_{damage}	0 (recommend 1 min^{-1} when modeling damage)
damage repair rate		r_{repair}	0 (recommend 0.012 min^{-1} for recovery from sublethal T cell attack)

Key cell-cell interaction terms. Key cell-cell interaction terms (focused on transformation, fusion, phagocytosis, and effector attack) and cell integrity in the behavior dictionary, along with corresponding controllable parameters in the PhysiCell reference implementation. Here, *X* denotes any cell type, and *N* is the integer index of any cell type in the simulation.

Reference parameter values

As noted in the previous paragraph, default assumption is no fusion, and no reference values are provided; all fusion rates are set to 0 by default. In problems requiring epithelial cell fusion, we would recommend a fusion rate on the order of 0.033 min^{-1}, equivalent to the observed time scale of 30 minutes to complete cell fusion[57,58].

Phagocytosis (or predation or ingestion)

We include a simple reference model of phagocytosis (or predation or ingestion), based on recent modeling[4,5,8]: if cell *i* is in contact with (live) cell *j*, then between the current time *t* and a future time $t + \Delta t$, cell *i* has a probability of phagocytosing cell *j* given by:

$$r_{PL,ij}\,\Delta t, \tag{15}$$

where $r_{PL,ij}$ is cell i's rate of phagocytosing live cells of type j. If cell j is dead, then the phagocytosis rate depends on the type of death: apoptotic, necrotic, or a user-defined death model. It does not depend on the cell type of j. The probabilities of phagocytosing such a cell in that time interval are $r_{PA,i}\Delta t$, $r_{PN,i}\Delta t$, and $r_{PO,i}\Delta t$, respectively.

In the reference model, when a cell phagocytoses (or ingests) another, it absorbs from the target cell its volume, a fraction of its internalized substrates, and any conserved custom data. It retains its original position. (In the event that a more detailed volume is represented as in prior work[13], cell j's solid volume is added to cell i's cytoplasmic solid volume, and cell j's fluid volume is added to cell i's overall fluid volume.) If the simulation framework actively regulates the volume of cell *i* (e.g., to maintain a target volume[13]), then over time cell *i* will return to its previous volume, as a simplified model of degradation of the phagocytosed cell materials. These parameters are summarized above in ***Key cell-cell interaction terms***.

Reference parameter values

Recent experiments[59] that tracked phagocytic cells found that macrophages require more time to phagocytose necrotic cells than apoptotic cells, requiring on the order of 5-10 minutes of contact time to phagocytose an apoptotic cell, and on the order of 60 minutes to phagocytose a necrotic cell; therefore, for phagocytic cells we set a default phagocytosis rate for apoptotic cells to r_{PA}=0.1 min^{-1}, and a rate of necrotic cell phagocytosis to r_{PN}=0.017 min^{-1}. For other dead cells, we recommend the apoptotic rate of r_{PO}=0.1 min^{-1}. See ***Key cell-cell interaction terms***.

By default we set the phagocytosis rate of live cells to 0; those modeling live cell phagocytosis are recommended to analyze the literature on amoeboid cell kill (e.g., in this reference[60]). Interestingly, amoebas can demonstrate "trogocytosis" (taking small bites of a live cell, rather than whole-cell ingestion) [60], which may be modeled instead as an "attack"; indeed, experiments observe that approximately 30 minutes of attack can sufficiently damage a cell's membrane integrity to cause death. (See ***Effector attack*** below).

(Effector) attack

Based on recent models[4,5,8,61,62], we define a reference model of cytotoxic effector attack (e.g., CD8 T inflicting fatal damage on a target cell via perforin and granzymes[63-65]). If cell *i* is in contact with cell *j*, then its probability of initiating an attack on cell *j* between time *t* and a future time $t + \Delta t$ is given by

$$P(\text{start attack}) = r_{A,ij} \cdot g_{ji}\,\Delta t, \tag{16}$$

where $r_{A,ij}$ is cell i's rate of attacking cells of type j, and g_{ji} is cell j's immunogenicity to cell *i*. When cell *i* attacks cell j, an elastic adhesion between the cells forms keeping them in close contact[66,67]. Cell *i* begins

increasing the (dimensionless) damage on cell j, d_j, at a rate r_{AD} (taken to be 1 min^{-1} in the reference implementation). Throughout the attack, therefore, d_j increases by $r_{\mathrm{AD}}\,\Delta t$ in every Δt time step. Concurrently, the total attack time logged by cell j is increased by Δt. Cell i also increments its total damage delivered by $r_{AD}\,\Delta t$ to track this quantity. The length of the attack is governed by the attack duration parameter, T_{attack}. The probability of an attack ending between time t and $t + \Delta t$ is given by

$$P(\text{end attack}) = \frac{\Delta t}{T_{\text{attack}}}, \tag{17}$$

Note that if multiple cells are attacking cell j simultaneously, then both the damage and total attack time increase more rapidly[68,69], which can lead to more rapid death in the target cell j. We note that in this reference model, effector attack increases damage in the target cell j, but it does not cause death without additional hypotheses relating damage to a death rate. Behavioral parameters that can be modulated by our grammar are summarized above (***Key cell-cell interaction terms***). We also note that this formulation could be for any generalized form of (non-phagocytic) cell attack.

Reference Parameter Values (including recommended values for damage response rules in target cells)

To estimate effector attack reference values, we analyzed detailed cell tracking experiments by Weigelin et al.[69], who observed that from the start of a single cytotoxic cell's attack on a tumor cell, an average 1.8 hours (108 min) of contact time are required to initiate cell death. They also reported that on average, three attacks are required to initiate death. When combined with the mean time required to start death (for a given apoptosis rate), and accounting for the lag time between the three attacks, this implies a total time to death T_{death}:

$$108 \text{ min} = T_{\text{death}} = T_{\text{attack1}} + T_{\text{contact2}} + T_{\text{attack2}} + T_{\text{contact3}} + T_{\text{attack3}} + T_{\text{apop}}.$$

Here, T_{contact} is the mean time a cytotoxic cell spends in contact with target cell before initiating an attack (on the order of 6 to 16 minutes[69], and so we set this value at 10 minutes), T_{attack} is the mean duration of an attack (95% of CTL-target contacts were observed to have a median duration of 15 minutes[69]), and $T_{\mathrm{apop}} = 1/r_{\mathrm{A,max}}$ is the expected time to death for a cell after sustaining 3 attacks. Substituting these values, we can solve for T_{apop}:

$$108 \text{ min} = 15 \text{ min} + 10 \text{ min} + 15 \text{ min} + 10 \text{ min} + 15 \text{ min} + T_{\text{apop}} \Rightarrow T_{\text{apop}} = 43 \text{ min}.$$

Thus, the recommended maximum apoptosis rate is $r_{\mathrm{A,max}} = 0.023\ \mathrm{min}^{-1}$, after reaching approximately 45 minutes of attack (3 total attacks).

Because "damage" is a dimensionless quantity, we recommend an attack damage rate of 1 min^{-1}, so that the damage variable tracks the total attack time that can most directly be matched to experiments such as those by Weigelin et al.[69]. In our recommended apoptotic response model (for target cells), we use a relatively large Hill power of ~10 to give a "switch-like" response after reaching 45 minutes of attack. Thus, the recommended rule is (noting that we use min for time units):

damage increases apoptosis towards 0.023 with Hill power 10 and half max 45

These main parameters for cytotoxic cells are summarized in ***Key cell-cell interaction terms*** above.

Cell Integrity

We introduced a basic "cell integrity" structure to the cell phenotype, used for modeling general damaged caused to a cell by a variety of mechanisms, such as cytotoxic drugs, toxins, and effector attack. In that general model, we simulate the *additional* (or independent) damage from non-effector attack, and a simple exponential repair model (e.g., as has been observed for ionizing DNA damage[70-72]):

$$\frac{d[\text{damage}]}{dt} = r_{\text{damage}} + (\text{separate damage from attacking cells}) + r_{\text{repair}}[\text{damage}]$$

We generally recommend only modeling one damage type at a time (in a given cell), e.g., effector attack *or* drug damage. Future versions of the modeling grammar will extend to more finely detailed types of attack (e.g., membrane damage, DNA damage, oxidative damage) as driven by community needs and interest.

Reference Parameter Values

By default, $r_{\text{damage}} = 0$ and $r_{\text{repair}} = 0$. As in cytotoxic attacks, we recommend setting $r_{\text{damage}} = 1\ \text{min}^{-1}$ for cells undergoing damage, so that the damage variable can track total accumulated damage time (e.g., number of minutes of exposure to cytotoxic drug at a specific concentration) for direct experimental matching.

Similarly, the choice of r_{repair} should be based upon the type of damage being modeled. For example, in cytotoxic attack, Weigelin et al.[69] observed cell lethality when three attacks were delivered with less than 50 minutes between attacks, with a median recovery time of 250 minutes after a sublethal hit. Assuming that the cells repair 95% of their damage after 250 minutes, this yields $r_{\text{repair}} = 0.012\ \text{min}^{-1}$. For cytotoxic drugs or radiation therapies (particularly those that damage DNA), we would recommend an analysis of DNA repair rates. See the summary above (***Key cell-cell interaction terms***).

Other terms

Because scientists may require custom biology not yet supported in our grammar or reference implementation, we allow a *custom* symbol to access any custom cell parameters. We also reserve symbols for cell-basement mechanical interactions that currently lack reference implementations. See ***Other terms*** below.

dictionary symbol	synonyms	controllable phenotype parameter
custom:*X*	custom: *X*, custom *N*	*X*
cell-BM adhesion	cell-membrane repulsion	None (symbol reserved)
cell-BM repulsion		None (symbol reserved)

Other terms. Other cell terms in the behavior dictionary, along with corresponding controllable parameters in the PhysiCell reference implementation. Here, *X* is a custom cell variable (parameter), and *N* is its integer index.

Signals

Signals are (typically exogeneous but sometimes internal) stimuli or information that can be interpreted by a cell to drive behavioral or state changes. In the context of mathematical modeling, signals are inputs to constitutive laws or agent rules. We broadly surveyed mathematical and biological models from cancer biology[73-84], tissue morphogenesis[42,75,85-88], immunology[4,5,8,73,89,90], and microbial ecosystems[91,92], to generalize classes of inputs to cell behavioral rules, generally including chemical factors, mechanical cues, cell volume (e.g., for volume-based cycle checkpoints), physical contact with cells, live/dead status, current simulation time (for use in triggering events), and accumulated damage (e.g., from effector attack[63-65]). We thus define the following forms of signals below (***List of signal terms***).

Some signals were included at the request of the mathematical modeling community; for instance, the *time* signal can trigger global model events (e.g., exposure to a dose of radiation therapy), and *apoptotic* and *necrotic* allow more fine-grained control of dead cells. The *damage* signal is currently a flexible, generic term that could describe membrane, nuclear, mitochondrial, DNA, or other types of damage as needed by the specific context of the modeling application. The *total attack time* signal similarly allows "area-under-the-curve" (AUC) models that require total exposure time to attacking cells. The *custom*

family of signals enables greater extensibility by allowing direct access to model-specific cell variables, without the need to formally extend the language and its vocabulary itself.

Over time, new signals can readily be added to the standard vocabulary based on community feedback, or when new signals emerge as widespread in modeling. For example, more specific types of damage (e.g., DNA damage or mitochondrial damage) may appear widely in some biological domains, thus justifying the addition of multiple, more specific damage signals in the vocabulary.

We note that while some signals can be found in existing ontologies (e.g., many diffusible chemical substrates in ChEBI[93,94], mechanical pressure in the ontology for physics in biology (OPB)[95] or non-specific pressure in PATO[96], or non-specific cell-cell contact in the cell behavior ontology (CBO)[1], no single ontology was found to incorporate our broader family of signals in a form that can be concretely and algorithmically generated for a specific simulation or mathematical model system.

**dictionary symbol**	**synonyms**	**accessible variable (and notes)**
X		concentration of extracellular diffusible substrate *X* at cell location
intracellular *X*	internalized *X*	total internalized substrate *X* in the cell
X gradient	grad(*X*), gradient of *X*	norm of the gradient of extracellular diffusible substrate *X*
volume		total cell volume
pressure		(nondimensionalized) mechanical pressure acting upon the cell
contact with *Y*	contact with cell type *N*	number of live cells of type *Y* in physical contact with the cell
contact with live cell	contact with live cells	number of live cells in physical contact with the cell
contact with apoptotic cell	contact with apoptotic cells	number of apoptotic cells in physical contact with the cell
contact with necrotic cell	contact with necrotic cells	number of necrotic cells in physical contact with the cell
contact with other dead cell	contact with other dead cells	number of other dead (neither apoptotic nor necrotic) cells in physical contact with the cell
contact with dead cell	contact with dead cells	number of dead cells in physical contact with the cell
contact with basement membrane	contact with BM	1 if in contact with a basement membrane, and 0 otherwise (reserved symbol for future reference models)
attacking	is attacking	1 if the cell is attacking another cell, and 0 otherwise (see ***effector attack*** above)
damage		total cell damage (see ***effector attack*** above)
total attack time		total accumulated attack time on the target cell by effector cells (see ***effector attack*** above)
damage delivered	total damage delivered	total damage dealt by this (attacking) cell via effector attack (see ***effector attack*** above)
dead	is dead	1 if the cell is dead, and 0 otherwise
apoptotic	is_apoptotic	1 if the cell is apoptotic, and 0 otherwise (or if the cell is undergoing a non-necrotic cell death)
necrotic	is_necrotic	1 if the cell is necrotic, and 0 otherwise
time	current time, global time	current elapsed simulation time
custom:*Z*	custom: *Z*, custom *M*	access to a cell's custom parameter *Z*

List of signal terms. A list of symbols in our vocabulary of signals that can be used to drive behavioral or state changes in cells. Here, *X* is any diffusible substrate, *Y* is a cell type (with integer index *N*), and *Z* is a customized cell parameter (or variable) with integer index *M*.

Full description of the modeling grammar

Now that we have introduced the key signals and behaviors, we now describe our grammar to modulate cell behaviors in response to signals. Cell behavioral hypotheses are written as human-readable statements of the form:

In [cell type T]:

[signal S] [increases / decreases] [behavior B] [optional parameters 1 & 2]. [optional statements].

Here, [signal S] and [behavior B] are symbols defined in our “dictionaries” above. This construction allows us to efficiently “bundle” multiple behavioral response statements for a cell type.

Optional parameters 1: base and maximum effect

These parameters are used to specify the maximum effect of the rule. They take the form:

from [base] towards [value].

Here, [base] is the unperturbed value of the behavioral parameter in the absence of signals, and [value] is its saturated response under maximum signal. The values should be stated with units, with a preference for microns for spatial units and minutes for time units.

When [base] and [effect] are not specified, parsers should assume a tenfold (one order-of-magnitude) change in behavior from the base parameter value:

$$b_M = 10\, b_0 \quad \text{for increasing responses}$$

$$b_m = 0.1\, b_0 \quad \text{for decreaseing responses}$$

Optional parameters 2: response form and parameters

Any rule can specify the form of its response function (currently linear or Hill) by appending:

with a [linear or Hill] response.

If the rule further specifies the parameters of the response, it takes the form:

with a [linear or Hill] response, with [parameters].

For linear responses, [parameters] takes the form:

with minimum threshold [value] and maximum threshold [value].

For Hill responses, [parameters] takes the form:

with half-max [value] and Hill power [value].

Optional statements: effect on dead cells

By default, we assume that the hypothesis rules apply to live cells only, to avoid non-physical behaviors such as nonzero motility or proliferation of dead cells. However, rules can be designated to apply to dead cells in addition to live cells with the optional statement:

Rule applies to dead cells.

Examples:

Here are examples of behavioral response statements with optional arguments noted in red.

- Oxygen increases cycle entry.
- Oxygen increases cycle entry from 7e-6 1/min towards 7e-4 1/min.
- Oxygen increases cycle entry from 7e-6 1/min towards 0.0007 1/min with a Hill response, with half-max 21.5 mmHg and Hill power 4.

- Pressure decreases cycle entry towards 0 1/min with a linear response, with minimum threshold 0 and maximum threshold 0.5.
- doxorubicin increases apoptosis.
- doxorubicin increases apoptosis towards 0.01 1/min with a Hill response, with half-max 0.1 and Hill power 2.
- Virus increases fusion to tumor cells. Rule applies to dead cells.

As an example of bundling multiple statements for a single cell type:

In malignant epithelial cells:

- Oxygen increases cycle entry towards 0.0007 1/min, with a Hill response, with half-max 21.5 mmHg and Hill power 4.
- Pressure decreases cycle entry towards 0.0 1/min, with a linear response, with minimum threshold 0 and maximum threshold 0.5.
- Dead increases debris secretion towards 1 1/min, with a Hill response, with half-max 0.1 and Hill power 4. Rule applies to dead cells.

Mathematical representation of model rules

With clearly defined behaviors and signals, and grammar to connect them, we can now uniquely map human-interpretable cell hypothesis statements onto mathematical expressions that make the grammar both human interpretable and computable. Moreover, our mathematical formulation allows new hypotheses to be directly added to models without modifying prior hypotheses, making our framing extensible and scalable as new knowledge is acquired.

Response functions

We use *response functions* to mathematically represent how a behavior varies with a signal towards its maximal (saturated) response. Our initial set of supported response functions are drawn from recurring forms in mathematical biology. In general, a response function R satisfies these properties:

1. $R(0) = 0$	In the absence of a signal, there is no response.
2. $R'(s) \geq 0$	Response increases monotonically as the signal increases.
3. $R(s) \to 1$ as $s \to \infty$	The response saturates at 100% for large amounts of signal.
4. $R(s) = 0$ if $s < 0$	For convenience, negative signals are truncated and ignored.

<u>Linear response function</u>

Many mathematical models use linear constitutive relations, so we define:

$$L(s; s_0, s_1) = \begin{cases} 0 & \text{if } s \leq s_0 \\ \dfrac{s - s_0}{s_1 - s_0} & \text{if } s_0 < s < s_1 \\ 1 & \text{if } s \geq s_1 \end{cases}$$

Here, s_0 and s_1 are (nonnegative) parameters of the response function governing where the response reaches its minimum and maximum values. For simplicity, we will call s_0 and s_1 the minimum and maximum response thresholds, respectively.

<u>Hill (sigmoidal) response function</u>

Hill functions (or sigmoidal functions) are commonly used in pharmacodynamics and in computational models of cell responses to chemical signals. We define:

$$H(s; s_{\text{half}}, \text{h}) = \frac{s^h}{s_{\text{half}}^h + s^h} = \frac{\left(\frac{s}{s_{\text{half}}}\right)^h}{1 + \left(\frac{s}{s_{\text{half}}}\right)^h} \text{ if } s \geq 0, \qquad \text{and } H(s) = 0 \text{ if } s < 0.$$

Here, s_{half} and h are (nonnegative) parameters governing where the response reaches half of its maximum value (s_{half}) and the steepness of the response (the Hill power h).

Multivariate Hill (sigmoidal) response function

Sometimes, we may need to consider the impact of multiple signals $\boldsymbol{s}$ (with half-maximum parameters $\boldsymbol{s}_{\text{half}}$ and Hill powers $\boldsymbol{h}$) on a behavior. Similarly to prior multi-variate response functions specifically designed to study drug combinations[97,98], we therefore introduce a multivariate version:

$$H_M(\boldsymbol{s}; \boldsymbol{s}_{\text{half}}, \boldsymbol{h}) = \frac{\sum_i \left(\frac{s_i}{s_{\text{half},i}}\right)^{h_i}}{1 + \sum_i \left(\frac{s_i}{s_{\text{half},i}}\right)^{h_i}},$$

with the additional stipulation that we replace s_i by $(s_i)^+ = \max(s_i,0)$ as needed. It satisfies:

- $H_M(\mathbf{0}) = 0$
- $H_M(0,\ldots,0,s_i,0,\ldots,0) = H(s_i)$ for any i
- $0 \leq H_M \leq 1$ for all $\boldsymbol{s}$
- $H_M(\boldsymbol{s}) \to 1$ as $|\boldsymbol{s}| \to \infty$

Thus, the signals can contribute to a response individually and in combination, and when only one signal is supplied, the response behaves as the more common Hill response function. This functional form has the benefit that individual Hill response parameters do not need to be recalibrated as new arguments (i.e., new biological knowledge and rules) are added. See **Fig. S1 (left).**

Multivariate linear response function

Similarly, we may need to consider the impact of multiple signals $\boldsymbol{s}$ (with thresholds $\boldsymbol{s}_0$ and $\boldsymbol{s}_1$) on a behavior. Motivated by the properties of the multivariate Hill function, we introduce a multivariate linear response function:

$$L_M(\boldsymbol{s}; \boldsymbol{s}_0, \boldsymbol{s}_1) = \min\left(1, \sum_i L(s_i; s_{0,i}, s_{1,i})\right),$$

with the additional stipulation that we replace s_i by $(s_i)^+ = \max(s_i, 0)$ as needed. It satisfies:

- $L(\mathbf{0}) = 0$
- $L_M(0, \ldots, 0, s_i, 0, \ldots 0) = L(s_i)$ for any i
- $0 \leq L_M \leq 1$ for all $\boldsymbol{s}$
- $L_M(\boldsymbol{s}) \to 1$ as $|\boldsymbol{s}| \to \infty$

Thus, the signals can contribute to a response individually and in combination, and when only one signal is supplied, the response behaves as the simpler linear response function. This functional form has the benefit that individual linear response parameters do not need to be recalibrated as new arguments (i.e., new biological knowledge and rules) are added. See **Fig. S1 (right).**

Approximating linear responses with Hill response functions

When curating and aggregating prior knowledge, we may encounter linear relationships and seek to approximate them with Hill response functions for a more unified framework. If a linear response function has thresholds s_0 and s_1, then we stipulate that:

- The half-max is $s_{\text{half}} = \frac{1}{2}(s_0 + s_1)$
- The Hill response function reaches 90% of its peak value at s_1

This can be satisfied when the Hill power is given by

$$h = \frac{\log\left(\frac{1-\epsilon}{\epsilon}\right)}{\log\left(\frac{s_1}{s_{\text{half}}}\right)},$$

where $0 < \epsilon < 1$ is a tolerance. (We set $\epsilon = 0.1$.) An Example is shown **Fig. S2**. We note that computationally, integer-valued Hill powers are at least tenfold faster in execution than non-integer powers, so it can be advantageous to round to the nearest whole number.

Approximating Hill responses with linear response functions

Similarly, when curating and aggregating prior knowledge, we may encounter Hill relationships and seek to approximate them as linear responses in a broader, unified framework. Moreover, this offers an opportunity for computational acceleration. If a Hill response function has half-max s_{half} and Hill power h, then we stipulate that:

- The right threshold s_1 is reached where the Hill response function reaches 90% of its peak value, and
- The half-max and thresholds are related by $s_{\text{half}} = \frac{1}{2}(s_0 + s_1)$

This can be satisfied by setting:

$$s_1 = s_{\text{half}} \left(\frac{1-\epsilon}{\epsilon}\right)^{\frac{1}{h}}$$

and

$$s_0 = 2\, s_{\text{half}} - s_1.$$

As before, $0 < \epsilon < 1$ is a tolerance, which we set to $\epsilon = 0.1$. An Example is show in **Fig. S2.**

Single hypothesis statement: S increases B or S decreases B

For any biological hypothesis statement of the form "*S* increases *B*" or "*S* decreases *B*", the behavior *B* can be associated with a phenotypic behavioral parameter *b*. The statement then takes the form of a linear interpolation in the nonlinear response function *R*:

$$b(s) = b_0 + (b_{\text{M}} - b_0)R(s) = \big(1 - R(s)\big) \cdot b_0 + R(s) \cdot b_M$$

Here, b_0 is the base level of the parameter (from the base behavioral phenotype), b_{M} is the maximal (saturated) response, and $R(s)$ is a user-selected response function.

Example: oxygen-driven cycling

The statement "oxygen increases cycle entry" (with a "base" value of $b_0 = 0.001\ \text{hr}^{-1}$ and a maximum rate of $b_{\text{M}} = 0.042\ \text{hr}^{-1}$) takes the form

$$r_{01} = 0.001 + (0.042 - 0.001)R(\text{pO}_2).$$

If we use a linear response function as in prior work[6,7,10,13,21] with minimum and maximum thresholds at 5 mmHg and 38 mmHg, respectively, then the response function takes the form

$$r_{01} = \begin{cases} 0.001 & \text{if } \mathrm{pO_2} \leq 5 \text{ mmHg} \\ 0.001 + (0.042 - 0.001)\left(\frac{\mathrm{pO_2} - 5}{38 - 5}\right) & \text{if } 5 < \mathrm{pO_2} < 38 \text{ mmHg} \\ 0.042 & \text{if } \mathrm{pO_2} \geq 38 \text{ mmHg} \end{cases}$$

If we use a Hill response function (with a half-max of 21.5 mmHg, and Hill power of 4), the mathematical rule takes the form

$$r_{01} = 0.001 + (0.042 - 0.001)\frac{(\mathrm{pO_2})^4}{21.5^4 + (\mathrm{pO_2})^4}$$

Example: mechanoregulation of cycling

The statement "pressure decreases cycle entry" (with a "base" value of $b_0 = 0.001$ hr^{-1} and a maximally inhibited rate of $b_M = 0$ hr^{-1}) takes the form

$$r_{01} = 0.001 + (0 - 0.001)R(p).$$

If we use a Hill response function (with a half-max of 0.25, and Hill power of 3), the mathematical rule takes the form

$$r_{01} = 0.001 + (0.0 - 0.001)\frac{p^3}{0.25^3 + p^3} = 0.001\left(1 - \frac{p^3}{0.25^3 + p^3}\right)$$

Multiple hypothesis statements: S_1 increases B, S_2 increases B

For any biological hypothesis statement of the form "*S* decreases *B*", the behavior *B* can be associated with a phenotypic behavioral parameter *b*. The statement then takes the form:

$$b(s_1, s_2) = b_0 + (b_M - b_0)R(s_1, s_2)$$

Here, b_0 is the base level of the parameter (its value in the absence of any signals), b_M is the maximal (saturated) response, and $R(\mathbf{s})$ is a user-selected response function. We use the multivariate Hill response function H_M for $R(\mathbf{s})$.

Example: oxygen- and hormone-dependent cycling

The statements "oxygen increases cycle entry" and "estrogen increases cycle entry" (with a "base" value of $b_0 = 0.001$ hr^{-1} and a maximum rate of $b_M = 0.042$ hr^{-1}) takes the form

$$r_{01} = 0.001 + (0.042 - 0.001)\, H_M(\mathrm{pO_2}, e).$$

If we use an extended Hill response function (where oxygen has a half-max of 21.5 mmHg and Hill power of 4, and nondimensionalized estrogen has a half-max of 0.5 and Hill power of 3), the mathematical rule takes the form

$$r_{01} = 0.001 + (0.042 - 0.001)\frac{\left(\frac{\mathrm{pO_2}}{21.5}\right)^4 + \left(\frac{e}{0.5}\right)^3}{1 + \left(\frac{\mathrm{pO_2}}{21.5}\right)^4 + \left(\frac{e}{0.5}\right)^3}.$$

Multiple competing hypothesis statements: S1 increases B, S2 decreases B

For competing biological hypothesis statements of the form "S_1 increases *B*" and "S_2 decreases *B*", if the behavior has the associated phenotypic parameter *b*, then we use the form:

$$b(s_1, s_2) = \left(b_0 + (b_M - b_0) \frac{s_1^p}{(s_1^*)^p + s_1^p} \right) + \left(b_m - \left(b_0 + (b_M - b_0) \frac{s_1^p}{(s_1^*)^p + s_1^p} \right) \right) \frac{s_2^q}{(s_2^*)^q + s_2^q}$$

Here, b_0 is the base level of the parameter (from the base behavioral phenotype in the absence of signals), b_M is the maximal response to the promoting signal s_1 (with half-max s_1^* and Hill power p), and b_m is the maximal response to the inhibiting signal s_2 (with half-max s_2^* and Hill power q).

We can write this more simply with:

$$U = \frac{s_1^p}{(s_1^*)^p + s_1^p} \quad , \quad D = \frac{s_2^q}{(s_2^*)^q + s_2^q}$$

giving the overall response as a bilinear interpolation:

$$b(s_1, s_2) = (1 - D) \cdot [\, (1 - U) \cdot b_0 + U \cdot b_M] + D \cdot b_m$$

In this formulation, the promoting signal s_1 pushes the behavior towards a "target" value that is then subject to inhibition by s_2. Notice that:

- If $s_2 = 0$, then $D = 0$ and b behaves as the regular Hill response to the promoting signal s_1.
- If $s_1 = 0$, then $U = 0$ and b behaves as the regular Hill response to the inhibiting signal s_2.
- In the absence of either signal, $b(0,0) = b_0$.
- As $s_1 \to \infty$ and $s_2 \to \infty$, then $b(s_1, s_2) \to b_m$.

Example: oxygen-driven cycling with negative mechanofeedback

Suppose "oxygen increases cycle entry" and "pressure decreases cycle entry" with parameters as in the prior examples. Then our functional form is:

$$U = \frac{(\mathrm{pO_2})^4}{21.5^4 + (\mathrm{pO_2})^4}, \qquad D = \frac{p^3}{0.25^3 + p^3}$$

$$b(\mathrm{pO_2}, p) = (1 - D) \cdot [(1 - U) \cdot 0.001 + U \cdot 0.042] + D \cdot 0$$

See **Fig. S3 (left)**.

Example: "contradictory" observations on cell migration

Suppose a cell is migrating by chemotaxis towards a (scaled to be nondimensional) chemokine c, where we observe:

- Migration becomes less random and more directed as the chemokine concentration increases (it is more difficult to sense a chemical gradient in low concentrations)
- Migration becomes more random and less directed for extremely high chemokine concentrations (high concentrations can saturate the cell's chemical receptors, making it difficult to sense the chemical gradient)

In the cell behavior grammar, these statements become:

- c increases migration bias
  - We will use a base migration bias of 0.01 and maximum bias of 1.
  - If the effect reaches its maximum at $c = 0.2$, we set the linear response minimum and maximum thresholds to $s_0 = 0$ and $s_1 = 0.2$. For the Hill response approximation, we set $s_{\mathrm{half}} = 0.1$ and $h = 3$
- c decreases migration bias
  - We will use a minimum migration bias of 0.

- If the effect is first notable at $c = 0.8$ and increases towards its maximum at $c = 1$, we use minimum and maximum response thresholds of $s_0 = 0.8$ and $s_1 = 1$ for a linear response. For the Hill response approximation, we set $s_{\text{half}} = 0.9$ and $h = 21$

These seemingly contradictory statements jointly create non-monotonic behavior:

$$b(c) = \big(1 - H(c; 0.9{,}21)\big) \cdot \big[\big(1 - H(c; 0.1{,}3)\big) \cdot 0.01 + H(c; 0.1{,}3) \cdot 1\big] + H(c; 0.9{,}21) \cdot 0$$

We plot the overall response using both Hill responses (red) and linear response (dotted black) in **Fig. S3 (right).**

General form: multiple promoting and inhibiting rules

Suppose we have the statements:

- u_1 increases B (with half-max u_1^* and Hill power p_1)
- u_2 increases B (with half-max u_2^* and Hill power p_2)
- ...
- u_m increases B (with half-max u_m^* and Hill power p_m)
- d_1 decreases B (with half-max d_1^* and Hill power q_1)
- d_2 decreases B (with half-max d_2^* and Hill power q_2)
- …
- d_n decreases B (with half-max d_m^* and Hill power q_n)

Here, let $b_M$be the maximum value of the parameter b (under the combined influence of the up-regulating signals $\boldsymbol{u}$), let b_0 be its base value in the absence of signals, and let b_m be its minimum value (under the combined influence of the down-regulating signals $\boldsymbol{d}$).

We define the total up response as:

$$U = H_M(\boldsymbol{u}; \boldsymbol{u}_{\text{half}}, \boldsymbol{p}) = \frac{\left(\frac{u_1}{u_1^*}\right)^{p_1} + \left(\frac{u_2}{u_2^*}\right)^{p_2} + \cdots + \left(\frac{u_m}{u_m^*}\right)^{p_m}}{1 + \left(\frac{u_1}{u_1^*}\right)^{p_1} + \left(\frac{u_2}{u_2^*}\right)^{p_2} + \cdots + \left(\frac{u_m}{u_m^*}\right)^{p_m}}$$

and the total down response as:

$$D = H_M(\boldsymbol{d}; \boldsymbol{d}_{\text{half}}, \boldsymbol{q}) = \frac{\left(\frac{d_1}{d_1^*}\right)^{q_1} + \left(\frac{d_2}{d_2^*}\right)^{q_2} + \cdots + \left(\frac{d_n}{d_n^*}\right)^{q_n}}{1 + \left(\frac{d_1}{d_1^*}\right)^{q_1} + \left(\frac{d_2}{d_2^*}\right)^{q_2} + \cdots + \left(\frac{d_n}{d_n^*}\right)^{q_n}}.$$

Using these, we combine the overall response of the behavioral parameter as bilinear interpolation in the nonlinear up- and down-responses U and D:

$$b(\boldsymbol{u}, \boldsymbol{d}) = (1 - D) \cdot [(1 - U) \cdot b_0 + U \cdot b_M\,] + D \cdot b_m$$

Notice that:

- In the presence of up-regulating signals only, this reduces to the multivariate Hill response to $\boldsymbol{u}$.
- In the presence of down-regulating signals only, this becomes the multivariate Hill response to $\boldsymbol{d}$.
- Generally, the combined up-regulating signals sets a “target” value of the parameter, which can then be inhibited by the combined down-regulating signals.

Note also that adding and removing individual rules to the form does not require alteration to the remaining rules. In this release, we use multivariate Hill response functions for clarity, but mixed linear and Hill responses could be used in the future.

Example:

We combine three hypothesis statements from prior examples that modulate cycle entry:

- Oxygen increases cycle entry
- Estrogen increases cycle entry
- Pressure decreases cycle entry

The combined mathematical form for the cycle entry rate r_{01} is:

$$U = \frac{\left(\frac{\mathrm{pO_2}}{21.5}\right)^4 + \left(\frac{\mathrm{e}}{0.5}\right)^3}{1 + \left(\frac{\mathrm{pO_2}}{21.5}\right)^4 + \left(\frac{\mathrm{e}}{0.5}\right)^3} \quad , \quad D = \frac{\left(\frac{p}{0.25}\right)^3}{1 + \left(\frac{p}{0.25}\right)^3}$$

$$r_{01} = (1 - D) \cdot [b_0 + (b_M - b_0)] + D \cdot b_m$$

$$b_0 = 0.001\ \mathrm{hr}^{-1} \quad , \quad b_M = 0.042\ \mathrm{hr}^{-1} \quad , \quad b_m = 0\ \mathrm{hr}^{-1}.$$

Reference PhysiCell implementation

We implemented support for the grammar in PhysiCell version 1.12.0[99], and version 1.14.2 (and later) supports the grammar as specified in this manuscript. Rules are imported at the start of a simulation using a compact CSV format, parsed, and stored for each cell type. Rules are stored in a ruleset data structure, with a separate ruleset for each cell definition:

1. For each modulated behavior (with corresponding parameter b), store a set of rules:
   a. For each rule, we store:
      i. The "direction" of the response (increases/promotes or decreases/inhibits the behavior)
      ii. The signal used in the rule
      iii. The half-max, Hill parameter, and maximal value of the behavior of the parameter
      iv. Whether it applies to dead cells
   b. Based upon all the up-regulating rules, store the largest maximum parameter value b_M
   c. Based upon all the down-regulating rules, store the lowest minimum parameter value b_m

To execute a set of rules (for a single cell):

1. For each modulated behavior (with corresponding parameter b):
   a. For each rule:
      i. Sample the corresponding signal s
      ii. Query its Hill parameter h and half-max s_{half}
      iii. Compute $\left(\frac{s}{s_{\mathrm{half}}}\right)^h$
      iv. If the rule increases (promotes) the behavior, add the contribution from iii to U in the generalized multivariate response function. Otherwise, add the contribution to D.
   b. Query the base parameter value b_0, the maximum parameter value b_M, and the minimum parameter value b_m.
   c. Compute the modulated parameter value via $b = (1 - D) \cdot [(1 - U) \cdot b_0 + U \cdot b_M] + D \cdot b_m$
   d. Update the phenotypic parameter b for the cell.

At the start of each simulation step, each cell evaluates its individual rules (based on its current cell type) as noted above to set its current phenotype parameters, and then runs its standard phenotype processes.

We envision that other agent-based simulation frameworks (e.g., Chaste, CompuCell3D, Biocellion, Morpheus, and Tissue Simulation Toolkit) can independently implement this framework so long as they (1)

implement the reference cell process models, (2) define cell types, (3) can generate compatible dictionaries of signals and behaviors, (4) can parse the rules, and (5) use these to modulate the cell behavioral parameters using the reference multivariate response functions as noted above. For an up-front development cost, simulation frameworks could advance reproducibility and support cross-model validation.

We note that PhysiCell currently has only implemented the Hill response functions; linear responses are planned for a later release.

UNCERTAINTY QUANTIFICATION IN PHYSICELL MODELS

Biological variability in multicellular systems arises from stochastic processes, including gene expression, signaling, and microenvironment fluctuations, contributing to heterogeneity among otherwise identical cells. These sources of intrinsic and extrinsic variability are collectively referred to as biological noise. Rather than being treated as experimental error or nuisance variability, biological noise is increasingly recognized as a fundamental component of biological systems, driving heterogeneous behaviors even among genetically identical cells under the same conditions. PhysiCell inherently account for aleatory uncertainty – inherent randomness in biological systems – by incorporating stochastic rules that govern cell behaviors, such as proliferation and migration. This incorporation of aleatory uncertainty in PhysiCell provides a foundation for advanced uncertainty quantification techniques.

Uncertainty quantification (UQ) in computational modeling aims to reduce epistemic uncertainty – uncertainty due to incomplete knowledge – and quantify the combined effects of aleatory and epistemic uncertainties. UQ supports multiple modeling objectives, including parameter prioritization, distributed outcome quantification, and parameter calibration. In this work, we demonstrate these objectives through sensitivity analysis, which identifies how variations in model parameters influence systems outcomes, facilitating parameter prioritization and distributional outcome quantification. Calibration techniques align parameter values with experimental data, mitigating epistemic uncertainty. Additionally, model selection addresses ontological uncertainty – uncertainty arising from model structure or assumptions – by evaluating how different model formulations impact prediction, recognizing that simplifications or missing mechanism may affect results. These strategies have informed the development of UQ_PhysiCell, a package designed to address uncertainty quantification challenges in the PhysiCell modeling framework.

Comprehensive Analyses with UQ_PhysiCell

We developed UQ_PhysiCell [https://pypi.org/project/uq-physicell/], a Python package designed to facilitate comprehensive analysis of PhysiCell models, including sensitivity analysis, calibration, model selection, and validation. The primary goal of UQ_PhysiCell is to enable uncertainty quantification (UQ) across the entire input space of PhysiCell models—encompassing parameters, initial conditions, and rules—using either a single desktop or high-throughput computing resources. This package is built to seamlessly integrate with popular Python libraries such as SALib[100], pyABC[101], emcee[102], pymc3[103], and UQpy[104], providing a flexible and modular framework for extending its capabilities and fostering reproducibility in computational modeling workflows.

In this work, we applied UQ_PhysiCell to perform a local sensitivity analyses of the hypoxia model and the simple tumor-immune model.

Local Sensitivity Analyses of the Hypoxia model

To evaluate the impact of parameter variations on model outputs, we conducted a local sensitivity analysis using a reference parameter set $\theta^\star$ (**Fig. 2A** in the main manuscript). The goal was to quantify how changes in individual parameters influence the quantities of interest (QoIs) and identify the most influential

parameters. We select four non-zero parameters for each rule: "base" value, half-max, maximum value, and Hill power. These parameters were associated with the following rules:

- Oxygen increases cycle entry (rule1 and rule5 for non-motile and motile tumor cells),
- Pressure decreases cycle entry (rule2 and rule6 for non-motile and motile tumor cells),
- Oxygen decreases necrosis (rule3 and rule7 for non-motile and motile tumor cells),
- Oxygen decreases transformation from non-motile to motile cell (rule4 for non-motile tumor cells),
- Oxygen increases transformation from motile to non-motile (rule8 for motile tumor cells).

This generated a total of 24 parameters. For each parameter, we applied a multiplicative perturbation around the reference value θ^* at levels of 1%, 5%, 10% and 20%:

$$\theta_i = \theta^* + \delta\theta^*, \delta \in \{\pm 0.01, \pm 0.05, \pm 0.10, \pm 0.20\}$$

We performed 50 replicates for the reference parameter set and for each of the 192 perturbed parameter set, totaling 9,650 simulations. **Fig. S4** presents the convergence of the mean and standard deviation for each QoI across replicates. Each simulation stored cell population data and the distance of each cell from the domain center over a 5-day period (with a time step of one hour), generating a dataset of 21.38GB (Available in https://doi.org/10.5281/zenodo.14590311).

The QoIs were defined as follows:

- Temporal changes in cell populations,
- Spatial distribution differences between non-motile and motile tumor cells.

For population dynamics, we calculated the areas under the curve (AUC) for live tumor cells, dead tumor cells, live motile cells, and dead motile cells (**Fig. 2C** in the main manuscript). For spatial differences, we calculated the Wasserstein distance to compare the distributions of cell distances from the center between live tumor cells and live motile cells on day 5 (**Fig. 2C** in the main manuscript).

Across the multiplicative perturbations, the QoIs generally maintained their central tendency; however, deviations increased with higher levels of perturbation (**Fig. 2D** in the main manuscript). To detect the impact of individual parameters on each QoI, we calculated the sensitivity index S_i for each parameter θ_i:

$$S_i = \frac{|f(\theta^*) - f(\theta_i)|}{\max_{\delta}|f(\theta^*) - f(\theta_i)|}$$

where $|.|$ represents the norm of the difference between QoIs $f(.)$ derived from the reference parameter set θ^* and the perturbed parameter set θ_i. The index was normalized by the maximum observed difference across all perturbation levels δ. For each parameter, we calculated the mean sensitivity index across all perturbations and summarized the mean and standard deviation of all QoIs (**Fig. 2E** in the main manuscript). The individual sensitivity indices associated with each QoI are available at:

https://github.com/PhysiCell-Models/grammar_samples/blob/main/Sensitivity_Analysis/SA_script_ex1.ipynb.

Local Sensitivity Analyses of the Simple Tumor-Immune Model

To evaluate the impact of parameter variations in this model, we applied a local sensitivity analysis based on a reference parameter set $\theta^\star$ (**Fig. S11A**). This analysis aimed to identify the influence of individual parameters on the selected QoIs and determine the most impactful parameters.

For this model, the parameters were associated with the following behaviors:

- The initial quantity of macrophages and CD8 T cells with ratio 1:1 (labeled as *IC_immune_cells*),
- In tumor cells:
    - Damage increases apoptosis (labeled as *damage_apop_sat*, *damage_apop_hfm*, and *damage_apop_hp*).

- Death of tumor cells increases debris secretion (labeled as *dead_debris_sat*, *dead_debris_hfm*, and *dead_debris_hp*).
- In macrophages:
  - Phagocytosis rate of necrotic and apoptotic cells (labeled as *phago_rate_nec* and *phago_rate_apop*).
  - Oxygen increases the pro-inflammatory factor secretion (labeled as *mac_oxy_proinf_sat*,*mac_oxy_proinf_hfm*, and *mac_oxy_proinf_hp*).
  - Oxygen decreases the anti-inflammatory factor secretion (labeled *mac_antinf_secretion_base*,*mac_oxy_antinf_secretion_hfm*, and *mac_oxy_antinf_secretion_hp*).
- In CD8 T cells:
  - Total attack duration of CD8 T cells (labeled as *attack_duration*).
  - Base migration speed (labeled as *cd8_migr_speed_base*).
  - Pro-inflammatory factor increases attack of malignant epithelial cells (labeled as *cd8_proinf_attack_sat, cd8_proinf_attack_hfm,* and *cd8_proinf_attack_hp*).
  - Anti-inflammatory factor decreases attack of malignant epithelial cells (labeled as *cd8_antinf_attack_hfm*and *cd8_antinf_attack_hp*).
  - Anti-inflammatory factor decreases migration speed (labeled as *cd8_antinf_speed_hfm* and *cd8_antinf_speed_hp*).
  - Contact with tumor cells decreases migration speed (labeled as *cd8_contact_speed_hfm* and *cd8_contact_speed_hp*).

A total of 26 parameters were perturbed by applying multiplicative changes of ±1%, ±5%, ±10%, and ±20% similar to the previous analyses. Fifty replicates were conducted for the reference parameter set and each perturbed set, generating a total of 10,450 simulations. Two simulations resulted in zero remaining cells, making certain QoIs incomputable. These were thus excluded from downstream analyses. **Fig. S12** shows the variation of QoIs across replicates, while **Fig. S11D** illustrates the variation in QoIs under multiplicative perturbations in the 26-dimensional parameter space. Data from these simulations included cell population dynamics, spatial distributions, and pro- and anti-inflammatory factor secretion rates, recorded over 5 days using a time step of one hour, resulting in a dataset of 15.06GB (Available in https://doi.org/10.5281/zenodo.14590311).

The QoIs for this model were defined as:

- Temporal changes in cell population and pro- and anti- inflammatory factor secretion,
- Spatial distribution differences between tumor cells, macrophages, and CD8 T cells.

We computed the AUC for live and dead tumor cells and the average pro- and anti-inflammatory factor secretion rates (**Fig. S11B**). To evaluate spatial patterns, we calculated the Wasserstein distance to compare the distributions of cell distances from the center among tumor cells, macrophages, and CD8 T cells on day 5, as shown in **Fig. S11C**.

The sensitivity of each parameter was quantified using the sensitivity indices, calculated in a manner similar to the analyses performed in the hypoxia model. The mean sensitivity index across all perturbations was computed for each parameter, with the mean and standard deviation of all QoIs summarized in **Fig. S11E**. The individual sensitivity indices associated with each QoI are available at:

https://github.com/PhysiCell-Models/grammar_samples/blob/main/Sensitivity_Analysis/SA_script_ex3.ipynb

RESOURCES FOR LEARNING AND USING PHYSICELL

We have developed online training curriculum for developing and using PhysiCell models, using the rules grammar presented in this paper. New learners (including those with agent-based modeling expertise, but

who have not yet learned PhysiCell or the grammar) should use the ***PhysiCell Essentials*** short course, available at https://physicell.org/Training.html#essentials.

The ***Essentials*** course includes an introduction to agent-based modeling, the grammar, and hands-on examples that can be completed in a web browser without requiring software installation or any writing and compiling of code. These tutorials (including slides, downloadable code, and recordings), can be completed asynchronously, or as part of a virtual or in-person workshop or hackathon, such as those we ran virtually in 2021-2023[105-107] and in person in at the University of California at Irvine in 2022[108] and 2024[109], at the Centre Européen de Calcul Atomique et Moléculaire (CECAM) in 2023[110], at Northwestern University in 2023[111], and our upcoming workshop at the Society for Mathematical Biology Annual Meeting in July 2025. Announcements for ongoing and future training workshops will be posted at:

https://physicell.org/Training.html

We are also revising curriculum for more advanced learners including:

- ***Integration of Boolean Networks with PhysiBoSS*** (for users who wish to integrate intracellular Boolean networks into PhysiCell agents via PhysiBoSS[112])
- ***Advanced PhysiCell Modeling*** (for C++-based development of custom functions and visualizations, as well as use of advanced C++-based extensions for extracellular matrix[113,114] and related advanced topics)
- ***PhysiCell for Developers*** (for future extension developers and new core developers).

Future training materials will include additional purpose-built training apps[3], as well as tutorials and guides by members of our modeling community and software ecosystem[112].

Lastly, we have a supportive community who readily provide technical support and modeling tips on a dedicated Slack workspace, which can be joined at:

https://join.slack.com/t/physicellcomm-sf93727/shared_invite/zt-qj1av6yd-yVeer8VkQaNDjDz7fF00jA

FULL MODEL ANNOTATIONS

Modeling the progression of hypoxia in a metastatic tumor

This model is called "hypoxia" in https://github.com/PhysiCell-Models/grammar_samples

Cell Types and Rules

Cell type: tumor

BASE BEHAVIORS (AND PARAMETER VALUES)

Cycling: 7.20×10^{-4} min^{-1} (live model).

Apoptosis: 5.32×10^{-5} min^{-1}.

Necrosis: 2.80×10^{-3} min^{-1}.

Migration: No.

Secretion: 10 min^{-1} of oxygen uptake.

RULES

- oxygen increases cycle entry from 0.00072 towards 0.0072 with a Hill response, with half-max 21.5 and Hill power 4
- pressure decreases cycle entry from 0.00072 towards 0 with a Hill response, with half-max 0.25 and Hill power 3
- oxygen decreases necrosis from 0.0028 towards 0 with a Hill response, with half-max 3.75 and Hill power 8.
- oxygen decreases transform to motile tumor from 0.001 towards 0 with a Hill response, with half-max 6.75 and Hill power 8.

Cell type: motile tumor

BASE BEHAVIORS (AND PARAMETER VALUES)

Cycling: 7.20×10^{-4} min^{-1} (live model).

Apoptosis: 5.32×10^{-5} min^{-1}.

Necrosis: 2.80×10^{-3} min^{-1}.

Migration: 0.47 micron/min, with persistence time 15 min, and migration bias 0.18 towards oxygen gradient.

Secretion: 10 min^{-1} of oxygen uptake.

RULES

- oxygen increases cycle entry from 0.00072 towards 0.0072 with a Hill response, with half-max 21.5 and Hill power 4.
- pressure decreases cycle entry from 0.00072 towards 0 with a Hill response, with half-max 0.25 and Hill power 3.
- oxygen decreases necrosis from 0.0028 towards 0 with a Hill response, with half-max 3.75 and Hill power 8.
- oxygen increases transform to tumor from 0 towards 0.005 with a Hill response, with half-max 6.75 and Hill power 8.

Other implementation details

COMPUTATIONAL DOMAIN

The model simulates a 3D computational domain of $1500\ \mu m \times 1500\ \mu m \times 20\ \mu m$.

INITIALIZATION

The simulation begins with 2,000 viable tumor cells randomly seeded within a virtual disk of 400 microns in radius.

MICROENVIRONMENT

The domain is initialized with a uniform oxygen partial pressure of 38 mmHg, maintained as a constant source at the boundaries.

Fibroblast-mediated invasion of neoplastic cells

This model is called "epi_caf_invasion" in https://github.com/PhysiCell-Models/grammar_samples

Cell Types and Rules

Cell type: epithelial_normal

BASE BEHAVIORS (AND PARAMETER VALUES)

Cycling: No.

Apoptosis: 5.32×10^{-5} min^{-1}

Necrosis: No.

Motility: No.

Secretion: No.

RULES

- pressure decreases cycle entry from 0 towards 0 with a Hill response, with half-max 0.5 and Hill power 4.
- ecm increases transform to mesenchymal_normal from 0 towards 0.01 with a Hill response, with half-max 0.01 and Hill power 4.

Cell type: mesenchymal_normal

BASE BEHAVIORS (AND PARAMETER VALUES)

Cycling: No.

Apoptosis: 5.32×10^{-6} min^{-1}

Necrosis: No.

Motility: 0.249909 micron/min, with persistence time 10 min, and no chemotaxis.

Secretion: 1 min^{-1} of inflammatory_signal secretion.

Mechanics: 0.1 micron/min of cell-cell adhesion strength with all cells.

Transition to: epithelial_normal cell with a rate of 0.01 min^{-1};

mesenchymal_tumor cell with a rate of 0.001 min^{-1}.

RULES

- ecm decreases migration speed from 0.249909 towards 0.249678 with a Hill response, with half-max 0.0595916 and Hill power 1.22413.
- ecm increases migration speed from 0.249909 towards 3.78565 with a Hill response, with half-max 3.11941 and Hill power 9.99815.
- inflammatory_signal decreases transform to epithelial_normal from 0.01 towards 0 with a Hill response, with half-max 0.2 and Hill power 4.

Cell type: fibroblast

BASE BEHAVIORS (AND PARAMETER VALUES)

Cycling: No.

Apoptosis: No.

Necrosis: No.

Motility: 8.04×10^{-6} micron/min, with persistence time 10 min, and no chemotaxis.

Secretion: 0.1 min^{-1} of ecm secretion with secretion target of 10 ecm density.

RULES

- ecm decreases migration speed from 8.03971e-06 towards 6.90516e-06 with a Hill response, with half-max 2.07833 and Hill power 1.1551.
- ecm increases migration speed from 8.03971e-06 towards 3.47055 with a Hill response, with half-max 9.99968 and Hill power 1.15302.

Cell type: epithelial_tumor

BASE BEHAVIORS (AND PARAMETER VALUES)

Cycling: 1×10^{-3} min^{-1} (live model)

Apoptosis: 5.32×10^{-5} min^{-1}

Necrosis: No.

Motility: No.

Secretion: No.

RULES

- pressure decreases cycle entry from 0.001 towards 0 with a Hill response, with half-max 1 and Hill power 4.

- ecm increases transform to mesenchymal_tumor from 0 towards 0.01 with a Hill response, with half-max 0.01 and Hill power 4.

Cell type: mesenchymal_tumor

BASE BEHAVIORS (AND PARAMETER VALUES)

Cycling: No.

Apoptosis: 5.32×10^{-6} min^{-1}

Necrosis: No.

Motility: 0.249909 micron/min, with persistence time 10 min, and no chemotaxis.

Secretion: 1 min^{-1} of inflammatory_signal secretion.

Mechanics: 0.1 micron/min of cell-cell adhesion strength with all cells.

Transition to: epithelial_tumor cell with a rate of 0.01 min^{-1}.

RULES

- ecm decreases migration speed from 0.249909 towards 0.249678 with a Hill response, with half-max 0.0595916 and Hill power 1.22413.
- ecm increases migration speed from 0.249909 towards 3.78565 with a Hill response, with half-max 3.11941 and Hill power 9.99815.
- inflammatory_signal decreases transform to epithelial_tumor from 0.01 towards 0 with a Hill response, with half-max 0.2 and Hill power 4.

Cell type: other_tissue

BASE BEHAVIORS (AND PARAMETER VALUES)

Cycling: No.

Apoptosis: No.

Necrosis: No.

Motility: No.

Secretion: No.

RULES

No rules.

Other implementation details

COMPUTATIONAL DOMAIN

The model simulates a 3D computational domain of $1600\ \mu m \times 1600\ \mu m \times 20\ \mu m$.

INITIALIZATION

Custom initialization: coculture, PDAC01, PDAC02.

MICROENVIRONMENT

The domain is initialized with no concentration of inflammatory_signal or ecm, and cells act as sources and sinks of these substrates according to their phenotype states defined by the base behavior and rules.

Base tumor-immune model

This model is called “tumor_immune_base” in https://github.com/PhysiCell-Models/grammar_samples

Cell Types and Rules

Cell type: tumor

BASE BEHAVIORS (AND PARAMETER VALUES)

Cycling: 7.20×10^{-4} min^{-1} (live model).

Apoptosis: 5.32×10^{-5} min^{-1}.

Necrosis: 2.80×10^{-3} min^{-1}.

Motility: No.

Secretion: 10 min^{-1} of oxygen uptake.

RULES

- oxygen increases cycle entry from 0.00072 towards 0.0072 with a Hill response, with half-max 21.5 and Hill power 4.
- pressure decreases cycle entry from 0.00072 towards 0 with a Hill response, with half-max 0.25 and Hill power 3.
- oxygen decreases necrosis from 0.0028 towards 0 with a Hill response, with half-max 3.75 and Hill power 8.
- damage increases apoptosis from 5.31667e-05 towards 0.023 with a Hill response, with half-max 45 and Hill power 16.
- dead increases debris secretion from 0 towards 0.017 with a Hill response, with half-max 0.1 and Hill power 10. Rule applies to dead cells.

Cell type: macrophage

BASE BEHAVIORS (AND PARAMETER VALUES)

Cycling: No.

Apoptosis: No.

Necrosis: No.

Motility: 1 micron/min, with persistence time 5 min, and migration bias 0.25 towards debris gradient.

Secretion: 10 min^{-1} of oxygen uptake, 1 min^{-1} of debris uptake, and 10 min^{-1} of anti-inflammatory factor secretion.

Phagocytosis: 0.1 min^{-1} for apoptotic cells and 0.017 min^{-1} for necrotic cells.

RULES

- oxygen increases pro-inflammatory factor secretion from 0 towards 10 with a Hill response, with half-max 12 and Hill power 16.
- oxygen decreases anti-inflammatory factor secretion from 10 towards 0 with a Hill response, with half-max 12 and Hill power 16.

Cell type: CD8 T cell

BASE BEHAVIORS (AND PARAMETER VALUES)

Cycling: No.

Apoptosis: No.

Necrosis: No.

Motility: 1.0 micron/min, with persistence time 5 min, and migration bias 0.5 towards pro-inflammatory factor gradient.

Secretion: 10 min^{-1} of oxygen uptake, 1 min^{-1} of pro-inflammatory factor uptake, and 1 min^{-1} of anti-inflammatory factor uptake.

Attack: 0.1 min^{-1} attack rate to tumor cells, with 15 min of attack duration, and 1 min^{-1} of damage rate.

RULES

- anti-inflammatory factor decreases attack tumor from 0.1 towards 0 with a Hill response, with half-max 0.5 and Hill power 8.
- pro-inflammatory factor increases attack tumor from 0.1 towards 1 with a Hill response, with half-max 0.5 and Hill power 8.
- anti-inflammatory factor decreases migration speed from 1 towards 0 with a Hill response, with half-max 0.5 and Hill power 8.
- contact with tumor decreases migration speed from 1 towards 0 with a Hill response, with half-max 0.5 and Hill power 8.

Other implementation details

COMPUTATIONAL DOMAIN

The model simulates a 3D computational domain of $1500\ \mu m \times 1500\ \mu m \times 20\ \mu m$.

INITIALIZATION

The simulation begins with 2,000 viable tumor cells randomly seeded within a virtual disk of 400 microns in radius. Additionally, 100 macrophages and 100 CD8 T cells are randomly seeded within an annulus with an inner radius of 450 microns and an outer radius of 500 microns, relative to the center of the domain.

MICROENVIRONMENT

The domain is initialized with a uniform oxygen partial pressure of 38 mmHg, maintained as a constant source at the boundaries. No initial concentration of debris, pro-inflammatory factor, or anti-inflammatory factors are considered, and cells act as sources and sinks of these substrates according to their phenotype states defined by the base behavior and rules.

Extended tumor-immune model

This model is called "tumor_immune_extended" in https://github.com/PhysiCell-Models/grammar_samples

Cell Types and Rules

Cell type: tumor

BASE BEHAVIORS (AND PARAMETER VALUES)

Cycling: 7.20×10^{-4} min^{-1} (live model).

Apoptosis: 5.32×10^{-5} min^{-1}.

Necrosis: 2.80×10^{-3} min^{-1}.

Motility: No.

Secretion: 10 min^{-1} of oxygen uptake.

RULES

- pressure decreases cycle entry from 0.00072 towards 0 with a Hill response, with half-max 0.25 and Hill power 3.
- oxygen increases cycle entry from 0.00072 towards 0.00072 with a Hill response, with half-max 21.5 and Hill power 4.
- oxygen decreases necrosis from 0.0028 towards 0 with a Hill response, with half-max 3.75 and Hill power 8.
- damage increases apoptosis from 5.31667e-05 towards 0.023 with a Hill response, with half-max 45 and Hill power 16.
- dead increases debris secretion from 0 towards 0.017 with a Hill response, with half-max 0.1 and Hill power 10.

Cell type: M0 macrophage

BASE BEHAVIORS (AND PARAMETER VALUES)

Cycling: No.

Apoptosis: 7.20×10^{-5} min^{-1}.

Necrosis: No.

Motility: 1.0 micron/min, with persistence time 5 min, and migration bias 0.25 towards debris gradient.

Secretion: 10 min^{-1} of oxygen uptake and 1 min^{-1} of debris uptake.

Phagocytosis: 0.1 min^{-1} for apoptotic cells and 0.017 min^{-1} for necrotic cells.

RULES

- contact with dead cell decreases migration speed from 1 towards 0.1 with a Hill response, with half-max 0.1 and Hill power 4.
- contact with dead cell increases transform to M1 macrophage from 0 towards 0.05 with a Hill response, with half-max 0.1 and Hill power 10.
- dead increases debris secretion from 0 towards 0.017 with a Hill response, with half-max 0.1 and Hill power 10.

Cell type: M1 macrophage

BASE BEHAVIORS (AND PARAMETER VALUES)

Cycling: No.

Apoptosis: 7.20×10^{-5} min^{-1}.

Necrosis: No.

Motility: 1.0 micron/min, with persistence time 5 min, and migration bias 0.25 towards debris gradient.

Secretion: 10 min^{-1} of oxygen uptake, 1 min^{-1} of debris uptake, 1 min^{-1} of IFN-gamma uptake, and 10 min^{-1} of IFN-gamma secretion.

Phagocytosis: 0.1 min^{-1} for apoptotic cells and 0.017 min^{-1} for necrotic cells.

RULES

- contact with dead cell decreases migration speed from 1 towards 0.1 with a Hill response, with half-max 0.1 and Hill power 4.
- oxygen decreases transform to M2 macrophage from 0.01 towards 0 with a Hill response, with half-max 5 and Hill power 4.
- dead increases debris secretion from 0 towards 0.017 with a Hill response, with half-max 0.1 and Hill power 10.

Cell type: M2 macrophage

BASE BEHAVIORS (AND PARAMETER VALUES)

Cycling: No.

Apoptosis: 7.20×10^{-5} min^{-1}.

Necrosis: No.

Motility: 1.0 micron/min, with persistence time 5 min, and migration bias 0.25 towards debris gradient.

Secretion: 10 min^{-1} of oxygen uptake, 1 min^{-1} of debris uptake, 1 min^{-1} of IFN-gamma uptake, and 10 min^{-1} of IL-10 secretion.

Phagocytosis: 0.1 min^{-1} for apoptotic cells and 0.017 min^{-1} for necrotic cells.

RULES

- contact with dead cell decreases migration speed from 1 towards 0.1 with a Hill response, with half-max 0.1 and Hill power 4.
- dead increases debris secretion from 0 towards 0.017 with a Hill response, with half-max 0.1 and Hill power 10.

Cell type: naive T cell

Cycling: 7.20×10^{-5} min^{-1}.

Apoptosis: 7.20×10^{-5} min^{-1}.

Necrosis: No.

Motility: 1.0 micron/min, with persistence time 5 min, and migration bias 0.5 towards IFN-gamma gradient.

Secretion: 10 min^{-1} of oxygen uptake, 1 min^{-1} of IFN-gamma uptake, and 1 min^{-1} of IL-10 uptake.

RULES

- IL-10 decreases transform to CD8 T cell from 0.001 towards 0 with a Hill response, with half-max 0.25 and Hill power 2.
- IFN-gamma increases transform to CD8 T cell from 0.001 towards 0.01 with a Hill response, with half-max 0.25 and Hill power 2.
- dead increases debris secretion from 0 towards 0.017 with a Hill response, with half-max 0.1 and Hill power 10.

Cell type: CD8 T cell

BASE BEHAVIORS (AND PARAMETER VALUES)

Cycling: 7.20×10^{-5} min^{-1}.

Apoptosis: 7.20×10^{-5} min^{-1}.

Necrosis: No.

Motility: 1.0 micron/min, with persistence time 5 min, and migration bias 0.5 towards IFN-gamma gradient.

Secretion: 10 min^{-1} of oxygen uptake, 1 min^{-1} of IFN-gamma uptake, and 1 min^{-1} of IL-10 uptake.

Attack: 0.1 min^{-1} attack rate to tumor cells, with 15 min of attack duration, and 1 min^{-1} of damage rate.

RULES

- IL-10 decreases attack tumor from 0.1 towards 0 with a Hill response, with half-max 0.25 and Hill power 2.
- IL-10 decreases migration speed from 1 towards 0.1 with a Hill response, with half-max 0.25 and Hill power 2.
- contact with tumor decreases migration speed from 1 towards 0.1 with a Hill response, with half-max 0.1 and Hill power 2.
- IFN-gamma increases cycle entry from 7.2e-05 towards 0.00041 with a Hill response, with half-max 0.25 and Hill power 2.
- IL-10 increases transform to exhausted T cell from 0 towards 0.005 with a Hill response, with half-max 0.25 and Hill power 4.
- dead increases debris secretion from 0 towards 0.017 with a Hill response, with half-max 0.1 and Hill power 10.

Cell type: exhausted T cell

BASE BEHAVIORS (AND PARAMETER VALUES)

Cycling: No.

Apoptosis: 7.20×10^{-5} min^{-1}.

Necrosis: No.

Motility: 0.01 micron/min, with persistence time 5 min, and migration bias 0.1 towards IFN-gamma gradient.

Secretion: 1 min^{-1} of oxygen uptake, 1 min^{-1} of IFN-gamma uptake, and 1 min^{-1} of IL-10 uptake.

RULES

- dead increases debris secretion from 0 towards 0.017 with a Hill response, with half-max 0.1 and Hill power 10.

Other implementation details

COMPUTATIONAL DOMAIN

The model simulates a 3D computational domain of $1500\ \mu m\ \times\ 1500\ \mu m\ \times\ 20\ \mu m$.

INITIALIZATION

The simulation begins with 2,000 viable tumor cells randomly seeded within a virtual disk of 372 microns in radius. Additionally, 400 M0 macrophages and 400 naive T cells are randomly seeded within an annulus with an inner radius of 450 microns and an outer radius of 500 microns, relative to the center of the domain.

MICROENVIRONMENT

The domain is initialized with a uniform oxygen partial pressure of 38 mmHg, maintained as a constant source at the boundaries. No initial concentration of debris, IFN-gamma, or IL-10 are considered, and cells act as sources and sinks of these substrates according to their phenotype states defined by the base behavior and rules.

Tumor-associated macrophage and EGFR signaling

This model is called "tam_egf" in https://github.com/PhysiCell-Models/grammar_samples

Cell Types and Rules

Cell type: tumor

BASE BEHAVIORS (AND PARAMETER VALUES)

Cycling: 1.0×10^{-4} min^{-1}.

Apoptosis: 7.2×10^{-5} min^{-1}.

Necrosis: 2.8×10^{-3} min^{-1}.

Motility: 0.17 micron/min, with persistence time 5 min, and no chemotaxis.

Secretion: 10 min^{-1} of oxygen uptake and 1 min^{-1} of EGF uptake.

RULES

- pressure decreases cycle entry from 0.0001 towards 0 with a Hill response, with half-max 1 and Hill power 4.
- oxygen decreases necrosis from 0.0028 towards 0 with a Hill response, with half-max 3.75 and Hill power 8.
- dead increases debris secretion from 0 towards 0.017 with a Hill response, with half-max 0.1 and Hill power 10. Rule applies to dead cells.

- IFN-gamma decreases migration speed from 0.17 towards 0 with a Hill response, with half-max 0.25 and Hill power 2.

GROW HYPOTHESIS:

- EGF increases cycle entry from 0.0001 towards 0.001 with a Hill response, with half-max 2 and Hill power 10.

GO HYPOTHESIS:

- EGF increases migration speed from 0.17 towards 0.5 with a Hill response, with half-max 2 and Hill power 10.
- EGF increases migration persistence time from 0.05 towards 10 with a Hill response, with half-max 2 and Hill power 10.
- EGF decreases cell-cell adhesion from 0.4 towards 0.2 with a Hill response, with half-max 2 and Hill power 10.

Cell type: M0 macrophage

BASE BEHAVIORS (AND PARAMETER VALUES)

Cycling: 7.2×10^{-5} min^{-1}.

Apoptosis: 7.2×10^{-5} min^{-1}.

Necrosis: No.

Motility: 0.5 micron/min, with persistence time 5 min, and migration bias 0.25 towards debris gradient.

Secretion: 5 min^{-1} of oxygen uptake and 1 min^{-1} of debris uptake.

Mechanics: No adhesion strength with other cells.

Phagocytosis: 0.0 min^{-1} for apoptotic cells and 0.0 min^{-1} for necrotic cells.

RULES

- contact with dead cell increases transform to M1 macrophage from 0 towards 0.1 with a Hill response, with half-max 0.1 and Hill power 10.
- debris increases transform to M1 macrophage from 0 towards 0.1 with a Hill response, with half-max 0.1 and Hill power 10.
- contact with dead cell decreases migration speed from 0.5 towards 0 with a Hill response, with half-max 0.01 and Hill power 4.
- dead increases debris secretion from 0 towards 0.017 with a Hill response, with half-max 0.1 and Hill power 10. Rule applies to dead cells.

Cell type: M1 macrophage

BASE BEHAVIORS (AND PARAMETER VALUES)

Cycling: 7.2×10^{-5} min^{-1}.

Apoptosis: 7.2×10^{-5} min^{-1}.

Necrosis: No.

Motility: 0.5 micron/min, with persistence time 5 min, and migration bias 0.25 towards debris gradient.

Secretion: 5 min^{-1} of oxygen uptake, 1 min^{-1} of debris uptake, 1 min^{-1} of IFN-gamma uptake, 10 min^{-1} of IFN-gamma secretion, and 1 min^{-1} of IL-4 uptake.

Mechanics: No adhesion strength with other cells.

Phagocytosis: 0.0 min^{-1} for apoptotic cells and 0.0 min^{-1} for necrotic cells.

Transition to: M2 macrophage with a rate of 0.01 min^{-1}.

RULES

- contact with dead cell decreases migration speed from 0.5 towards 0 with a Hill response, with half-max 0.01 and Hill power 4.
- oxygen decreases transform to M2 macrophage from 0.01 towards 0 with a Hill response, with half-max 5 and Hill power 4.
- IL-4 increases transform to M2 macrophage from 0.01 towards 1 with a Hill response, with half-max 1 and Hill power 4.
- IFN-gamma increases cycle entry from 7.2e-05 towards 0.00036 with a Hill response, with half-max 0.25 and Hill power 2.
- IFN-gamma increases phagocytose apoptotic cell from 0.0 towards 0.1 with a Hill response, with half-max 0.25 and Hill power 2.
- IFN-gamma increases phagocytose necrotic cell from 0.0 towards 0.017 with a Hill response, with half-max 0.25 and Hill power 2.
- dead increases debris secretion from 0 towards 0.017 with a Hill response, with half-max 0.1 and Hill power 10. Rule applies to dead cells.

Cell type: M2 macrophage

BASE BEHAVIORS (AND PARAMETER VALUES)

Cycling: 7.2×10^{-5} min^{-1}.

Apoptosis: 7.2×10^{-5} min^{-1}.

Necrosis: No.

Motility: 0.5 micron/min, with persistence time 5 min, and migration bias 0.25 towards debris gradient.

Secretion: 5 min^{-1} of oxygen uptake, 1 min^{-1} of debris uptake, 5 min^{-1} of IFN-gamma uptake, 10 min^{-1} of IL-10 secretion, 1 min^{-1} of IL-4 uptake, and 10 min^{-1} of EGF secretion.

Mechanics: No adhesion strength with other cells.

Phagocytosis: 0.0 min^{-1} for apoptotic cells and 0.0 min^{-1} for necrotic cells.

RULES

- IFN-gamma increases transform to M1 macrophage from 0 towards 0.001 with a Hill response, with half-max 1 and Hill power 4.
- contact with dead cell decreases migration speed from 0.5 towards 0 with a Hill response, with half-max 0.01 and Hill power 4.
- IFN-gamma decreases cycle entry from 7.2e-05 towards 0 with a Hill response, with half-max 0.25 and Hill power 2.
- IL-4 increases cycle entry from 7.2e-05 towards 0.001 with a Hill response, with half-max 0.25 and Hill power 2.
- IFN-gamma increases phagocytose apoptotic cell from 0.0 towards 0.1 with a Hill response, with half-max 0.25 and Hill power 2.
- IFN-gamma increases phagocytose necrotic cell from 0.0 towards 0.017 with a Hill response, with half-max 0.25 and Hill power 2.
- dead increases debris secretion from 0 towards 0.017 with a Hill response, with half-max 0.1 and Hill power 10. Rule applies to dead cells.

Cell type: Th2 CD4 T cell

BASE BEHAVIORS (AND PARAMETER VALUES)

Cycling: No.

Apoptosis: 7.2×10^{-5} min^{-1}.

Necrosis: No.

Motility: 0.5 micron/min, with persistence time 5 min, and migration bias 0.5 towards debris gradient.

Secretion: 5 min^{-1} of oxygen uptake, 1 min^{-1} of IL-10 uptake, 1 min^{-1} of IL-4 uptake, and 5 min^{-1} of IL-4 secretion.

Mechanics: No adhesion strength with other cells.

RULES

- IL-4 increases cycle entry from 0 towards 0.00036 with a Hill response, with half-max 0.25 and Hill power 2.
- IFN-gamma decreases cycle entry from 0 towards 0 with a Hill response, with half-max 0.25 and Hill power 2.
- contact with M0 macrophage decreases migration speed from 0.5 towards 0.25 with a Hill response, with half-max 0.1 and Hill power 2.
- contact with M1 macrophage decreases migration speed from 0.5 towards 0.25 with a Hill response, with half-max 0.1 and Hill power 2.
- contact with M2 macrophage decreases migration speed from 0.5 towards 0.25 with a Hill response, with half-max 0.1 and Hill power 2.
- contact with tumor decreases migration speed from 0.5 towards 0.25 with a Hill response, with half-max 0.1 and Hill power 2.
- dead increases debris secretion from 0 towards 0.017 with a Hill response, with half-max 0.1 and Hill power 10. Rule applies to dead cells.

Other implementation details

COMPUTATIONAL DOMAIN

The model simulates a 3D computational domain of $2000\ \mu m\ \times\ 2000\ \mu m\ \times\ 20\ \mu m$.

INITIALIZATION

The simulation begins with 200 tumor cells randomly seeded within a virtual disk of 150 microns in radius. Additionally, 10 macrophages are randomly seeded within an annulus with an inner radius of 200 microns and an outer radius of 350 microns, relative to the center of the domain.

MICROENVIRONMENT

The domain is initialized with a uniform oxygen partial pressure of 20 mmHg, maintained as a constant source at the boundaries. No initial concentration of debris, IFN-gamma, IL-10, IL-4, or EGF are considered, and cells act as sources and sinks of these substrates according to their phenotype states defined by the base behavior and rules.

PDAC immunotherapy

This model is called “pdac_therapy” in https://github.com/PhysiCell-Models/grammar_samples

Cell Types and Rules

Cell type: PD-L1lo_tumor

BASE BEHAVIORS (AND PARAMETER VALUES)

Cycling: $7.2\ \times 10^{-4}$ min^{-1}.

Apoptosis: 7.2×10^{-5} min^{-1}.

Necrosis: No.

Motility: 0.5 micron/min, with persistence time 5 min, and no chemotaxis.

Secretion: 10 min^{-1} of oxygen uptake.

RULES

- pressure decreases cycle entry from 0.00072 towards 0 with a Hill response, with half-max 0.25 and Hill power 3.
- damage increases apoptosis from 7.2e-05 towards 0.023 with a Hill response, with half-max 45 and Hill power 16.

- dead increases debris secretion from 0 towards 0.017 with a Hill response, with half-max 0.1 and Hill power 10. Rule applies to dead cells.

Cell type: PD-L1hi_tumor

BASE BEHAVIORS (AND PARAMETER VALUES)

Cycling: 7.2×10^{-4} min^{-1}.

Apoptosis: 7.2×10^{-5} min^{-1}.

Necrosis: No.

Motility: 0.5 micron/min, with persistence time 5 min, and no chemotaxis.

Secretion: 10 min^{-1} of oxygen uptake and 1 min^{-1} of pro-inflammatory factor uptake

RULES

- pressure decreases cycle entry from 0.00072 towards 0 with a Hill response, with half-max 0.25 and Hill power 3.
- damage increases apoptosis from 7.2e-05 towards 0.023 with a Hill response, with half-max 45 and Hill power 16.
- dead increases debris secretion from 0 towards 0.017 with a Hill response, with half-max 0.1 and Hill power 10. Rule applies to dead cells.

Cell type: macrophage

BASE BEHAVIORS (AND PARAMETER VALUES)

Cycling: No.

Apoptosis: No.

Necrosis: No.

Motility: 1 micron/min, with persistence time 5 min, and migration bias 0.25 towards debris gradient.

Secretion: 10 min^{-1} of oxygen uptake, 1 min^{-1} of debris uptake, and 1 min^{-1} of anti-inflammatory factor secretion.

Mechanics: No adhesion strength with other cells.

Phagocytosis: 0.1 min^{-1} for apoptotic cells and 0.017 min^{-1} for necrotic cells.

RULES

- oxygen decreases anti-inflammatory factor secretion from 1 towards 0 with a Hill response, with half-max 5 and Hill power 4.
- oxygen increases pro-inflammatory factor secretion from 0 towards 0.1 with a Hill response, with half-max 5 and Hill power 4.

Cell type: PD-1hi_CD137lo_CD8_Tcell

BASE BEHAVIORS (AND PARAMETER VALUES)

Cycling: No.

Apoptosis: No.

Necrosis: No.

Motility: 1 micron/min, with persistence time 5 min, and migration bias 0.5 towards pro-inflammatory factor gradient.

Secretion: 10 min^{-1} of oxygen uptake.

Mechanics: No adhesion strength with other cells.

Attack: 3.33×10^{-6} min^{-1} attack rate to PD-L1lo_tumor cell, with 15 min of attack duration, and 1 min^{-1} of damage rate.

RULES

- contact with PD-L1hi_tumor decreases migration speed from 1 towards 0 with a Hill response, with half-max 0.1 and Hill power 2.
- contact with PD-L1lo_tumor decreases migration speed from 1 towards 0 with a Hill response, with half-max 0.1 and Hill power 2.
- anti-inflammatory factor decreases migration speed from 1 towards 0 with a Hill response, with half-max 2.5 and Hill power 2.
- anti-inflammatory factor decreases attack PD-L1hi_tumor from 0 towards 0 with a Hill response, with half-max 2.5 and Hill power 2.
- anti-inflammatory factor decreases attack PD-L1lo_tumor from 3.33e-06 towards 0 with a Hill response, with half-max 2.5 and Hill power 2.

Cell type: PD-1lo_CD137lo_CD8_Tcell

BASE BEHAVIORS (AND PARAMETER VALUES)

Cycling: No.

Apoptosis: No.

Necrosis: No.

Motility: 1 micron/min, with persistence time 5 min, and migration bias 0.5 towards pro-inflammatory factor gradient.

Secretion: 10 min^{-1} of oxygen uptake.

Mechanics: No adhesion strength with other cells.

Attack: 3.33×10^{-5} min^{-1} attack rate to PD-L1lo_tumor and PD-L1hi_tumor cells, with 15 min of attack duration, and 1 min^{-1} of damage rate.

RULES

- contact with PD-L1hi_tumor decreases migration speed from 1 towards 0 with a Hill response, with half-max 0.1 and Hill power 2.
- contact with PD-L1lo_tumor decreases migration speed from 1 towards 0 with a Hill response, with half-max 0.1 and Hill power 2.
- anti-inflammatory factor decreases migration speed from 1 towards 0 with a Hill response, with half-max 2.5 and Hill power 2.
- anti-inflammatory factor decreases attack PD-L1hi_tumor from 3.33e-05 towards 0 with a Hill response, with half-max 2.5 and Hill power 2.
- pro-inflammatory factor increases attack PD-L1hi_tumor from 3.33e-05 towards 0.01 with a Hill response, with half-max 0.1 and Hill power 2.
- anti-inflammatory factor decreases attack PD-L1lo_tumor from 3.33e-05 towards 0 with a Hill response, with half-max 2.5 and Hill power 2.
- pro-inflammatory factor increases attack PD-L1lo_tumor from 3.33e-05 towards 0.01 with a Hill response, with half-max 0.1 and Hill power 2.

Cell type: PD-1hi_CD137hi_CD8_Tcell

BASE BEHAVIORS (AND PARAMETER VALUES)

Cycling: No.

Apoptosis: No.

Necrosis: No.

Motility: 1 micron/min, with persistence time 5 min, and migration bias 0.5 towards pro-inflammatory factor gradient.

Secretion: 10 min^{-1} of oxygen uptake and 1 min^{-1} of pro-inflammatory factor secretion

Mechanics: No adhesion strength with other cells.

Attack: 3.33×10^{-5} min^{-1} attack rate to PD-L1lo_tumor cell, with 15 min of attack duration, and 1 min^{-1} of damage rate.

RULES

- contact with PD-L1hi_tumor decreases migration speed from 1 towards 0 with a Hill response, with half-max 0.1 and Hill power 2.
- contact with PD-L1lo_tumor decreases migration speed from 1 towards 0 with a Hill response, with half-max 0.1 and Hill power 2.
- anti-inflammatory factor decreases migration speed from 1 towards 0 with a Hill response, with half-max 2.5 and Hill power 2.
- anti-inflammatory factor decreases attack PD-L1hi_tumor from 0 towards 0 with a Hill response, with half-max 2.5 and Hill power 2.
- anti-inflammatory factor decreases attack PD-L1lo_tumor from 3.33e-05 towards 0 with a Hill response, with half-max 2.5 and Hill power 2.

Cell type: PD-1lo_CD137hi_CD8_Tcell

BASE BEHAVIORS (AND PARAMETER VALUES)

Cycling: No.

Apoptosis: No.

Necrosis: No.

Motility: 1 micron/min, with persistence time 5 min, and migration bias 0.5 towards pro-inflammatory factor gradient.

Secretion: 10 min^{-1} of oxygen uptake and 0.1 min^{-1} of pro-inflammatory factor secretion

Mechanics: No adhesion strength with other cells.

Attack: 3.33×10^{-4} min^{-1} attack rate to PD-L1lo_tumor and PD-L1hi_tumor cells, with 15 min of attack duration, and 1 min^{-1} of damage rate.

RULES

- contact with PD-L1hi_tumor decreases migration speed from 1 towards 0 with a Hill response, with half-max 0.1 and Hill power 2.
- contact with PD-L1lo_tumor decreases migration speed from 1 towards 0 with a Hill response, with half-max 0.1 and Hill power 2.
- anti-inflammatory factor decreases migration speed from 1 towards 0 with a Hill response, with half-max 2.5 and Hill power 2.
- anti-inflammatory factor decreases attack PD-L1hi_tumor from 0.000333 towards 0 with a Hill response, with half-max 2.5 and Hill power 2.
- pro-inflammatory factor increases attack PD-L1hi_tumor from 0.000333 towards 0.1 with a Hill response, with half-max 0.1 and Hill power 2.
- anti-inflammatory factor decreases attack PD-L1lo_tumor from 0.000333 towards 0 with a Hill response, with half-max 2.5 and Hill power 2.
- pro-inflammatory factor increases attack PD-L1lo_tumor from 0.000333 towards 0.1 with a Hill response, with half-max 0.1 and Hill power 2.

Cell type: PD-1hi_CD4_Tcell

BASE BEHAVIORS (AND PARAMETER VALUES)

Cycling: No.

Apoptosis: No.

Necrosis: No.

Motility: 1 micron/min, with persistence time 5 min, and migration bias 0.25 towards pro-inflammatory factor gradient.

Secretion: 10 min^{-1} of oxygen uptake.

Mechanics: No adhesion strength with other cells.

RULES

- anti-inflammatory factor decreases migration speed from 1 towards 0 with a Hill response, with half-max 2.5 and Hill power 2.
- contact with macrophage decreases migration speed from 1 towards 0 with a Hill response, with half-max 0.1 and Hill power 2.

Cell type: PD-1lo_CD4_Tcell

BASE BEHAVIORS (AND PARAMETER VALUES)

Cycling: No.

Apoptosis: No.

Necrosis: No.

Motility: 1 micron/min, with persistence time 5 min, and migration bias 0.25 towards pro-inflammatory factor gradient.

Secretion: 10 min^{-1} of oxygen uptake and 0.1 min^{-1} of pro-inflammatory factor secretion.

Mechanics: No adhesion strength with other cells.

RULES

- anti-inflammatory factor decreases migration speed from 1 towards 0 with a Hill response, with half-max 2.5 and Hill power 2.
- contact with macrophage decreases migration speed from 1 towards 0 with a Hill response, with half-max 0.1 and Hill power 2.

Other implementation details

COMPUTATIONAL DOMAIN

The model simulates a 3D computational domain of $2000\ \mu m\ \times\ 2000\ \mu m\ \times\ 20\ \mu m$.

INITIALIZATION

The simulation begins with data-derived cell counts. See the "ic_cells" folder for the initialization states. The three considered immunotherapies affect the initialization by shifting the cellular composition of the microenvironment. GVAX doubles the T cell populations. ICI shifts all T cells in a PD-1^{hi} state to a PD-1^{lo} state. URU shifts all CD8+ T cells in a $CD137^{lo}$ state to a $CD137^{hi}$ state.

MICROENVIRONMENT

The domain is initialized with a uniform oxygen partial pressure of 38 mmHg, maintained as a constant source at the boundaries. No initial concentration of debris, pro-inflammatory factor, or anti-inflammatory

factor are considered, and cells act as sources and sinks of these substrates according to their phenotype states defined by the base behavior and rules.

Brain development

This model is called "neuro_dev" in https://github.com/PhysiCell-Models/grammar_samples .

AUD - Cell Types and Rules

Cell type: apical

BASE BEHAVIORS (AND PARAMETER VALUES)

Cycling: No.

Apoptosis: No.

Necrosis: No.

Motility: 1 micron/min, with persistence time 5 min, and migration bias 0.5 towards rgc_taxis_factor gradient.

Secretion: 1 min^{-1} of rgc_taxis_factor secretion.

RULES

No rules.

Cell type: pial

BASE BEHAVIORS (AND PARAMETER VALUES)

Cycling: No.

Apoptosis: No.

Necrosis: No.

Motility: No.

Secretion: 10 min^{-1} of reelin secretion.

Mechanics: 1 micron/min of cell-cell adhesion strength with all cells. Attachment rate of 0.1 min^{-1}, with elastic constant of 0.01 min^{-1}, and affinity with all cells except apical cells.

RULES

No rules.

Cell type: rgc

BASE BEHAVIORS (AND PARAMETER VALUES)

Cycling: 1.30×10^{-3} min^{-1} (live model).

Apoptosis: No.

Necrosis: No.

Motility: 1 micron/min, with persistence time 1 min, and migration bias 0.5 towards rgc_taxis_factor gradient.

Secretion: No.

RULES

- contact with apical decreases migration speed from 1 towards 0 with a Hill response, with half-max 0.5 and Hill power 8.

- time decreases cycle entry from 0.0013 towards 0.00085 with a Hill response, with half-max 4808 and Hill power 10.
- time increases asymmetric division to layer_6 from 0 towards 1 with a Hill response, with half-max 1440 and Hill power 256.
- time decreases asymmetric division to layer_6 from 0 towards 0 with a Hill response, with half-max 3309 and Hill power 256.
- time increases asymmetric division to layer_5 from 0 towards 1 with a Hill response, with half-max 3309 and Hill power 256.
- time decreases asymmetric division to layer_5 from 0 towards 0 with a Hill response, with half-max 7068 and Hill power 256.
- time increases asymmetric division to layer_4 from 0 towards 1 with a Hill response, with half-max 7068 and Hill power 256.
- time decreases asymmetric division to layer_4 from 0 towards 0 with a Hill response, with half-max 8638 and Hill power 256.
- time increases asymmetric division to layer_3 from 0 towards 1 with a Hill response, with half-max 8638 and Hill power 256.
- time decreases asymmetric division to layer_3 from 0 towards 0 with a Hill response, with half-max 10799 and Hill power 256.
- time increases asymmetric division to layer_2 from 0 towards 1 with a Hill response, with half-max 10799 and Hill power 256.
- time decreases asymmetric division to layer_2 from 0 towards 0 with a Hill response, with half-max 12960 and Hill power 256.
- time increases apoptosis from 0 towards 1 with a Hill response, with half-max 12960 and Hill power 256.

Cell type: layer_6

BASE BEHAVIORS (AND PARAMETER VALUES)

Cycling: No.

Apoptosis: No.

Necrosis: No.

Motility: 2 micron/min, with persistence time 1 min, and migration bias 0.5 towards reelin gradient.

Secretion: No.

Mechanics: No adhesion strength with other cells.

RULES

No rules.

Cell type: layer_5

BASE BEHAVIORS (AND PARAMETER VALUES)

Cycling: No.

Apoptosis: No.

Necrosis: No.

Motility: 2 micron/min, with persistence time 1 min, and migration bias 0.5 towards reelin gradient.

Secretion: No.

Mechanics: No adhesion strength with other cells.

RULES

No rules.

Cell type: layer_4

BASE BEHAVIORS (AND PARAMETER VALUES)

Cycling: No.

Apoptosis: No.

Necrosis: No.

Motility: 2 micron/min, with persistence time 1 min, and migration bias 0.5 towards reelin gradient.

Secretion: No.

Mechanics: No adhesion strength with other cells.

RULES

No rules.

Cell type: layer_3

BASE BEHAVIORS (AND PARAMETER VALUES)

Cycling: No.

Apoptosis: No.

Necrosis: No.

Motility: 2 micron/min, with persistence time 1 min, and migration bias 0.5 towards reelin gradient.

Secretion: No.

Mechanics: No adhesion strength with other cells.

RULES

No rules.

Cell type: layer_2

BASE BEHAVIORS (AND PARAMETER VALUES)

Cycling: No.

Apoptosis: No.

Necrosis: No.

Motility: 2 micron/min, with persistence time 1 min, and migration bias 0.5 towards reelin gradient.

Secretion: No.

Mechanics: No adhesion strength with other cells.

RULES

No rules.

SOM - Cell Types and Rules

All cells and base behaviors are considered equal to AUD, except as defined by the rules governing rgc cells.

RGC RULES

- contact with apical decreases migration speed from 1 towards 0 with a Hill response, with half-max 0.5 and Hill power 8.

- time decreases cycle entry from 0.0013 towards 0.00085 with a Hill response, with half-max 9206 and Hill power 10.
- time increases asymmetric division to layer_6 from 0 towards 1 with a Hill response, with half-max 1440 and Hill power 256.
- time decreases asymmetric division to layer_6 from 0 towards 0 with a Hill response, with half-max 5007 and Hill power 256.
- time increases asymmetric division to layer_5 from 0 towards 1 with a Hill response, with half-max 5007 and Hill power 256.
- time decreases asymmetric division to layer_5 from 0 towards 0 with a Hill response, with half-max 6554 and Hill power 256.
- time increases asymmetric division to layer_4 from 0 towards 1 with a Hill response, with half-max 6554 and Hill power 256.
- time decreases asymmetric division to layer_4 from 0 towards 0 with a Hill response, with half-max 9010 and Hill power 256.
- time increases asymmetric division to layer_3 from 0 towards 1 with a Hill response, with half-max 9010 and Hill power 256.
- time decreases asymmetric division to layer_3 from 0 towards 0 with a Hill response, with half-max 10985 and Hill power 256.
- time increases asymmetric division to layer_2 from 0 towards 1 with a Hill response, with half-max 10985 and Hill power 256.
- time decreases asymmetric division to layer_2 from 0 towards 0 with a Hill response, with half-max 12960 and Hill power 256.
- time increases apoptosis from 0 towards 1 with a Hill response, with half-max 12960 and Hill power 256.

Other implementation details

COMPUTATIONAL DOMAIN

The model simulates a 3D computational domain of $410\ \mu m \times 1300\ \mu m \times 20\ \mu m$.

INITIALIZATION

The simulation begins with a layer of apical cells on the bottom boundary, a layer of rgc cells on top of them, and then a layer of pial cells above those. This information is stored within the "cells.csv" file for the project.

MICROENVIRONMENT

The domain is initialized with no concentration of reelin or rgc_taxis_factor, and cells act as sources and sinks of these substrates according to their phenotype states defined by the base behavior and rules.

Calibration

To calibrate the model, we derived quantitative metrics from a columnar subset of the Allen Brain Atlas's MERFISH spatial transcriptomic data. The specific metrics chosen were the neuron counts of each cortical layer (VI, V, IV, and II/III), as well as the total neuron count of the entire column.

We selected the half-maxes of the rules affecting rgc cycling rate and asymmetric division probabilities for calibration. These control how quickly the rgc proliferation slows as well as the timing of the switch to different layers produced by asymmetric division. We fixed the total length of the neurogenic phase to 8 days based on a consensus from literature.

We employed the Nelder-Mead algorithm from scipy.optimize to fit these parameters for each region using the neuron counts from the column in that region, minimizing the residual sum of squares of the thickness of the layers. The scripts for performing this on a high performance computing (HPC) cluster are provided in the GitHub repository.

ADDITIONAL RESULTS FOR THE VIRTUAL CLINICAL TRIAL EXAMPLE

We performed additional analyses on tumor shrinkage (**Fig. S19**), and immune cell population abundances during GVAX + ICI + URU treatment (**Fig. S20**), GVAX treatment (**Fig. S21**), ICI treatment (**Fig. S22**) GVAX + ICI treatment (**Fig. S23**), URU treatment (**Fig. S24**), GVAX + URU treatment (**Fig. S25**), and ICI + URU treatment (**Fig. S26**). These results can be found in **Figs. S19-S26**.
