# Supplementary material for: Human interpretable grammar encodes multicellular systems biology models to democratize virtual cell laboratories
Source: Cell. Author manuscript; Available in PMC 2026 Mar 24. (PMC13012569; doi:10.1016/j.cell.2025.06.048)
Supplement: 15 [file NIHMS2100468-supplement-15.pdf]

## Supplemental References


1. Sluka, J.P., Shirinifard, A., Swat, M., Cosmanescu, A., Heiland, R.W., and Glazier, J.A. (2014). The cell behavior ontology: describing the intrinsic biological behaviors of real and model cells seen as active agents. Bioinformatics *30*, 2367-2374. 10.1093/bioinformatics/btu210.
2. Friedman, S.H., Anderson, A.R.A., Bortz, D.M., Fletcher, A.G., Frieboes, H.B., Ghaffarizadeh, A., Grimes, D.R., Hawkins-Daarud, A., Hoehme, S., Juarez, E.F., et al. (2016). MultiCellDS: a community-developed standard for curating microenvironment-dependent multicellular data. bioRxiv **[preprint]** *090456*. 10.1101/090456.
3. Sundus, A., Kurtoglu, F., Konstantinopoulos, K., Chen, M., Willis, D., Heiland, R., and Macklin, P. (2022). PhysiCell training apps: Cloud hosted open-source apps to learn cell-based simulation software. bioRxiv **[preprint]** *10.1101/2022.06.24.497566*. 10.1101/2022.06.24.497566.
4. Jenner, A.L., Smalley, M., Goldman, D., Goins, W.F., Cobbs, C.S., Puchalski, R.B., Chiocca, E.A., Lawler, S., Macklin, P., Goldman, A., and Craig, M. (2022). Agent-based computational modeling of glioblastoma predicts that stromal density is central to oncolytic virus efficacy. iScience *25*, 104395. 10.1016/j.isci.2022.104395.
5. Islam, M.A., Getz, M., Macklin, P., and Versypt, A.N.F. (2022). An agent-based modeling approach for lung fibrosis in response to COVID-19. bioRxiv **[preprint]**. 10.1101/2022.10.03.510677.
6. Wang, Y., Brodin, E., Nishii, K., Frieboes, H.B., Mumenthaler, S.M., Sparks, J.L., and Macklin, P. (2021). Impact of tumor-parenchyma biomechanics on liver metastatic progression: a multi-model approach. Sci Rep *11*, 1710. 10.1038/s41598-020-78780-7.
7. Rocha, H.L., Godet, I., Kurtoglu, F., Metzcar, J., Konstantinopoulos, K., Bhoyar, S., Gilkes, D.M., and Macklin, P. (2021). A persistent invasive phenotype in post-hypoxic tumor cells is revealed by fate mapping and computational modeling. iScience *24*, 102935. 10.1016/j.isci.2021.102935.
8. Getz, M., Wang, Y., An, G., Asthana, M., Becker, A., Cockrell, C., Collier, N., Craig, M., Davis, C.L., Faeder, J.R., et al. (2021). Iterative community-driven development of a SARS-CoV-2 tissue simulator. bioRxiv **[preprint]**. 10.1101/2020.04.02.019075.
9. Risner, K.H., Tieu, K.V., Wang, Y., Bakovic, A., Alem, F., Bhalla, N., Nathan, S., Conway, D.E., Macklin, P., and Narayanan, A. (2020). Maraviroc inhibits SARS-CoV-2 multiplication and s-protein mediated cell fusion in cell culture. bioRxiv **[preprint]**. 10.1101/2020.08.12.246389.
10. Ozik, J., Collier, N., Heiland, R., An, G., and Macklin, P. (2019). Learning-accelerated discovery of immune-tumour interactions. Mol Syst Des Eng *4*, 747-760. 10.1039/c9me00036d.
11. Letort, G., Montagud, A., Stoll, G., Heiland, R., Barillot, E., Macklin, P., Zinovyev, A., and Calzone, L. (2019). PhysiBoSS: a multi-scale agent-based modelling framework integrating physical dimension and cell signalling. Bioinformatics *35*, 1188-1196. 10.1093/bioinformatics/bty766.
12. Ozik, J., Collier, N., Wozniak, J.M., Macal, C., Cockrell, C., Friedman, S.H., Ghaffarizadeh, A., Heiland, R., An, G., and Macklin, P. (2018). High-throughput cancer hypothesis testing with an integrated PhysiCell-EMEWS workflow. BMC Bioinformatics *19*, 483. 10.1186/s12859-018-2510-x.
13. Ghaffarizadeh, A., Heiland, R., Friedman, S.H., Mumenthaler, S.M., and Macklin, P. (2018). PhysiCell: An open source physics-based cell simulator for 3-D multicellular systems. PLoS Comput Biol *14*, e1005991. 10.1371/journal.pcbi.1005991.
14. Islam, M.A., Getz, M., Macklin, P., and Versypt, A.N.F. (2023). An agent-based modeling approach for lung fibrosis in response to COVID-19. PLoS Comput Biol *19*, e1011741. 10.1371/journal.pcbi.1011741.
15. Ghaffarizadeh, A., Heiland, R., Friedman, S.H., Mumenthaler, S.M., and Macklin, P. (2018). PhysiCell: An open source physics-based cell simulator for 3-D multicellular systems. PLOS Computational Biology *14*, e1005991. 10.1371/journal.pcbi.1005991.
16. Rocha, H.L., Aguilar, B., Getz, M., Shmulevich, I., and Macklin, P. (2024). A multiscale model of immune surveillance in micrometastases gives insights on cancer patient digital twins. npj Syst Biol Appl *10*. 10.1038/s41540-024-00472-z.
17. Metzcar, J., Duggan, B.S., Fischer, B., Murphy, M., Heiland, R., and Macklin, P. (2025). A Simple Framework for Agent-Based Modeling with Extracellular Matrix. Bull Math Biol *87*, 43. 10.1007/s11538-024-01408-8.

18. Juarez, E.F., Lau, R., Friedman, S.H., Ghaffarizadeh, A., Jonckheere, E., Agus, D.B., Mumenthaler, S.M., and Macklin, P. (2016). Quantifying differences in cell line population dynamics using CellPD. BMC Systems Biology *10*. 10.1186/s12918-016-0337-5.
19. Sadlonova, A., Novak, Z., Johnson, M.R., Bowe, D.B., Gault, S.R., Page, G.P., Thottassery, J.V., Welch, D.R., and Frost, A.R. (2005). Breast fibroblasts modulate epithelial cell proliferation in three-dimensional in vitro co-culture. Breast Cancer Res *7*, R46-59. 10.1186/bcr949.
20. Cooper, G.M. (2000). The Eukaryotic Cell Cycle. . https://www.ncbi.nlm.nih.gov/books/NBK9876/.
21. Macklin, P., Edgerton, M.E., Thompson, A.M., and Cristini, V. (2012). Patient-calibrated agent-based modelling of ductal carcinoma in situ (DCIS): from microscopic measurements to macroscopic predictions of clinical progression. J Theor Biol *301*, 122-140. 10.1016/j.jtbi.2012.02.002.
22. Macklin, P., Mumenthaler, S., and Lowengrub, J. (2013). Modeling Multiscale Necrotic and Calcified Tissue Biomechanics in Cancer Patients: Application to Ductal Carcinoma In Situ (DCIS). In Multiscale Computer Modeling in Biomechanics and Biomedical Engineering, pp. 349-380. 10.1007/8415_2012_150.
23. Majno, G., and Joris, I. (1995). Apoptosis, oncosis, and necrosis. An overview of cell death. Am J Pathol *146*, 3-15.
24. Krysko, D.V., Vanden Berghe, T., D'Herde, K., and Vandenabeele, P. (2008). Apoptosis and necrosis: detection, discrimination and phagocytosis. Methods *44*, 205-221. 10.1016/j.ymeth.2007.12.001.
25. Kerr, J.F., Winterford, C.M., and Harmon, B.V. (1994). Apoptosis. Its significance in cancer and cancer therapy. Cancer *73*, 2013-2026. 10.1002/1097-0142(19940415)73:8<2013::aid-cncr2820730802>3.0.co;2-j.
26. Hengartner, M.O. (2000). The biochemistry of apoptosis. Nature *407*, 770-776. 10.1038/35037710.
27. Garland, J.M., and Halestrap, A. (1997). Energy metabolism during apoptosis. Bcl-2 promotes survival in hematopoietic cells induced to apoptose by growth factor withdrawal by stabilizing a form of metabolic arrest. J Biol Chem *272*, 4680-4688. 10.1074/jbc.272.8.4680.
28. Hyun, A.Z., and Macklin, P. (2013). Improved patient-specific calibration for agent-based cancer modeling. J Theor Biol *317*, 422-424. 10.1016/j.jtbi.2012.10.017.
29. Ghaffarizadeh, A., Friedman, S.H., and Macklin, P. (2016). BioFVM: an efficient, parallelized diffusive transport solver for 3-D biological simulations. Bioinformatics *32*, 1256-1258. 10.1093/bioinformatics/btv730.
30. Wang, J., Delfarah, A., Gelbach, P.E., Fong, E., Macklin, P., Mumenthaler, S.M., Graham, N.A., and Finley, S.D. (2022). Elucidating tumor-stromal metabolic crosstalk in colorectal cancer through integration of constraint-based models and LC-MS metabolomics. Metab Eng *69*, 175-187. 10.1016/j.ymben.2021.11.006.
31. Tavakoli, N., Fong, E.J., Coleman, A., Huang, Y.K., Bigger, M., Doche, M.E., Kim, S., Lenz, H.J., Graham, N.A., Macklin, P., et al. (2025). Merging Metabolic Modeling and Imaging for Screening Therapeutic Targets in Colorectal Cancer. npj Syst Biol Appl *11*, 12. 10.1038/s41540-025-00494-1.
32. Theveneau, E., Steventon, B., Scarpa, E., Garcia, S., Trepat, X., Streit, A., and Mayor, R. (2013). Chase-and-run between adjacent cell populations promotes directional collective migration. Nat Cell Biol *15*, 763-772. 10.1038/ncb2772.
33. Ray, A., Slama, Z.M., Morford, R.K., Madden, S.A., and Provenzano, P.P. (2017). Enhanced Directional Migration of Cancer Stem Cells in 3D Aligned Collagen Matrices. Biophys J *112*, 1023-1036. 10.1016/j.bpj.2017.01.007.
34. Liu, Z., Lee, S.J., Park, S., Konstantopoulos, K., Glunde, K., Chen, Y., and Barman, I. (2020). Cancer cells display increased migration and deformability in pace with metastatic progression. FASEB J *34*, 9307-9315. 10.1096/fj.202000101RR.
35. Friedl, P., Zanker, K.S., and Brocker, E.B. (1998). Cell migration strategies in 3-D extracellular matrix: differences in morphology, cell matrix interactions, and integrin function. Microsc Res Tech *43*, 369-378. 10.1002/(SICI)1097-0029(19981201)43:5<369::AID-JEMT3>3.0.CO;2-6.
36. Clark, A.G., and Vignjevic, D.M. (2015). Modes of cancer cell invasion and the role of the microenvironment. Curr Opin Cell Biol *36*, 13-22. 10.1016/j.ceb.2015.06.004.
37. Tschumperlin, D.J. (2013). Fibroblasts and the ground they walk on. Physiology (Bethesda) *28*, 380-390. 10.1152/physiol.00024.2013.

38. Hakkinen, K.M., Harunaga, J.S., Doyle, A.D., and Yamada, K.M. (2011). Direct comparisons of the morphology, migration, cell adhesions, and actin cytoskeleton of fibroblasts in four different three-dimensional extracellular matrices. Tissue Eng Part A *17*, 713-724. 10.1089/ten.TEA.2010.0273.
39. Pixley, F.J. (2012). Macrophage Migration and Its Regulation by CSF-1. Int J Cell Biol *2012*, 501962. 10.1155/2012/501962.
40. Lefort, C.T., and Kim, M. (2010). Human T lymphocyte isolation, culture and analysis of migration in vitro. J Vis Exp. 10.3791/2017.
41. Miller, M.J., Wei, S.H., Cahalan, M.D., and Parker, I. (2003). Autonomous T cell trafficking examined in vivo with intravital two-photon microscopy. Proc Natl Acad Sci U S A *100*, 2604-2609. 10.1073/pnas.2628040100.
42. Glen, C.M., Kemp, M.L., and Voit, E.O. (2019). Agent-based modeling of morphogenetic systems: Advantages and challenges. PLoS Comput Biol *15*, e1006577. 10.1371/journal.pcbi.1006577.
43. Maiuri, P., Rupprecht, J.F., Wieser, S., Ruprecht, V., Benichou, O., Carpi, N., Coppey, M., De Beco, S., Gov, N., Heisenberg, C.P., et al. (2015). Actin flows mediate a universal coupling between cell speed and cell persistence. Cell *161*, 374-386. 10.1016/j.cell.2015.01.056.
44. Mirams, G.R., Arthurs, C.J., Bernabeu, M.O., Bordas, R., Cooper, J., Corrias, A., Davit, Y., Dunn, S.J., Fletcher, A.G., Harvey, D.G., et al. (2013). Chaste: an open source C++ library for computational physiology and biology. PLoS Comput Biol *9*, e1002970. 10.1371/journal.pcbi.1002970.
45. Kang, S., Kahan, S., McDermott, J., Flann, N., and Shmulevich, I. (2014). Biocellion: accelerating computer simulation of multicellular biological system models. Bioinformatics *30*, 3101-3108. 10.1093/bioinformatics/btu498.
46. Hoehme, S., and Drasdo, D. (2010). A cell-based simulation software for multi-cellular systems. Bioinformatics *26*, 2641-2642. 10.1093/bioinformatics/btq437.
47. Abbasi, A., Amjad-Iranagh, S., and Dabir, B. (2022). CellSys: An open-source tool for building initial structures for bio-membranes and drug-delivery systems. J Comput Chem *43*, 331-339. 10.1002/jcc.26793.
48. Cytowski, M., Szymańska, Z., Umiński, P., Andrejczuk, G., and Raszkowski, K. (2017). Implementation of an Agent-Based Parallel Tissue Modelling Framework for the Intel MIC Architecture. Scientific Programming *2017*, 1-11. 10.1155/2017/8721612.
49. Mathias, S., Coulier, A., Bouchnita, A., and Hellander, A. (2020). Impact of Force Function Formulations on the Numerical Simulation of Centre-Based Models. Bulletin of Mathematical Biology *82*. 10.1007/s11538-020-00810-2.
50. van Leeuwen, I.M.M., Mirams, G.R., Walter, A., Fletcher, A., Murray, P., Osborne, J., Varma, S., Young, S.J., Cooper, J., Doyle, B., et al. (2009). An integrative computational model for intestinal tissue renewal. Cell Proliferation *42*, 617-636. 10.1111/j.1365-2184.2009.00627.x.
51. Meineke, F.A., Potten, C.S., and Loeffler, M. (2001). Cell migration and organization in the intestinal crypt using a lattice-free model. Cell Proliferation *34*, 253-266. 10.1046/j.0960-7722.2001.00216.x.
52. Bursac, P., Lenormand, G., Fabry, B., Oliver, M., Weitz, D.A., Viasnoff, V., Butler, J.P., and Fredberg, J.J. (2005). Cytoskeletal remodelling and slow dynamics in the living cell. Nat Mater *4*, 557-561. 10.1038/nmat1404.
53. Matthews, B.D., Overby, D.R., Mannix, R., and Ingber, D.E. (2006). Cellular adaptation to mechanical stress: role of integrins, Rho, cytoskeletal tension and mechanosensitive ion channels. J Cell Sci *119*, 508-518. 10.1242/jcs.02760.
54. Guck, J., Schinkinger, S., Lincoln, B., Wottawah, F., Ebert, S., Romeyke, M., Lenz, D., Erickson, H.M., Ananthakrishnan, R., Mitchell, D., et al. (2005). Optical deformability as an inherent cell marker for testing malignant transformation and metastatic competence. Biophys J *88*, 3689-3698. 10.1529/biophysj.104.045476.
55. Byers, S.W., Sommers, C.L., Hoxter, B., Mercurio, A.M., and Tozeren, A. (1995). Role of E-cadherin in the response of tumor cell aggregates to lymphatic, venous and arterial flow: measurement of cell-cell adhesion strength. J Cell Sci *108 ( Pt 5)*, 2053-2064. 10.1242/jcs.108.5.2053.
56. Pajic-Lijakovic, I., Milivojevic, M., and McClintock, P.V.E. (2024). Epithelial cell-cell interactions in an overcrowded environment: jamming or live cell extrusion. J Biol Eng *18*, 47. 10.1186/s13036-024-00442-3.
57. Podbilewicz, B. (2006). Cell fusion. WormBook, 1-32. 10.1895/wormbook.1.52.1.

58. Mohler, W.A., Simske, J.S., Williams-Masson, E.M., Hardin, J.D., and White, J.G. (1998). Dynamics and ultrastructure of developmental cell fusions in the Caenorhabditis elegans hypodermis. Curr Biol *8*, 1087-1090. 10.1016/s0960-9822(98)70447-6.
59. Raymond, M.H., Davidson, A.J., Shen, Y., Tudor, D.R., Lucas, C.D., Morioka, S., Perry, J.S.A., Krapivkina, J., Perrais, D., Schumacher, L.J., et al. (2022). Live cell tracking of macrophage efferocytosis during Drosophila embryo development in vivo. Science *375*, 1182-1187. 10.1126/science.abl4430.
60. Ralston, K.S. (2015). Chew on this: amoebic trogocytosis and host cell killing by Entamoeba histolytica. Trends Parasitol *31*, 442-452. 10.1016/j.pt.2015.05.003.
61. Segovia-Juarez, J.L., Ganguli, S., and Kirschner, D. (2004). Identifying control mechanisms of granuloma formation during M. tuberculosis infection using an agent-based model. J Theor Biol *231*, 357-376. 10.1016/j.jtbi.2004.06.031.
62. Wang, Y., Bergman, D., Trujillo, E., Pearson, A.T., Sweis, R.F., and Jackson, T.L. (2023). Mathematical Model Predicts Tumor Control Patterns Induced by Fast and Slow CTL Killing Mechanisms. bioRxiv [preprint] *2023.07.19.548738*. 10.1101/2023.07.19.548738.
63. Osinska, I., Popko, K., and Demkow, U. (2014). Perforin: an important player in immune response. Cent Eur J Immunol *39*, 109-115. 10.5114/ceji.2014.42135.
64. Farhood, B., Najafi, M., and Mortezaee, K. (2019). CD8(+) cytotoxic T lymphocytes in cancer immunotherapy: A review. J Cell Physiol *234*, 8509-8521. 10.1002/jcp.27782.
65. Raskov, H., Orhan, A., Christensen, J.P., and Gogenur, I. (2021). Cytotoxic CD8(+) T cells in cancer and cancer immunotherapy. Br J Cancer *124*, 359-367. 10.1038/s41416-020-01048-4.
66. Espie, D., and Donnadieu, E. (2022). New insights into CAR T cell-mediated killing of tumor cells. Front Immunol *13*, 1016208. 10.3389/fimmu.2022.1016208.
67. Stinchcombe, J.C., Bossi, G., Booth, S., and Griffiths, G.M. (2001). The immunological synapse of CTL contains a secretory domain and membrane bridges. Immunity *15*, 751-761. 10.1016/s1074-7613(01)00234-5.
68. Halle, S., Keyser, K.A., Stahl, F.R., Busche, A., Marquardt, A., Zheng, X., Galla, M., Heissmeyer, V., Heller, K., Boelter, J., et al. (2016). In Vivo Killing Capacity of Cytotoxic T Cells Is Limited and Involves Dynamic Interactions and T Cell Cooperativity. Immunity *44*, 233-245. 10.1016/j.immuni.2016.01.010.
69. Weigelin, B., den Boer, A.T., Wagena, E., Broen, K., Dolstra, H., de Boer, R.J., Figdor, C.G., Textor, J., and Friedl, P. (2021). Cytotoxic T cells are able to efficiently eliminate cancer cells by additive cytotoxicity. Nat Commun *12*, 5217. 10.1038/s41467-021-25282-3.
70. Djuzenova, C.S., Rothfuss, A., Oppitz, U., Spelt, G., Schindler, D., Hoehn, H., and Flentje, M. (2001). Response to X-irradiation of Fanconi anemia homozygous and heterozygous cells assessed by the single-cell gel electrophoresis (comet) assay. Lab Invest *81*, 185-192. 10.1038/labinvest.3780226.
71. Reynolds, P., Cooper, S., Lomax, M., and O'Neill, P. (2015). Disruption of PARP1 function inhibits base excision repair of a sub-set of DNA lesions. Nucleic Acids Res *43*, 4028-4038. 10.1093/nar/gkv250.
72. Collins, A.R., and Azqueta, A. (2012). DNA repair as a biomarker in human biomonitoring studies; further applications of the comet assay. Mutat Res *736*, 122-129. 10.1016/j.mrfmmm.2011.03.005.
73. Norton, K.A., Gong, C., Jamalian, S., and Popel, A.S. (2019). Multiscale Agent-Based and Hybrid Modeling of the Tumor Immune Microenvironment. Processes (Basel) *7*. 10.3390/pr7010037.
74. Anderson, A.R.A. (2007). A Hybrid Multiscale Model of Solid Tumour Growth and Invasion: Evolution and the Microenvironment. In Single-Cell-Based Models in Biology and Medicine, pp. 3-28. 10.1007/978-3-7643-8123-3_1.
75. Hoehme, S., Friebel, A., Hammad, S., Drasdo, D., and Hengstler, J.G. (2017). Creation of Three-Dimensional Liver Tissue Models from Experimental Images for Systems Medicine. Methods Mol Biol *1506*, 319-362. 10.1007/978-1-4939-6506-9_22.
76. Finley, S.D., and Popel, A.S. (2013). Effect of tumor microenvironment on tumor VEGF during anti-VEGF treatment: systems biology predictions. J Natl Cancer Inst *105*, 802-811. 10.1093/jnci/djt093.
77. Swan, A., Hillen, T., Bowman, J.C., and Murtha, A.D. (2018). A Patient-Specific Anisotropic Diffusion Model for Brain Tumour Spread. Bull Math Biol *80*, 1259-1291. 10.1007/s11538-017-0271-8.
78. Chaplain, M.A., Graziano, L., and Preziosi, L. (2006). Mathematical modelling of the loss of tissue compression responsiveness and its role in solid tumour development. Math Med Biol *23*, 197-229. 10.1093/imammb/dql009.

79. Alarcon, T., Byrne, H.M., and Maini, P.K. (2003). A cellular automaton model for tumour growth in inhomogeneous environment. J Theor Biol *225*, 257-274. 10.1016/s0022-5193(03)00244-3.
80. Scott, J.G., Basanta, D., Anderson, A.R., and Gerlee, P. (2013). A mathematical model of tumour self-seeding reveals secondary metastatic deposits as drivers of primary tumour growth. J R Soc Interface *10*, 20130011. 10.1098/rsif.2013.0011.
81. Kaznatcheev, A., Vander Velde, R., Scott, J.G., and Basanta, D. (2017). Cancer treatment scheduling and dynamic heterogeneity in social dilemmas of tumour acidity and vasculature. Br J Cancer *116*, 785-792. 10.1038/bjc.2017.5.
82. Poleszczuk, J., Hahnfeldt, P., and Enderling, H. (2014). Biphasic modulation of cancer stem cell-driven solid tumour dynamics in response to reactivated replicative senescence. Cell Prolif *47*, 267-276. 10.1111/cpr.12101.
83. Powathil, G.G., Adamson, D.J., and Chaplain, M.A. (2013). Towards predicting the response of a solid tumour to chemotherapy and radiotherapy treatments: clinical insights from a computational model. PLoS Comput Biol *9*, e1003120. 10.1371/journal.pcbi.1003120.
84. Hamis, S., Nithiarasu, P., and Powathil, G.G. (2018). What does not kill a tumour may make it stronger: In silico insights into chemotherapeutic drug resistance. J Theor Biol *454*, 253-267. 10.1016/j.jtbi.2018.06.014.
85. Fortuna, I., Perrone, G.C., Krug, M.S., Susin, E., Belmonte, J.M., Thomas, G.L., Glazier, J.A., and de Almeida, R.M.C. (2020). CompuCell3D Simulations Reproduce Mesenchymal Cell Migration on Flat Substrates. Biophys J *118*, 2801-2815. 10.1016/j.bpj.2020.04.024.
86. Dunn, S.J., Appleton, P.L., Nelson, S.A., Nathke, I.S., Gavaghan, D.J., and Osborne, J.M. (2012). A two-dimensional model of the colonic crypt accounting for the role of the basement membrane and pericryptal fibroblast sheath. PLoS Comput Biol *8*, e1002515. 10.1371/journal.pcbi.1002515.
87. Schubert, M., Dokmegang, J., Yap, M.H., Han, L., Cavaliere, M., and Doursat, R. (2021). Computational modelling unveils how epiblast remodelling and positioning rely on trophectoderm morphogenesis during mouse implantation. Plos One *16*. 10.1371/journal.pone.0254763.
88. Camacho-Gómez, D., García-Aznar, J.M., and Gómez-Benito, M.J. (2022). A 3D multi-agent-based model for lumen morphogenesis: the role of the biophysical properties of the extracellular matrix. Engineering with Computers *38*, 4135-4149. 10.1007/s00366-022-01654-1.
89. Cess, C.G., and Finley, S.D. (2020). Multi-scale modeling of macrophage—T cell interactions within the tumor microenvironment. PLOS Computational Biology *16*. 10.1371/journal.pcbi.1008519.
90. Ruiz-Martinez, A., Gong, C., Wang, H., Sove, R.J., Mi, H., Kimko, H., and Popel, A.S. (2022). Simulations of tumor growth and response to immunotherapy by coupling a spatial agent-based model with a whole-patient quantitative systems pharmacology model. PLoS Comput Biol *18*, e1010254. 10.1371/journal.pcbi.1010254.
91. Ni, C., and Lu, T. (2022). Individual-Based Modeling of Spatial Dynamics of Chemotactic Microbial Populations. ACS Synth Biol *11*, 3714-3723. 10.1021/acssynbio.2c00322.
92. Hellweger, F.L., and Bucci, V. (2009). A bunch of tiny individuals—Individual-based modeling for microbes. Ecological Modelling *220*, 8-22. 10.1016/j.ecolmodel.2008.09.004.
93. Hastings, J., Owen, G., Dekker, A., Ennis, M., Kale, N., Muthukrishnan, V., Turner, S., Swainston, N., Mendes, P., and Steinbeck, C. (2016). ChEBI in 2016: Improved services and an expanding collection of metabolites. Nucleic Acids Res *44*, D1214-1219. 10.1093/nar/gkv1031.
94. Degtyarenko, K., de Matos, P., Ennis, M., Hastings, J., Zbinden, M., McNaught, A., Alcantara, R., Darsow, M., Guedj, M., and Ashburner, M. (2008). ChEBI: a database and ontology for chemical entities of biological interest. Nucleic Acids Res *36*, D344-350. 10.1093/nar/gkm791.
95. Cook, D.L., Mejino, J.L., Neal, M.L., and Gennari, J.H. (2008). Bridging biological ontologies and biosimulation: the ontology of physics for biology. AMIA Annu Symp Proc *2008*, 136-140.
96. Gkoutos, G.V., Mungall, C., Dolken, S., Ashburner, M., Lewis, S., Hancock, J., Schofield, P., Kohler, S., and Robinson, P.N. (2009). Entity/quality-based logical definitions for the human skeletal phenome using PATO. 2009 Annual International Conference of the IEEE Engineering in Medicine and Biology Society.
97. Meyer, C.T., Wooten, D.J., Paudel, B.B., Bauer, J., Hardeman, K.N., Westover, D., Lovly, C.M., Harris, L.A., Tyson, D.R., and Quaranta, V. (2019). Quantifying Drug Combination Synergy along Potency and Efficacy Axes. Cell Syst *8*, 97-108 e116. 10.1016/j.cels.2019.01.003.

98. Wooten, D.J., Meyer, C.T., Lubbock, A.L.R., Quaranta, V., and Lopez, C.F. (2021). MuSyC is a consensus framework that unifies multi-drug synergy metrics for combinatorial drug discovery. Nat Commun *12*, 4607. 10.1038/s41467-021-24789-z.
99. PhysiCell (2023). PhysiCell Version 1.12.0. https://github.com/MathCancer/PhysiCell/releases/tag/1.12.0.
100. Iwanaga, T., Usher, W., and Herman, J. (2022). Toward SALib 2.0: Advancing the accessibility and interpretability of global sensitivity analyses. Socio-Environmental Systems Modelling *4*. 10.18174/sesmo.18155.
101. Schälte, Y., Klinger, E., Alamoudi, E., and Hasenauer, J. (2022). pyABC: Efficient and robust easy-to-use approximate Bayesian computation. Journal of Open Source Software 7. 10.21105/joss.04304.
102. Foreman-Mackey, D., Hogg, D.W., Lang, D., and Goodman, J. (2013). emcee: The MCMC Hammer. Publications of the Astronomical Society of the Pacific *125*, 306-312. 10.1086/670067.
103. Salvatier, J., Wiecki, T.V., and Fonnesbeck, C. (2016). Probabilistic programming in Python using PyMC3. PeerJ Computer Science *2*. 10.7717/peerj-cs.55.
104. Olivier, A., Giovanis, D.G., Aakash, B.S., Chauhan, M., Vandanapu, L., and Shields, M.D. (2020). UQpy: A general purpose Python package and development environment for uncertainty quantification. Journal of Computational Science *47*. 10.1016/j.jocs.2020.101204.
105. PhysiCell Project (2021). 2021 PhysiCell Virtual Workshop and Hackathon. https://github.com/physicell-training/ws2021.
106. PhysiCell Project (2023). 2023 PhysiCell Virtual Workshop and Hackathon. https://github.com/physicell-training/ws2023.
107. PhysiCell Project (2022). 2022 PhysiCell Virtual Workshop and Hackathon. https://github.com/physicell-training/ws2022.
108. PhysiCell Project (2022). PhysiCell Mini-Workshop (part of the 2022 UCI short course in systems biology). https://github.com/physicell-training/UCI-sysbio-2022.
109. PhysiCell Project (2024). https://github.com/physicell-training/UCI_2024. https://github.com/physicell-training/UCI_2024.
110. PhysiCell Project (2023). PhysiCell Short Course for the 2023 CECAM ECM Workshop. https://github.com/physicell-training/cecam23.
111. PhysiCell Project (2023). PhysiCell Mini-Workshop for Northwestern University on June 8, 2023. https://github.com/physicell-training/nw2023.
112. Ruscone, M., Checcoli, A., Heiland, R., Barillot, E., Macklin, P., Calzone, L., and Noel, V. (2024). Building multiscale models with PhysiBoSS, an agent-based modeling tool. Brief Bioinform *25*. 10.1093/bib/bbae509.
113. Metzcar, J., Duggan, B.S., Fischer, B., Murphy, M., Heiland, R., and Macklin, P. (2025). A Simple Framework for Agent-Based Modeling with Extracellular Matrix. Bull Math Biol *87*, 43. 10.1007/s11538-024-01408-8.
114. Noel, V., Ruscone, M., Shuttleworth, R., and Macnamara, C.K. (2024). PhysiMeSS - a new physiCell addon for extracellular matrix modelling. GigaByte *2024*, gigabyte136. 10.46471/gigabyte.136.
