# Supplementary material for: Human interpretable grammar encodes multicellular systems biology models to democratize virtual cell laboratories
Source: Cell. Author manuscript; Available in PMC 2026 Mar 24. (PMC13012569; doi:10.1016/j.cell.2025.06.048)
Supplement: 16 [file NIHMS2100468-supplement-16.pdf]

CellPress
OPEN ACCESS

Supplemental figures

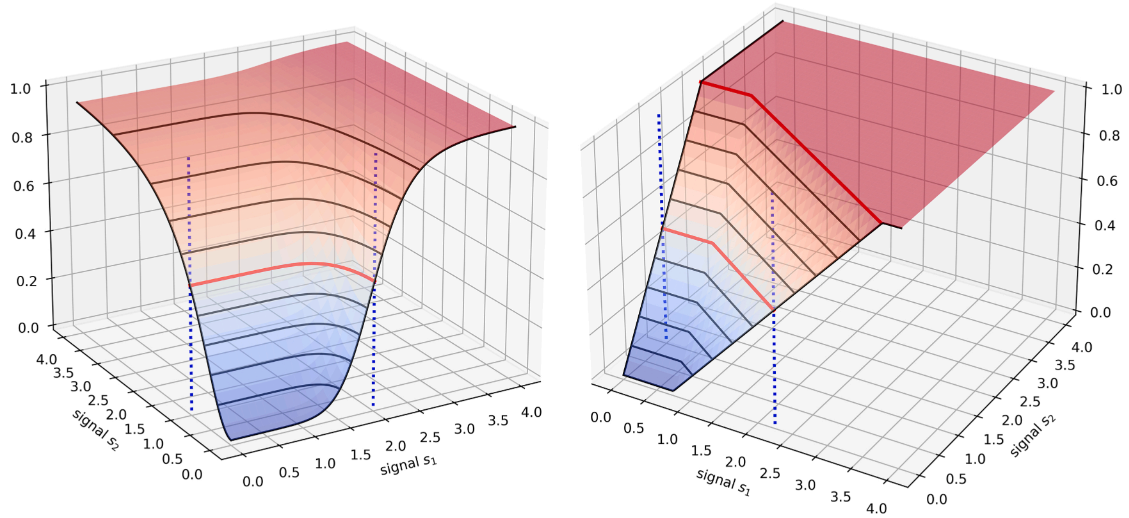

Figure S1. Multivariate response functions in STAR Methods

Left: a multivariate Hill response function, where s1 has half-max 2 and Hill power 8, and s2 has half-max 1 and Hill power 2. Half-maxes are plotted on each variable as blue dashed lines, while the half-max across the multivariate function is plotted as a red contour. H(s1) and H(s2) are plotted as black curves along the respective axes.

Right: a multivariate linear response function, where s1 has min and max thresholds 0.75 and 3.75, and s2 has thresholds 0 and 2. Half-maxes are plotted on each variable as blue dashed lines, while the half-max across the multivariate function is plotted as a red contour. L(s1) and L (S2) are plotted as black curves along the respective axes. Related to STAR Methods.

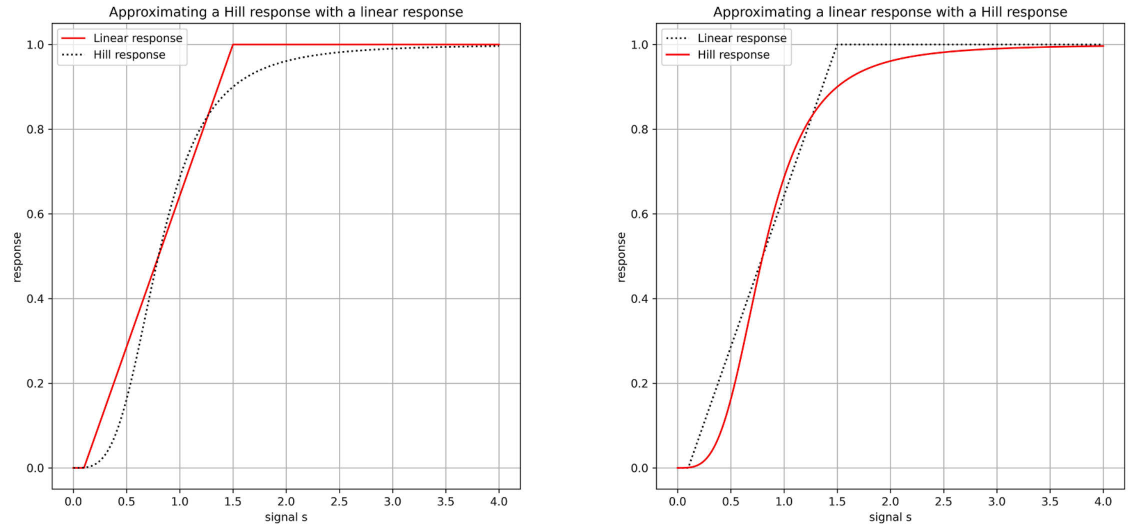

Figure S2. Converting between linear and Hill response functions, related to STAR Methods
Left: approximating a linear response function (black dotted curve) with a Hill response function (red).
Right: approximating a Hill response function (black dotted) with a linear response function (red).

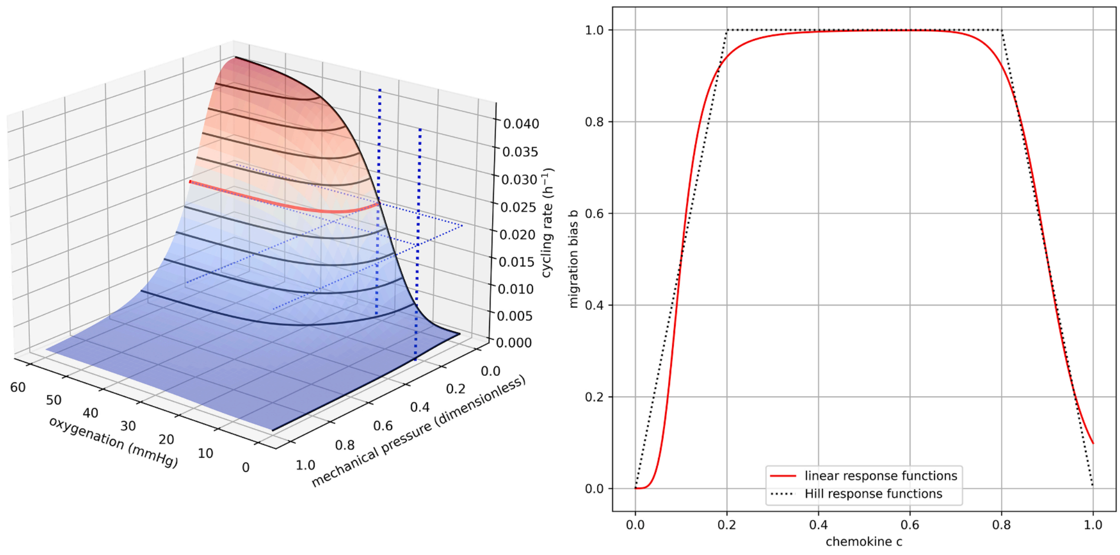

Figure S3. Sample response functions, related to STAR Methods

Left: the behavioral response function for the statements "oxygen increases cycle entry" and "pressure decreases cycle entry," using the multivariate Hill response function.

Right: a non-monotonic response function from the statements "*c* increases migration bias" (for lower values of *c*) and "*c* decreases migration bias" (for higher values of *c*), showing both linear and Hill response constructions.

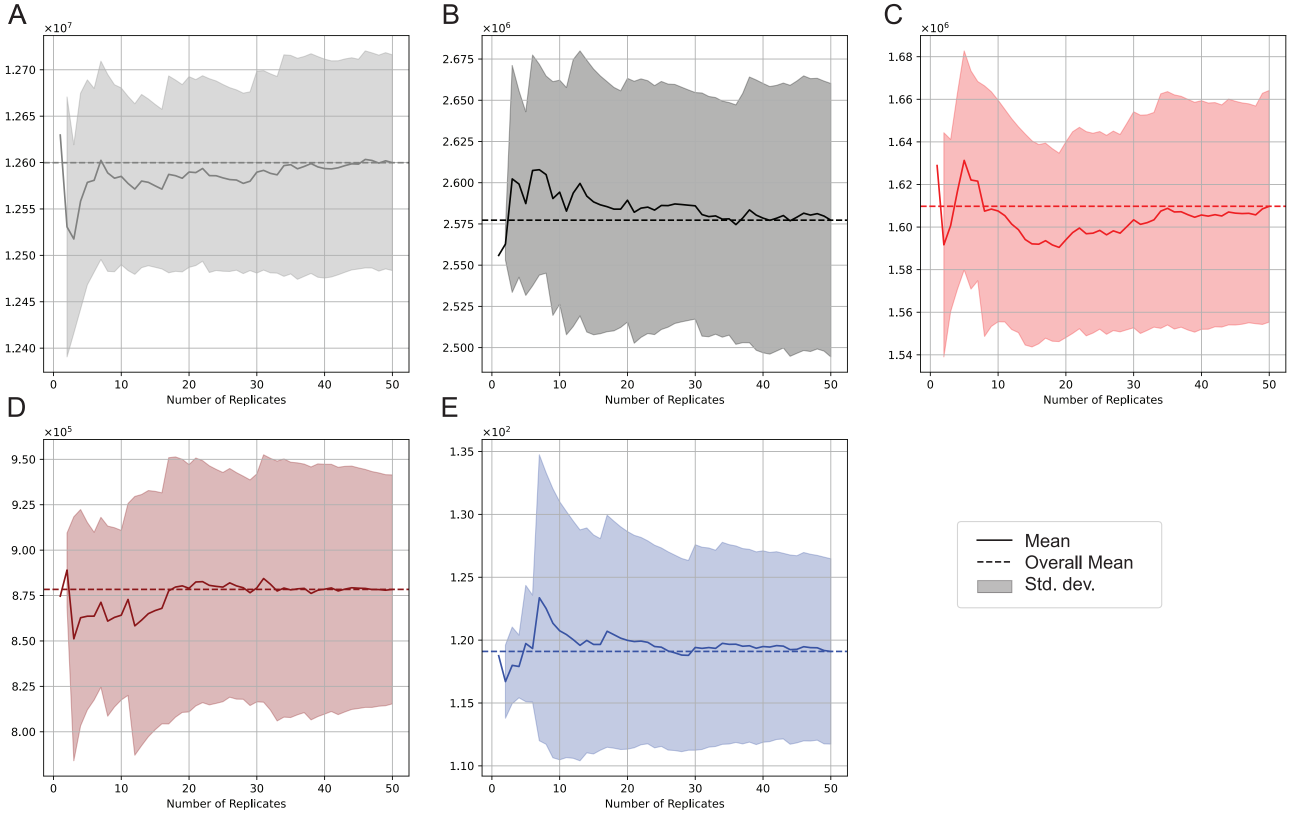

Figure S4. Convergence of QoIs across the replicates in hypoxia model, related to Figure 2

AUC of live (A) and dead (B) non-motile tumor cells. AUC of live (C) and dead (D) motile tumor cells. (E) Wassertein distance of radial distributions between non-motile and motile tumor cells.

Panc10.05 monoculture

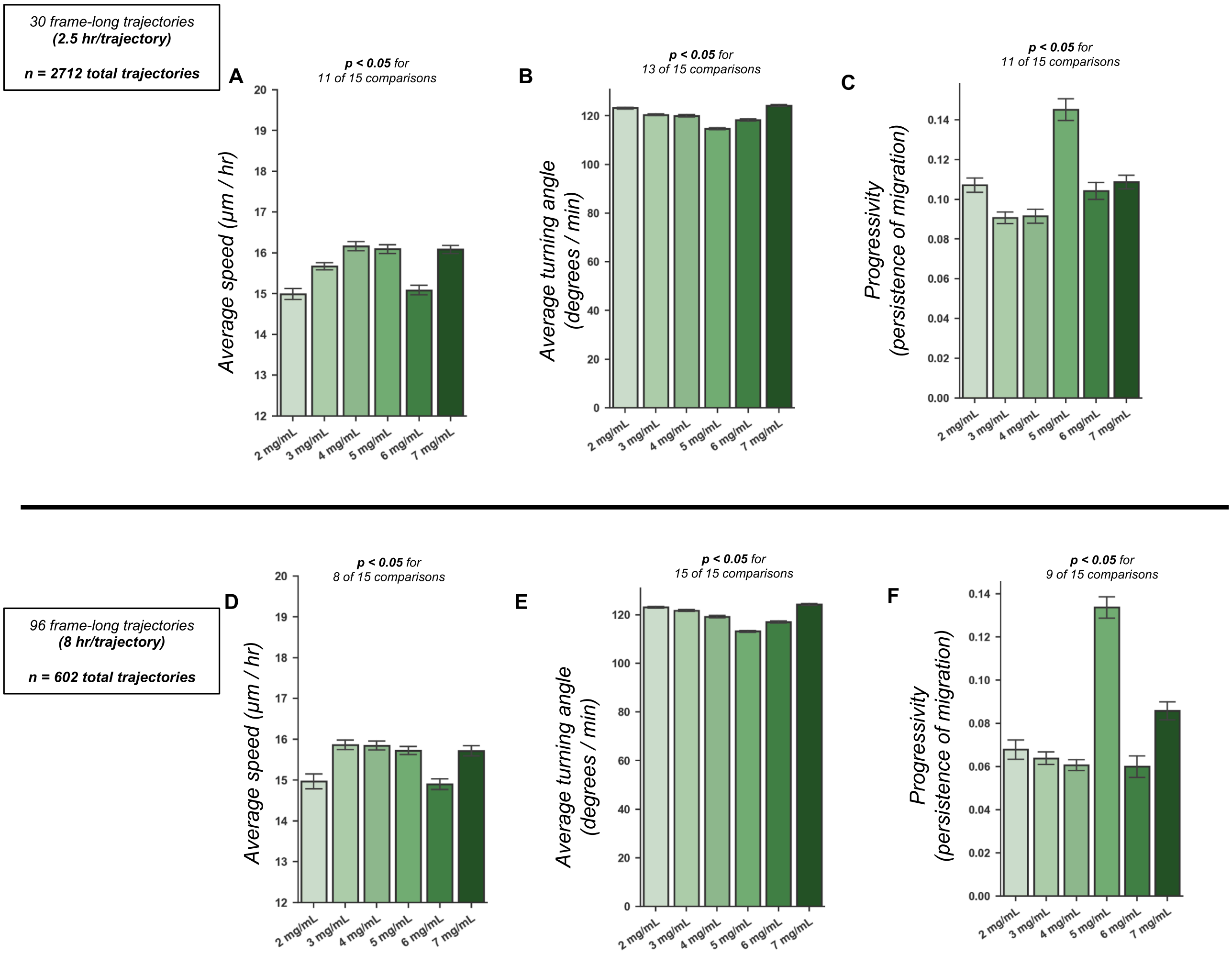

Figure S5. PANC10.05 cell motility in monoculture or co-culture, related to Figure 3

Comparing motility behavior of PANC10.05 cells in monoculture or in co-culture with HT-231 CAFs assayed using 30-frame-long trajectories (A–C) and 96-frame-long trajectories (D–F). Average speed (μm/h) (A), average turning angle (degrees/min) (B), and progressivity (C) of PANC10.05 monoculture and co-culture in varying ECM densities. (D–F) equivalent metrics from the 96-frame trajectory experiment.

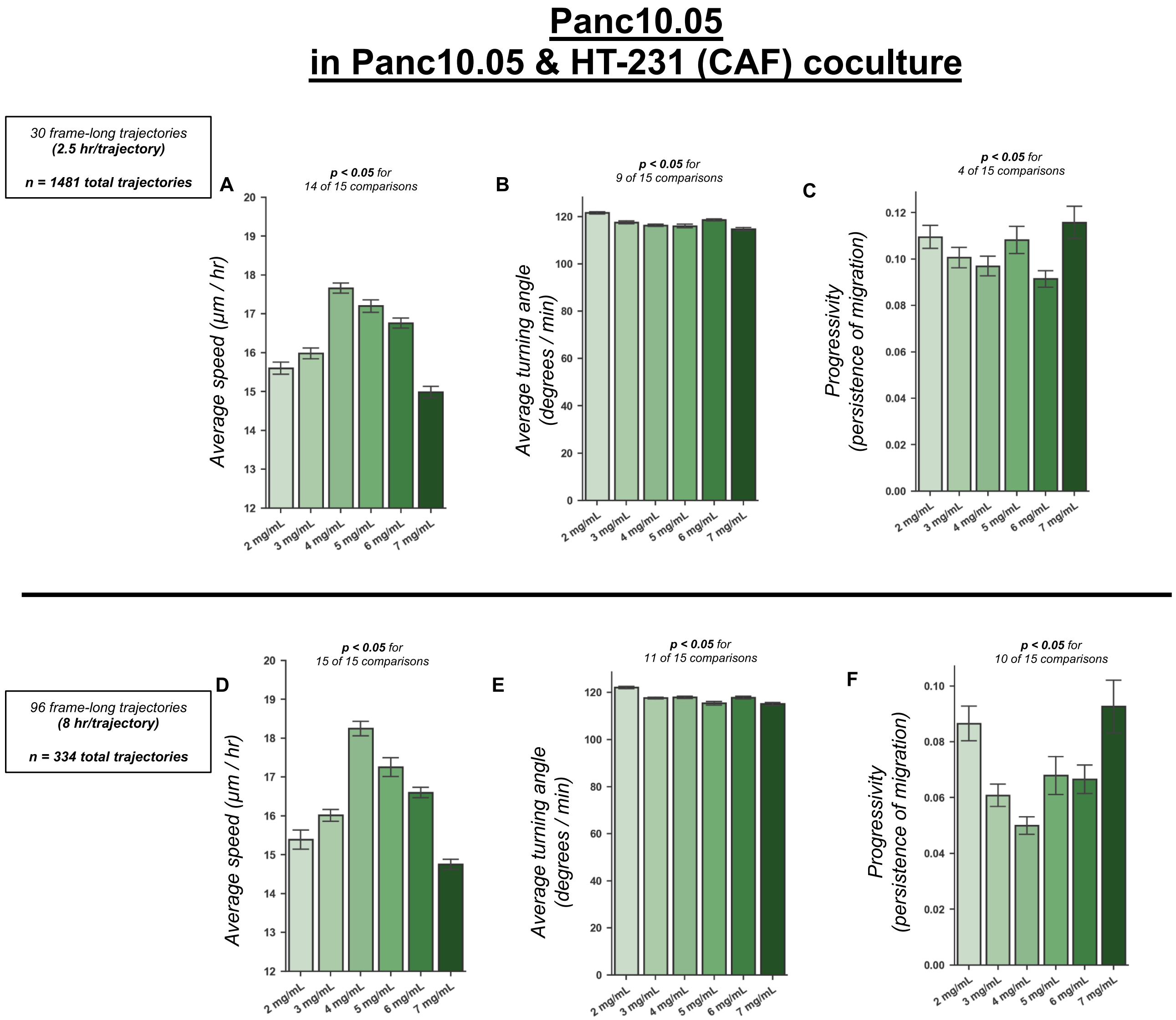

Figure S6. PANC10.05 cell motility monoculture, related to Figure 3

Motility behavior of PANC10.05 cells in monoculture assayed using 30-frame-long trajectories (A–C) and 96-frame-long trajectories (D–F). Average speed (μm/h) (A), average turning angle (degrees/min) (B), and progressivity (C) of PANC10.05 monoculture in varying ECM densities. (D–F) equivalent metrics from the 96-frame trajectory experiment.

CellPress
OPEN ACCESS

Comparing Panc10.05 in monocultures vs in cocultures

Interesting note - The presence of CAFs seem to increase Panc10.05 speed ***except*** *at the highest of ECM densities (7.0 mg/mL), where it exhibits decreased speed*

30 frame-long trajectories
(2.5 hr/trajectory)

n = 4193 total trajectories

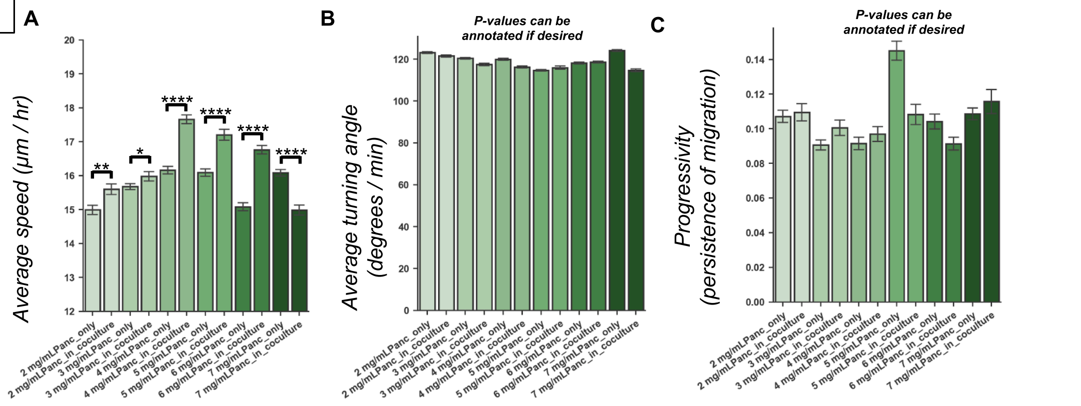

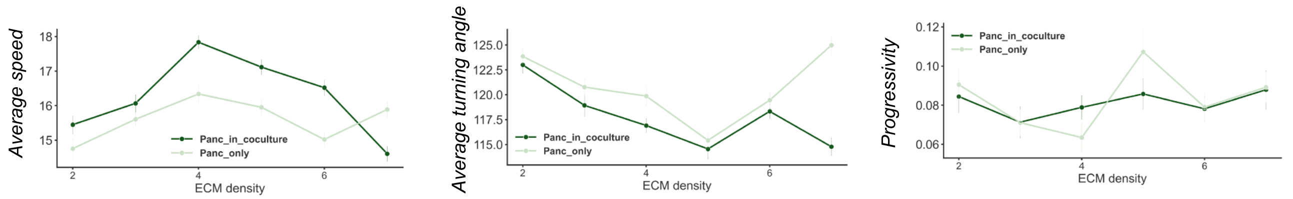

96 frame-long trajectories
(8 hr/trajectory)

n = 936 total trajectories

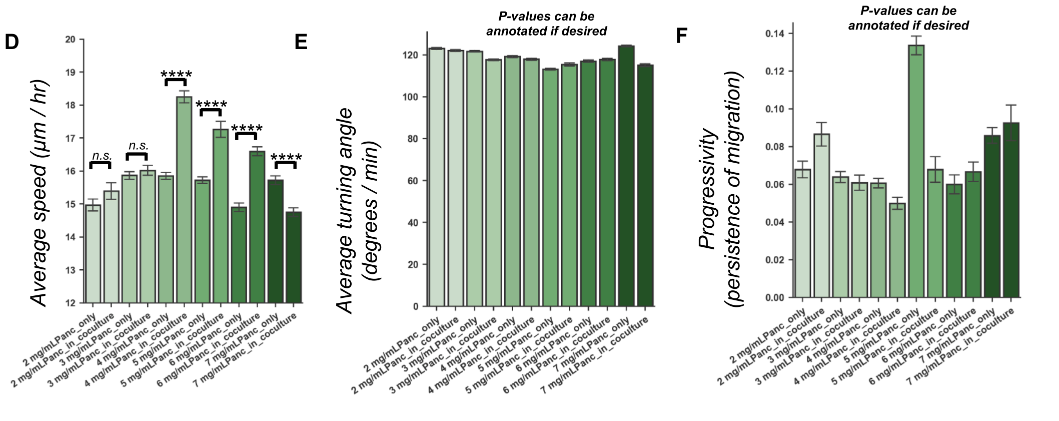

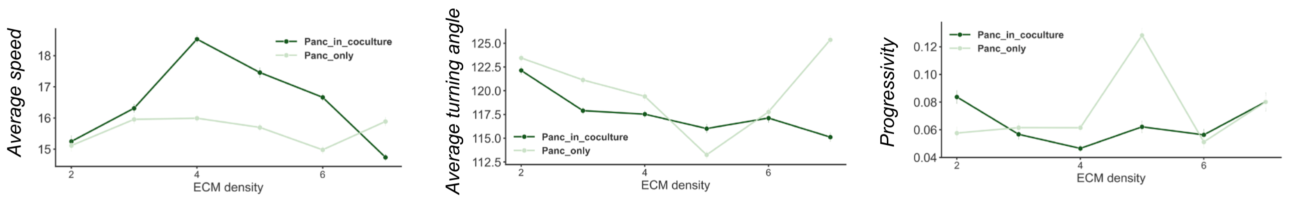

(legend on next page)

Figure S7. PANC10.05 cell motility in co-culture, related to Figure 3

Motility behavior of PANC10.05 cells in co-culture with HT-231 CAFs assayed using 30-frame-long trajectories (A–C) and 96-frame-long trajectories (D–F). Average speed (μm/h) (A), average turning angle (degrees/min) (B), and progressivity (C) of PANC10.05 in co-culture with HT-231 in varying ECM densities. (D–F) equivalent metrics from the 96-frame trajectory experiment.

HT-231 (CAF) monoculture

30 frame-long trajectories
(2.5 hr/trajectory)

n = 371 total trajectories

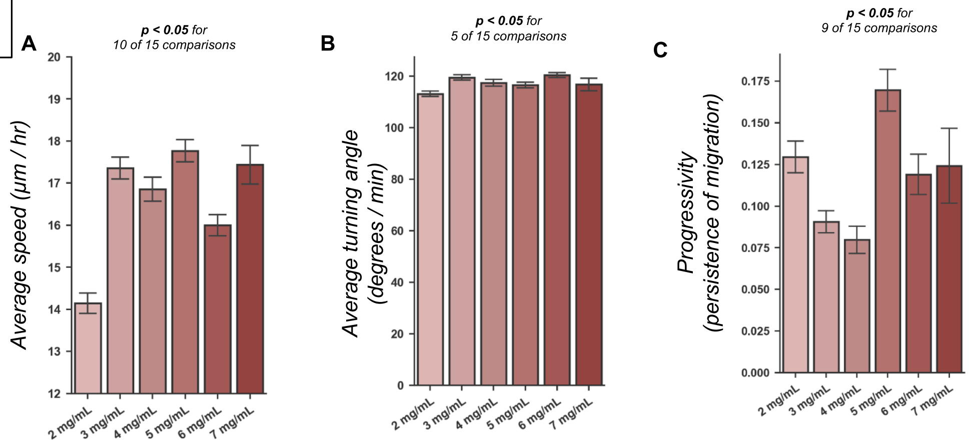

96 frame-long trajectories
(8 hr/trajectory)

Too few trajectories tracked

HT-231 (CAF)
in Panc10.05 & HT-231 (CAF) coculture

30 frame-long trajectories
(2.5 hr/trajectory)

n = 142 total trajectories

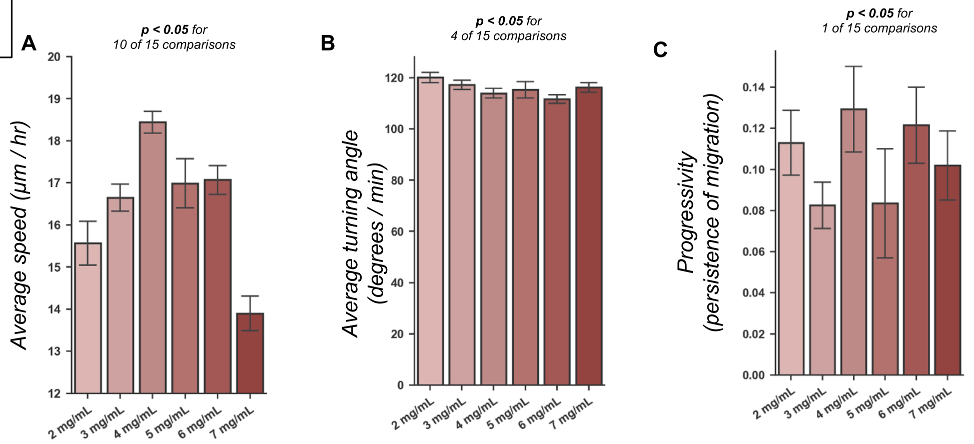

96 frame-long trajectories
(8 hr/trajectory)

Too few trajectories tracked

(legend on next page)

Figure S8. HT-231 CAF motility in monoculture or co-culture, related to Figure 3

Comparing motility behavior of HT-231 CAFs in monoculture or in co-culture with PANC10.05 cells assayed using 30-frame-long trajectories (A–C) and 96-frame-long trajectories (D–F). Average speed (μm/h) (A), average turning angle (degrees/min) (B), and progressivity (C) of HT-231 monoculture and co-culture in varying ECM densities. (D–F) equivalent metrics from the 96-frame trajectory experiment.

CellPress
OPEN ACCESS

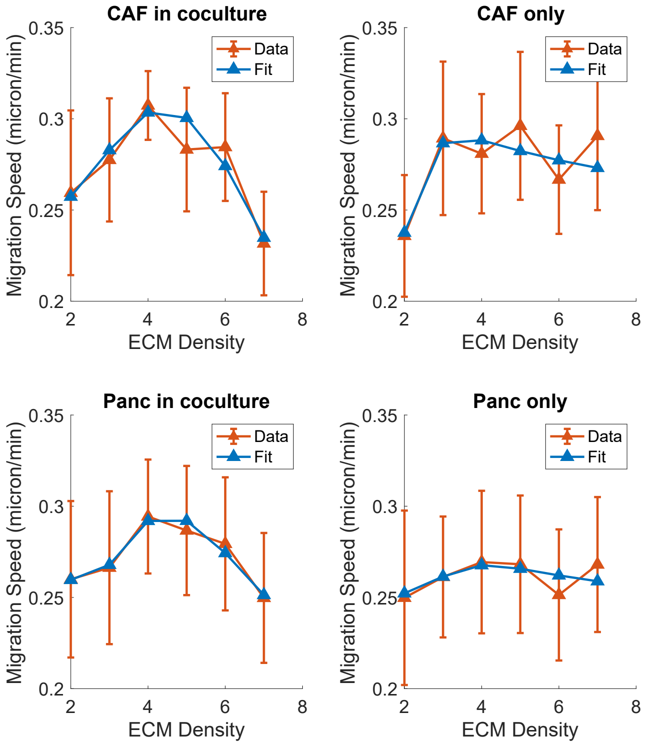

Figure S9. Fibroblast and pancreatic cell motility response curves, related to Figure 3

Motility behavior of simulated fibroblasts and pancreatic tumor cells is specified using two Hill curves that come together to fit the experimental data describing the relationship between migration speed and ECM density for each cell type (S5–S8).

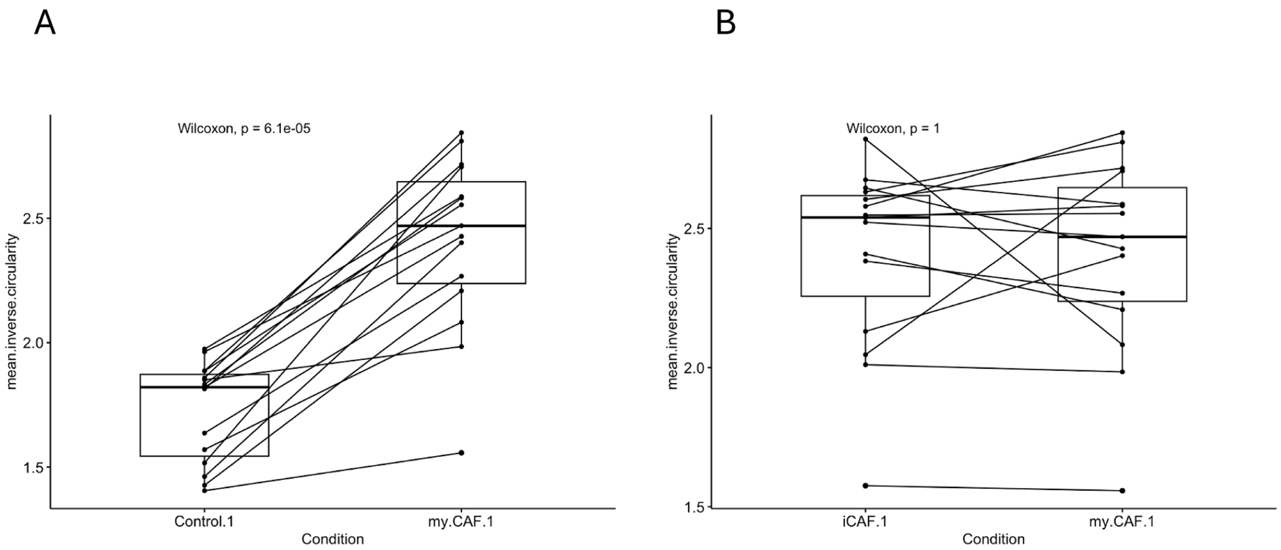

Figure S10. Invasiveness of patient-derived organoids, related to Figure 3
(A) Comparison of mean inverse circularity of PDOs between myCAF-conditioned and control media.
(B) Comparison of mean inverse circularity of PDOs between iCAF-conditioned and myCAF-conditioned media.

CellPress
OPEN ACCESS

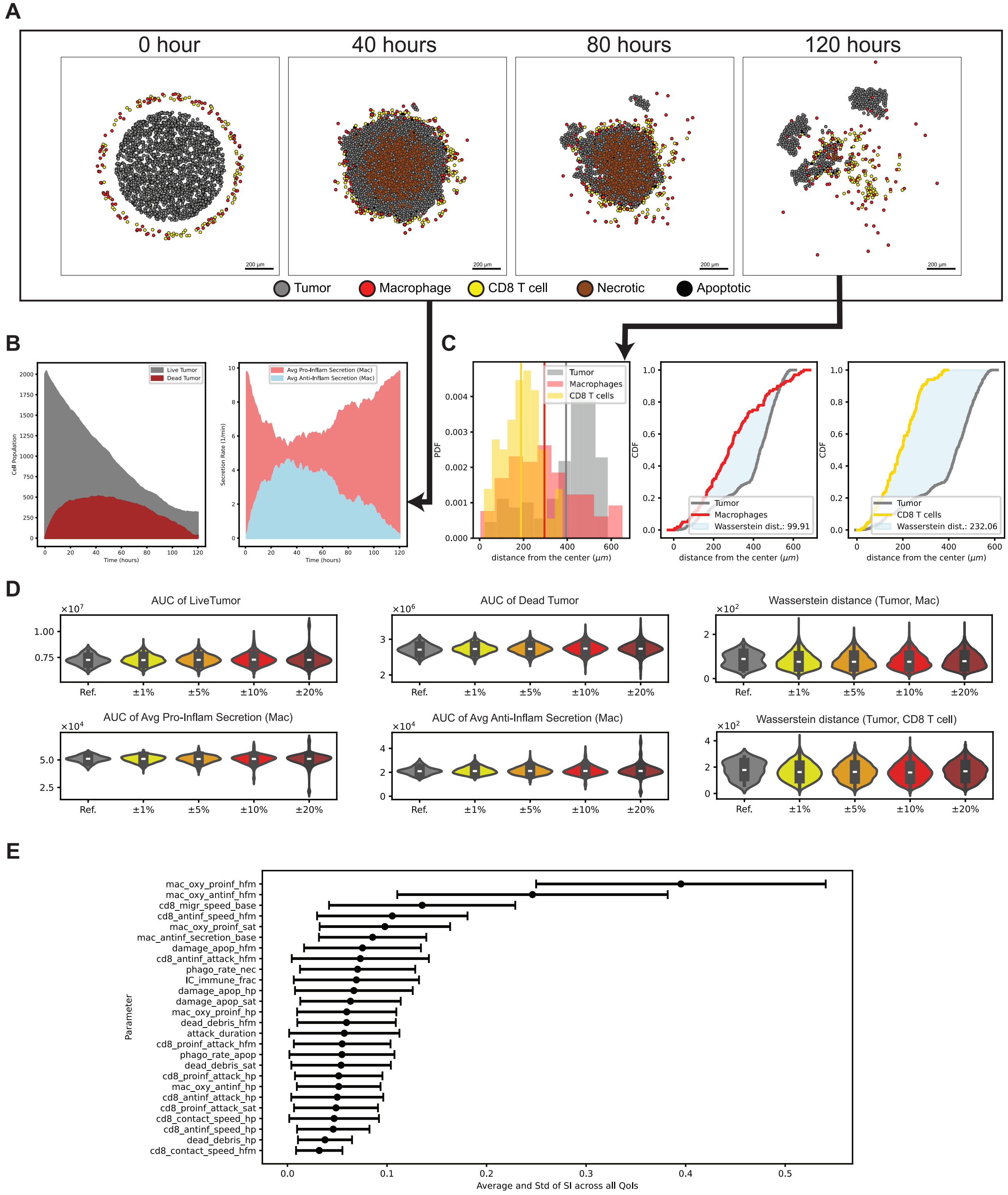

Figure S11. Local sensitivity analysis of the simple tumor-immune model, related to Figure 4
(A) Simulation of a 5-day evolution using reference parameters.
(B) Area under curve (AUC) for cell populations, extracted from the time course.

(legend continued on next page)

(C) Radial distributions at the final snapshot (day 5) are shown in left plot, while the Wassertein distances extracted from these distributions are presented in the middle plot (macrophages vs. tumor cells) and the right plot (CD8 T cells vs. tumor cells).
(D) Variation in quantities of interest (QoIs) under multiplicative perturbations in the 26D parameter space.
(E) Average and standard deviation of the sensitivity index for each parameter across all QoIs.

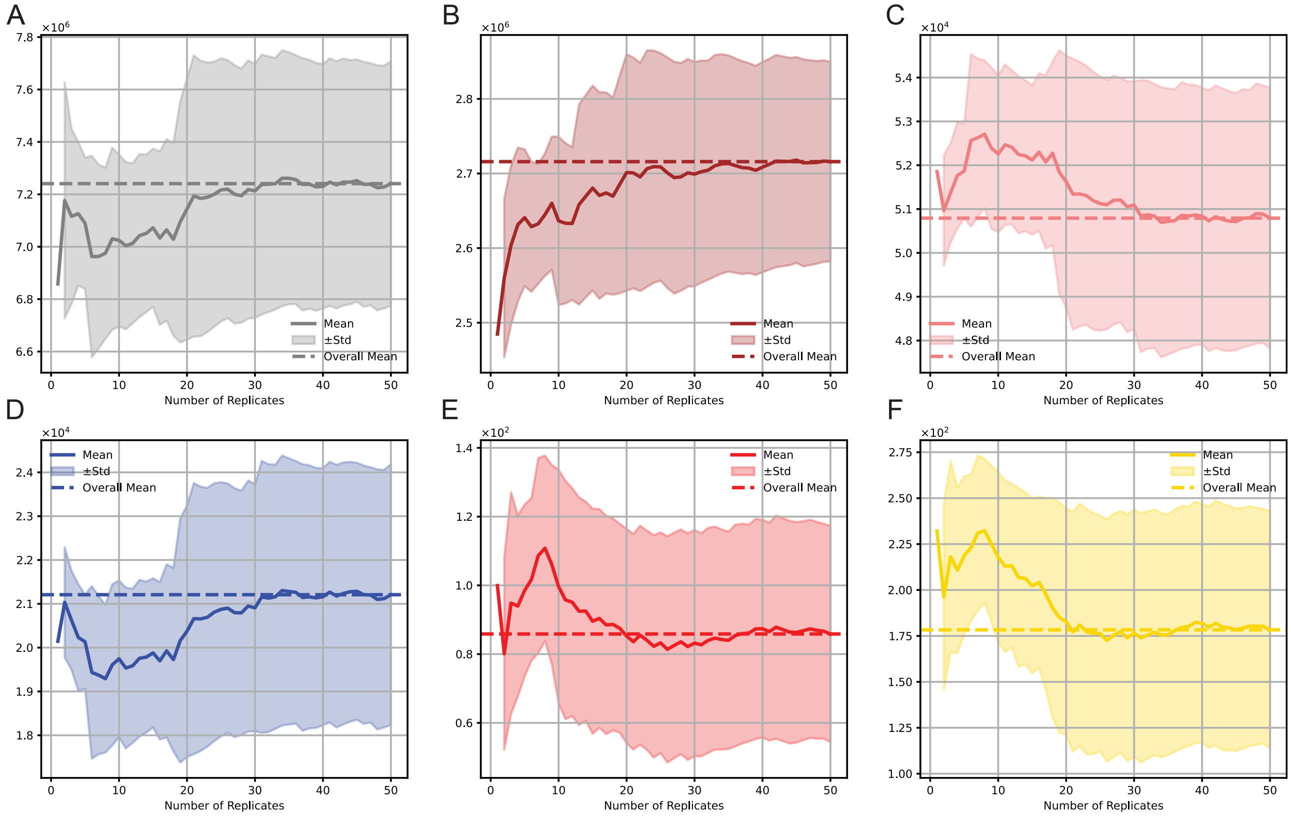

Figure S12. Convergence of QoIs across the replicates in simple tumor-immune model, related to Figure 4

AUC of live (A) and dead (B) tumor cells. AUC of pro-inflammatory (C) and anti-inflammatory (D) secretion rate of macrophages. Wassertein distance of radial distributions between tumor cells and macrophages (E) and tumor cells and $CD8^+$ T cells (F).

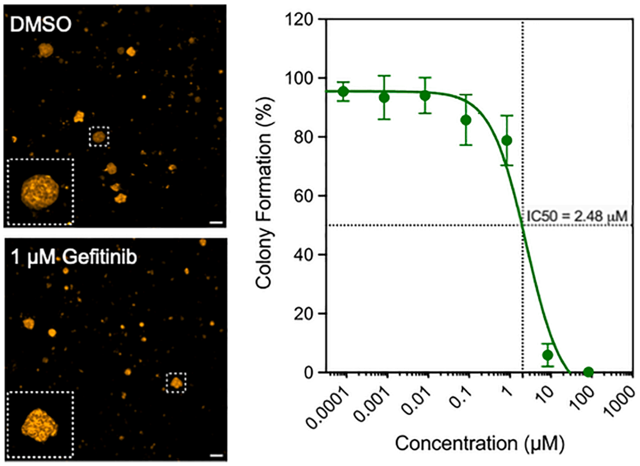

Figure S13. Gefitinib colony formation assay, related to Figure 5

Tumor cells are seeded and the number of successful colonies formed is compared between control and gefitinib (EGFR inhibitor). This shows that inhibiting EGFR signaling does not inhibit colony formation behavior in these tumor cells.

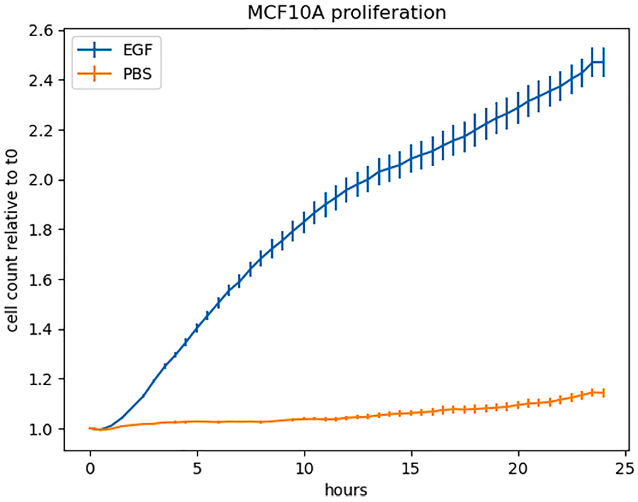

Figure S14. EGF impact on MCF10A proliferation, related to Figure 5

EGF increases MCF10A cell proliferation vs. PBS control over a 24-h time frame.

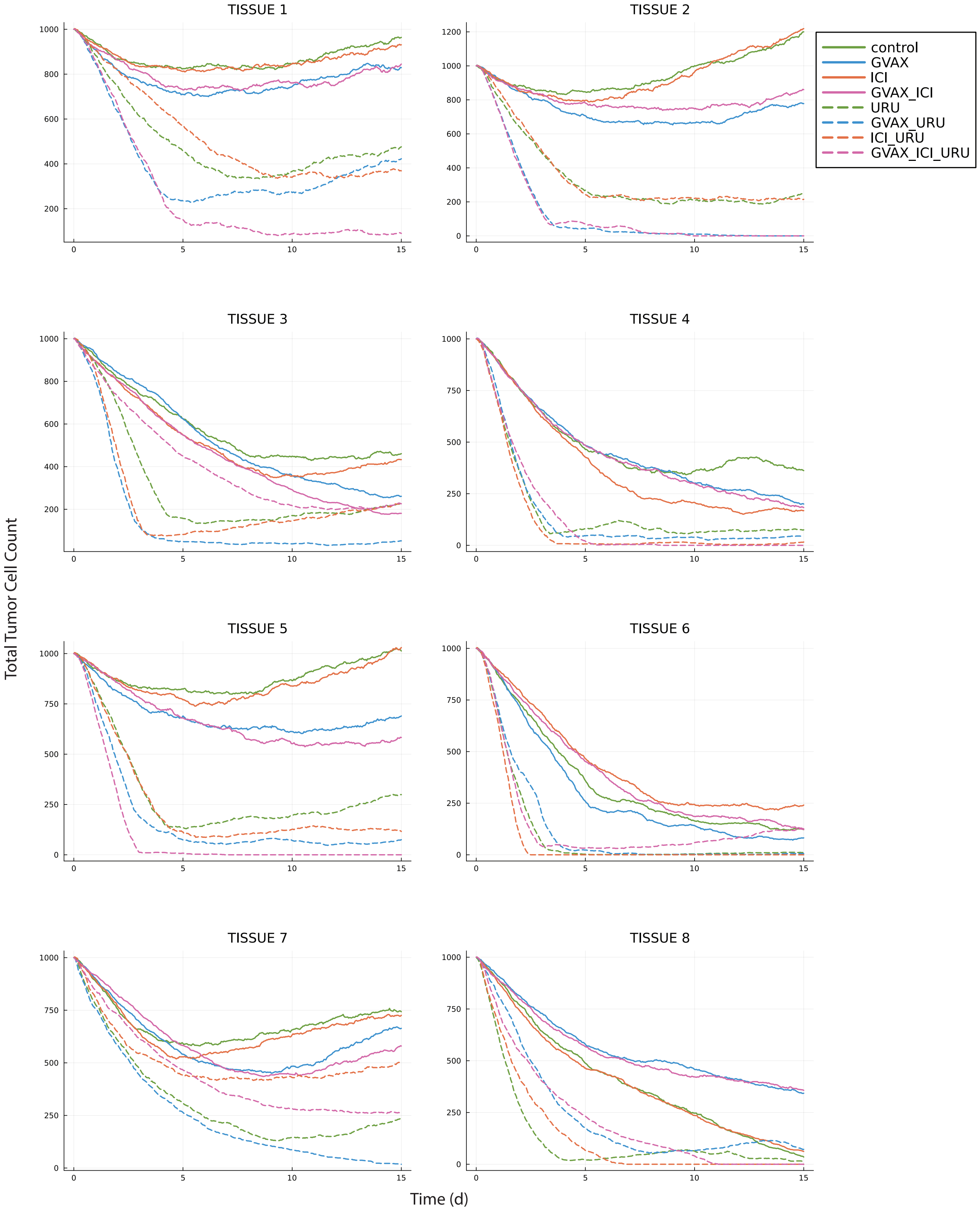

Figure S15. Simulated tumor cell growth under therapy combinations vs. untreated virtual control, related to Figure 6
Time series of simulated tumor cell counts under baseline and combination therapy conditions for PDAC tissues 1–8.

CellPress
OPEN ACCESS

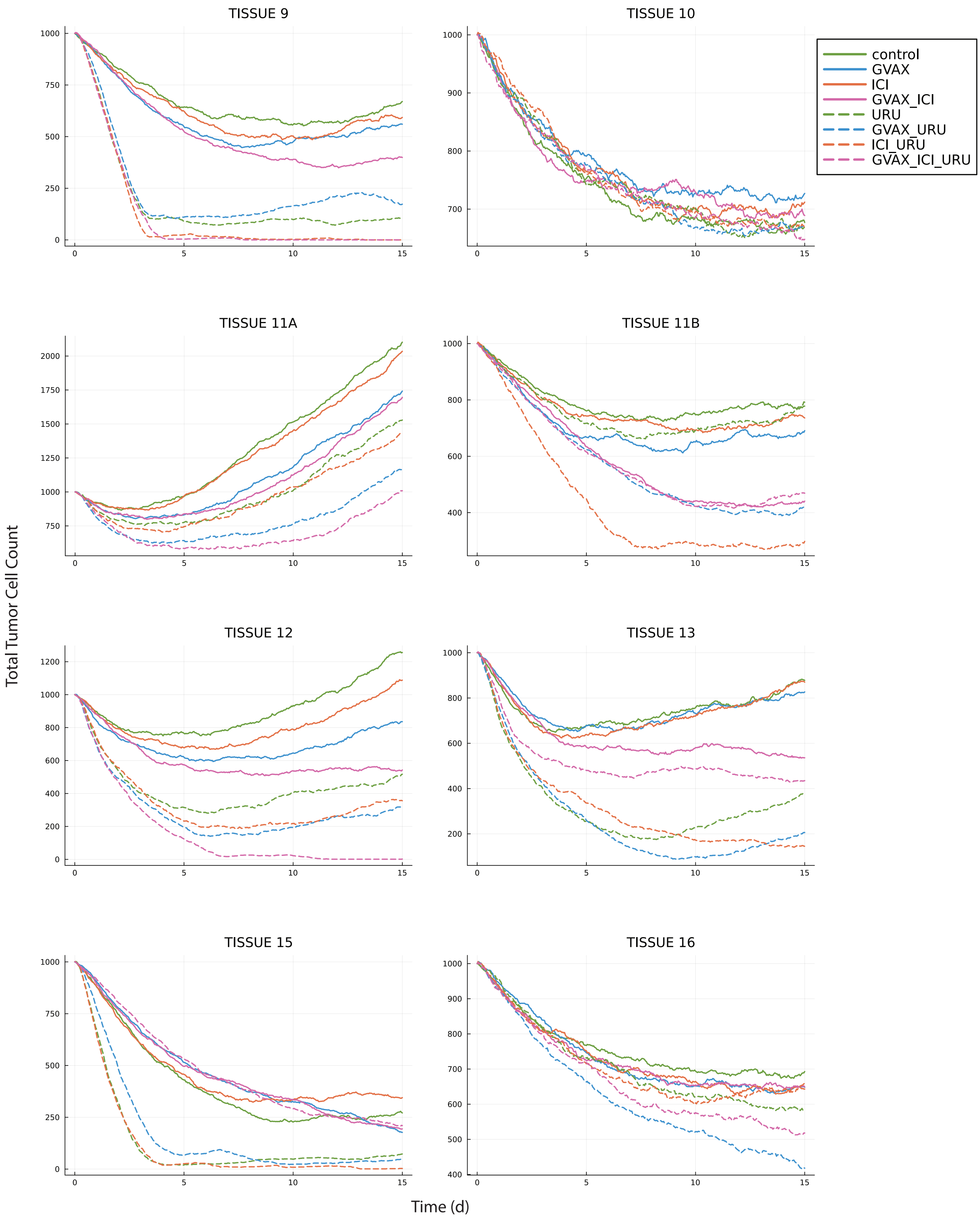

Figure S16. Simulated tumor cell growth under treatment and control for PDAC tissues 9–16, related to Figure 6

Time series of simulated tumor cell counts under baseline and combination therapy conditions for PDAC tissues 9–16.

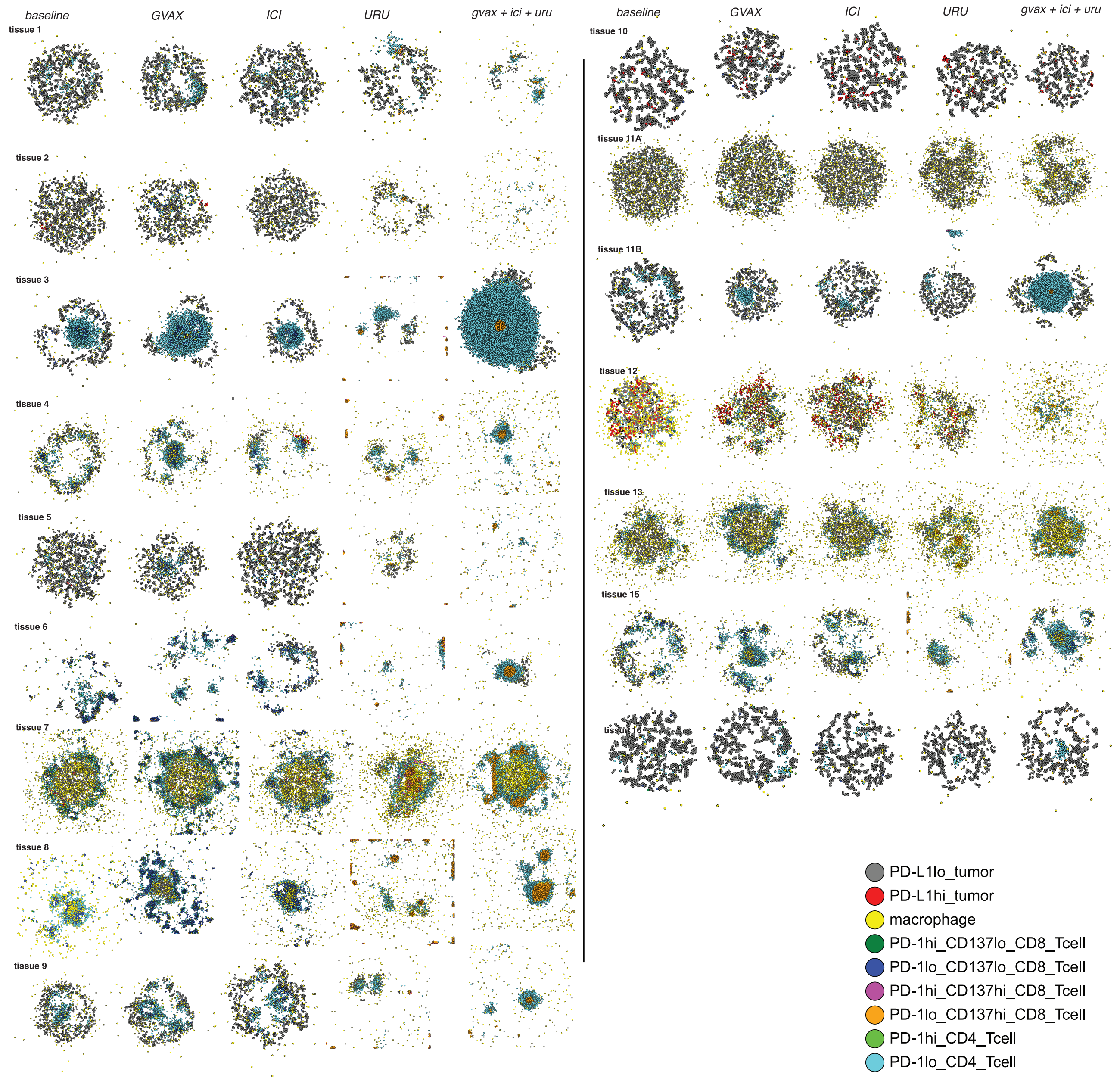

Figure S17. Simulated tissue endpoints under single- and triple-combination treatments and virtual control, related to Figure 6
Snapshots of agents at simulation endpoints for each tissue, baseline, single therapies, and triple-combination therapy shown.

CellPress
OPEN ACCESS

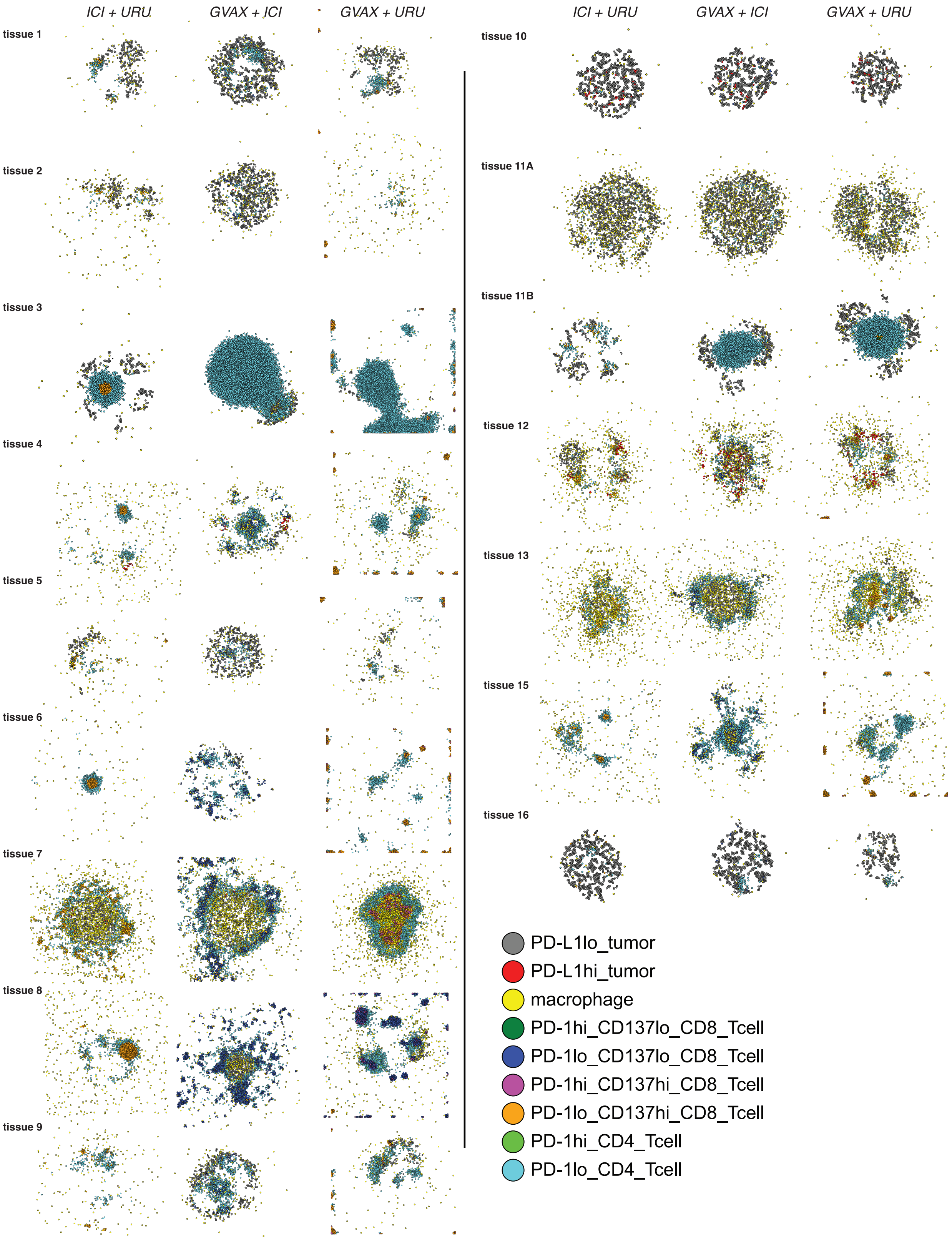

Figure S18. Simulated tissue endpoints under double-combination treatments, related to Figure 6
Snapshots of agents at simulation endpoints for each tissue, double-combination therapies shown.

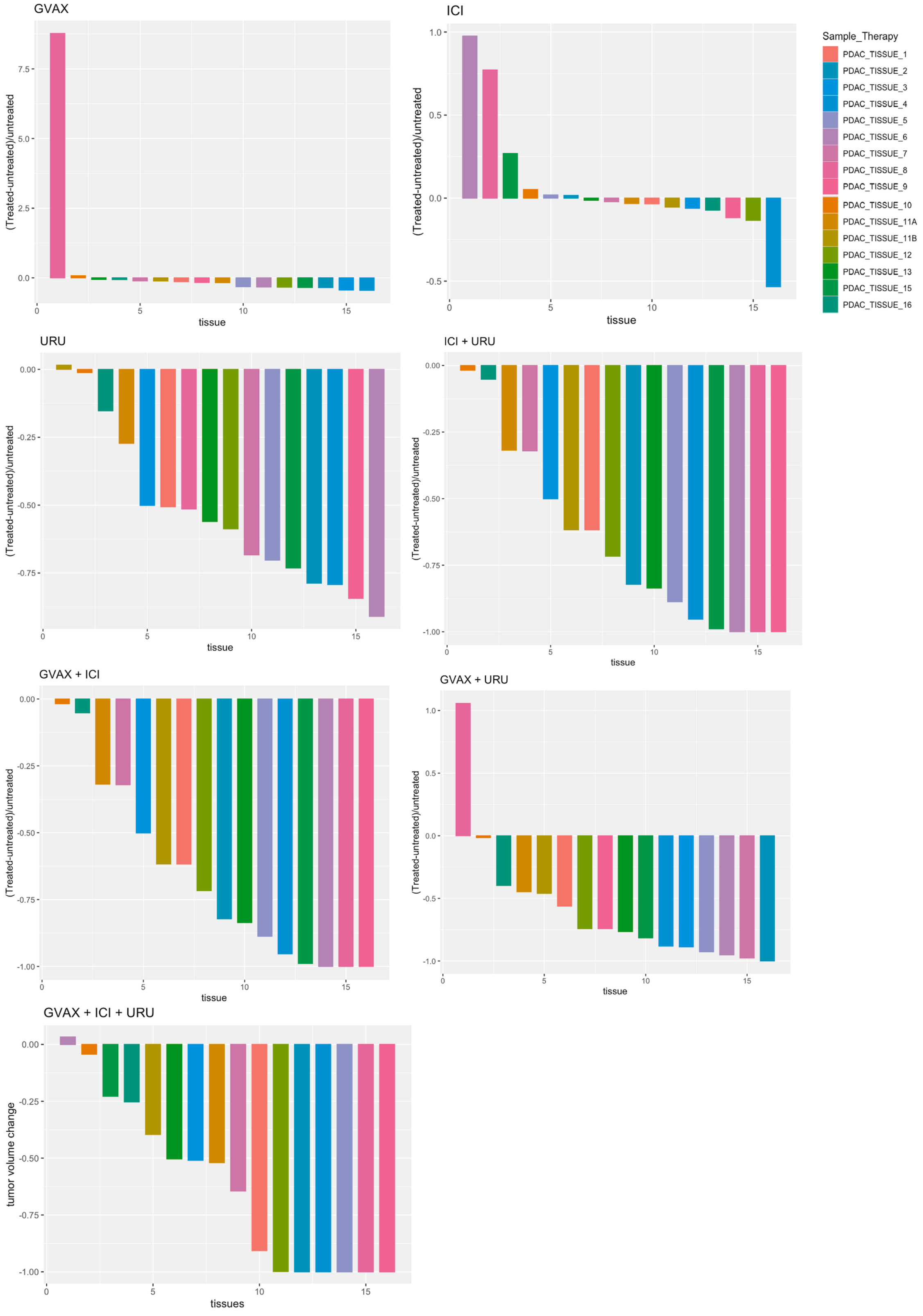

(legend on next page)

CellPress
OPEN ACCESS

Figure S19. Waterfall plots of treatment efficacy for simulated tissue, related to Figure 6

Waterfall plots showing efficacy of each simulated therapy in each tissue. Positive values indicated tumor grew relative to baseline final volume; negative values indicate tumor shrinkage.

Therapy: GVAX + ICI + URU

compare baseline immune population abundances to therapeutic efficacy in each simulated tissue

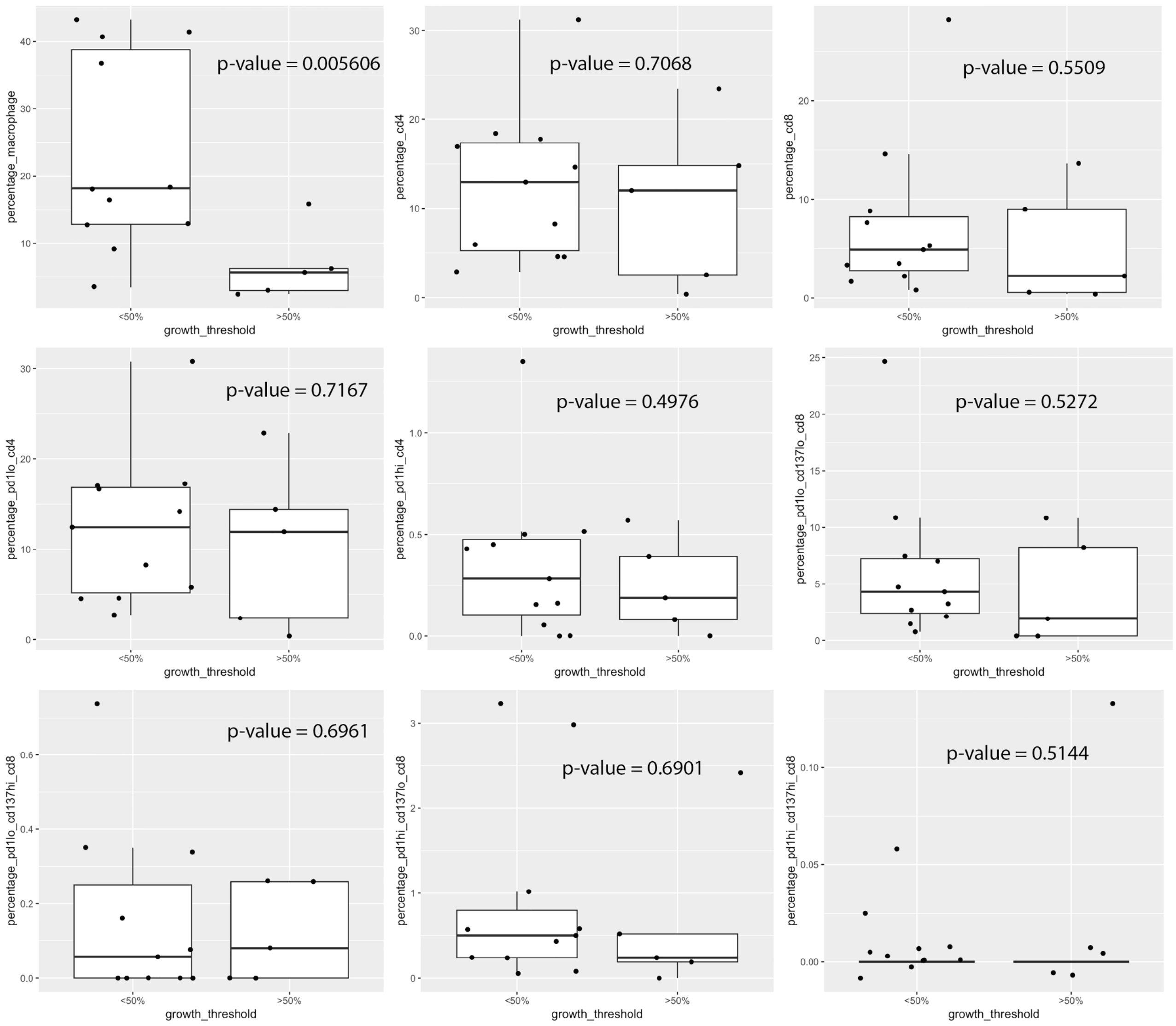

Figure S20. Immune cell abundances in GVAX + ICI + URU treatment, related to Figure 6

Immune population abundances in tissues, binned by whether the simulation with GVAX + ICI + URU reached the tumor clearance threshold. Macrophage abundance was significantly higher in tissues whose simulations reached the tumor clearance threshold.

Therapy: GVAX

compare baseline immune population abundances to therapeutic efficacy in each simulated tissue

no tissues met the threshold of 50% tumor volume reduction relative to baseline

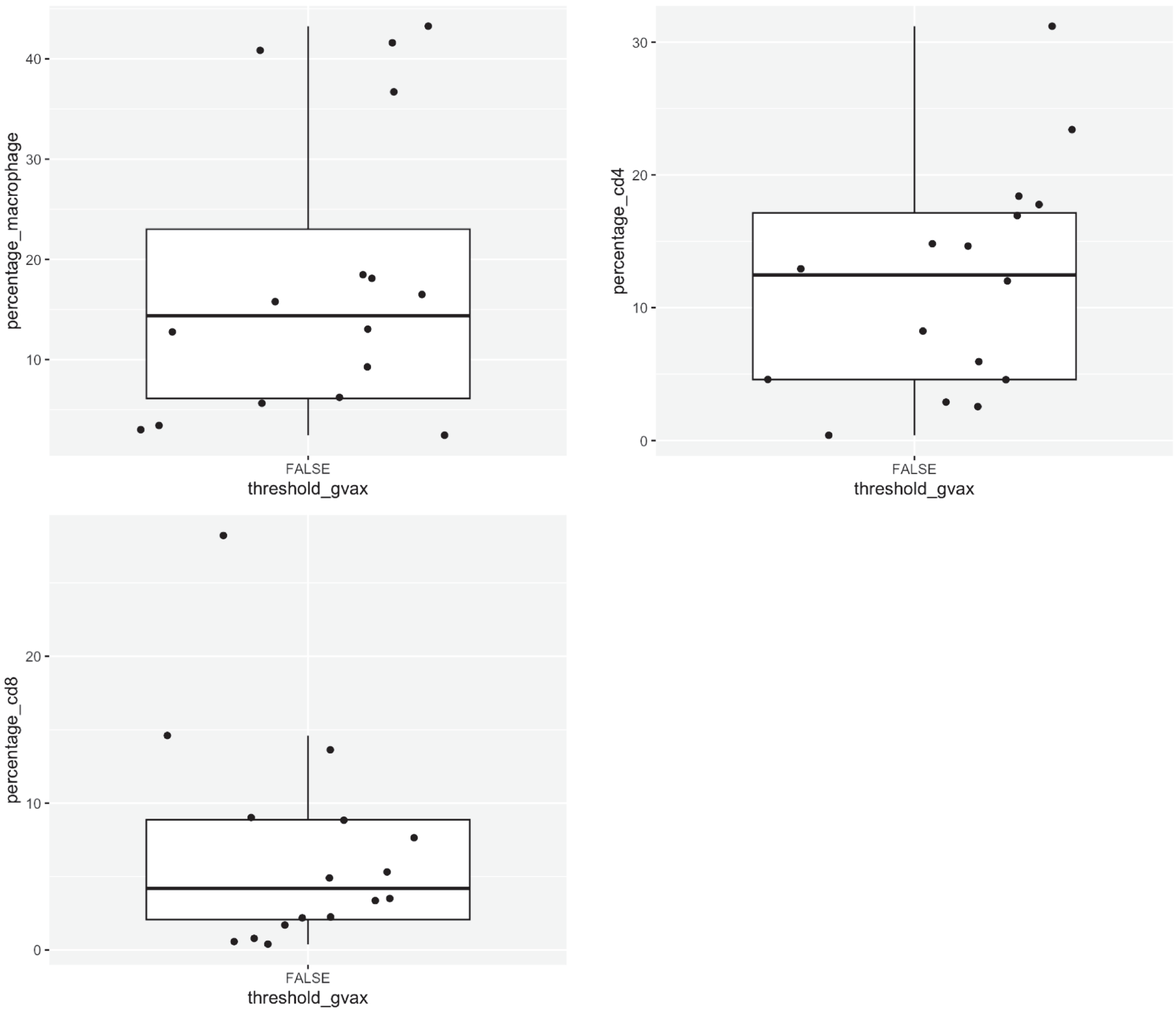

Figure S21. Immune cell abundances in GVAX treatment, related to Figure 6

Shown here is the abundance of macrophages and CD4 and CD8 T cells in each tissue. With the GVAX treatment, no tissues reached the threshold of 50% clearance relative to baseline.

Therapy: ICI

compare baseline immune population abundances to therapeutic efficacy in each simulated tissue

(not enough observations in "true" for t test)

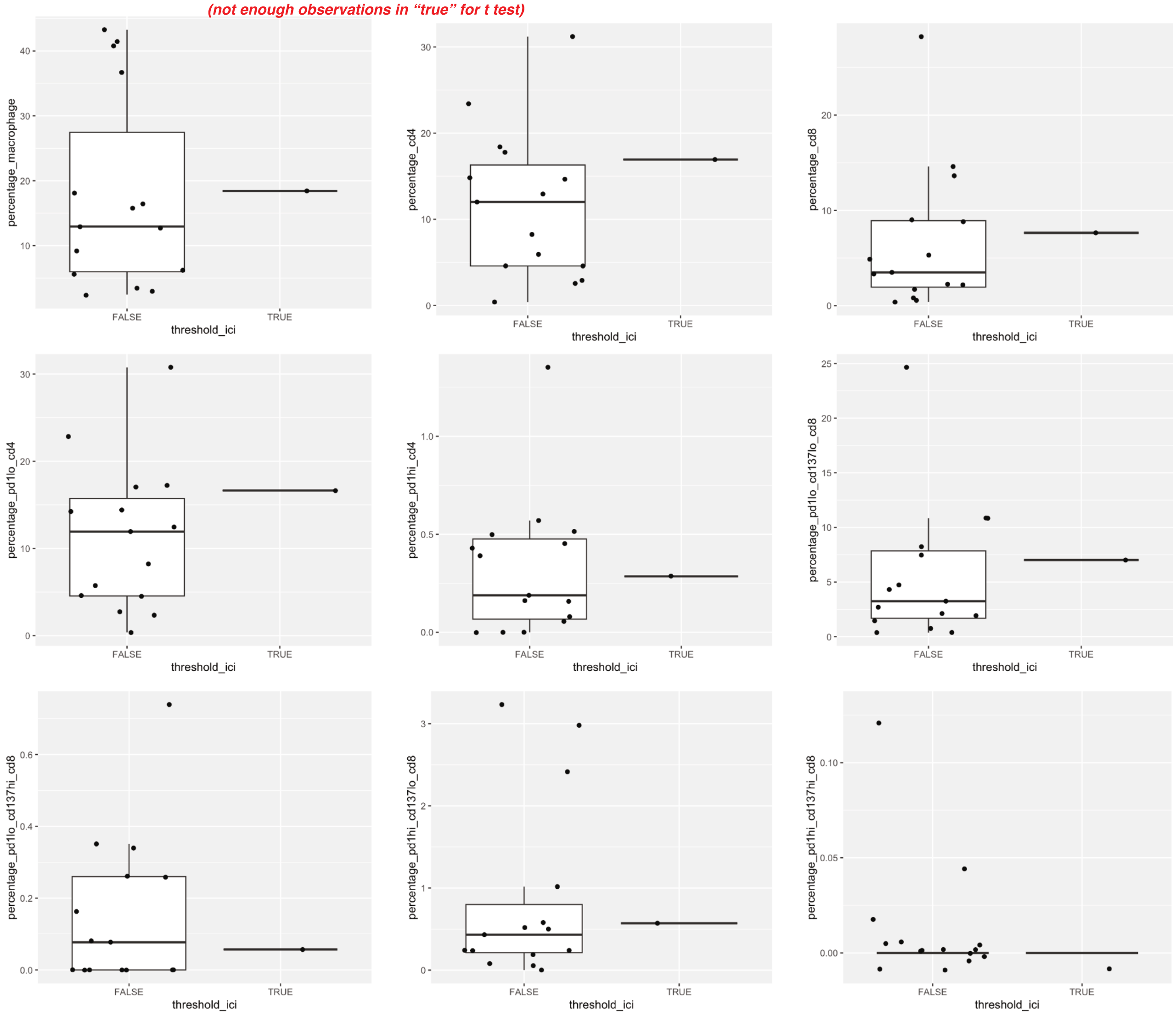

Figure S22. Immune cell abundances in ICI treatment, related to Figure 6

Immune population abundances in tissues, binned by whether the simulation with immune checkpoint inhibitor (ICI, Nivolumab) reached the tumor clearance threshold. Not enough tissues met the clearance threshold for significance test.

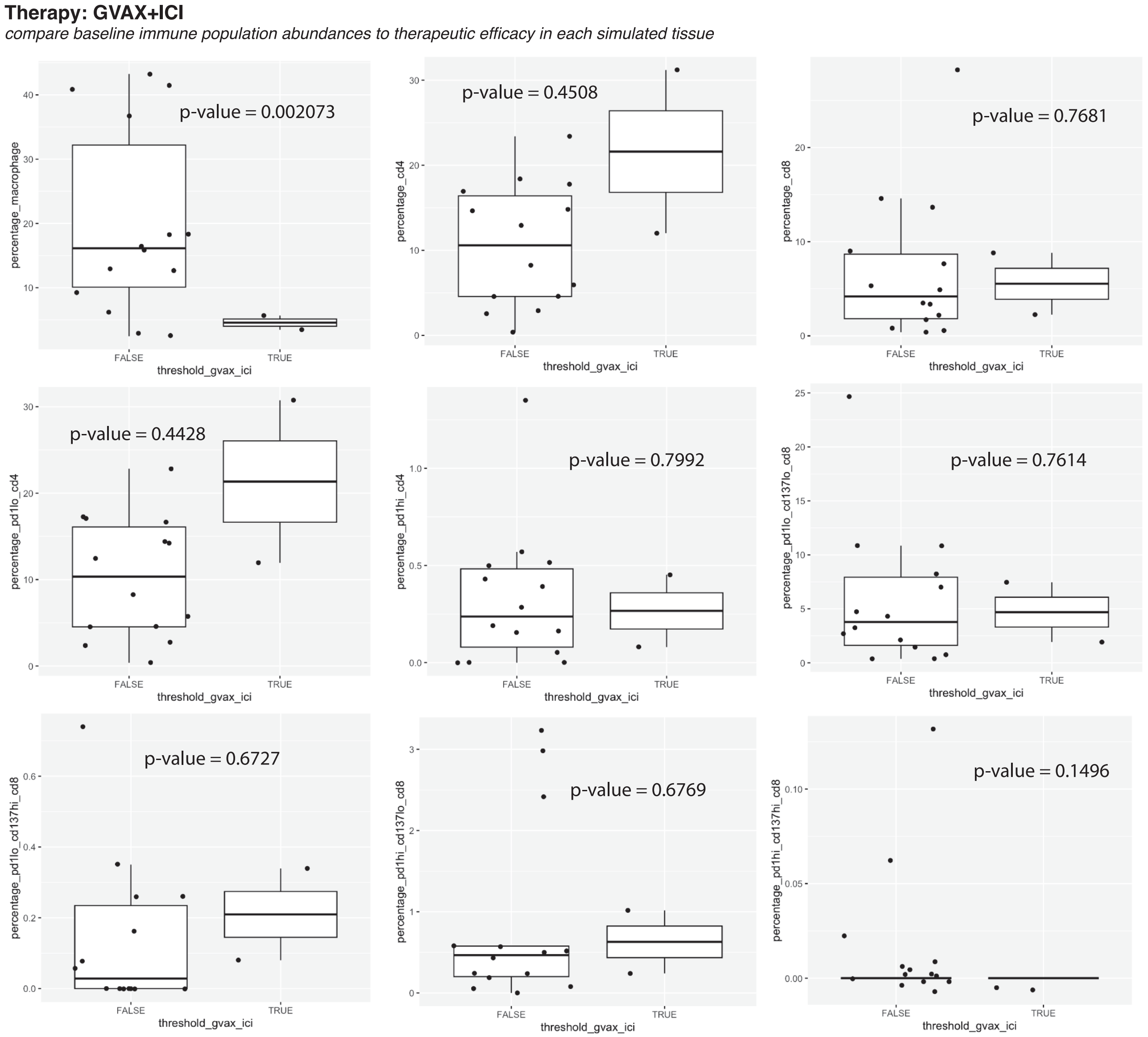

Figure S23. Immune cell abundances in GVAX + ICI treatment, related to Figure 6

Boxplots comparing baseline immune population abundances in each tissue simulation with efficacy of GVAX + ICI, contrasting tissues that grew relative to baseline vs. those that shrank (threshold =TRUE means the relative tumor volume change is <-0.5 or the tumor shrank; FALSE is the opposite, tumor growth did not meet our threshold).

Therapy: URU

compare baseline immune population abundances to therapeutic efficacy in each simulated tissue

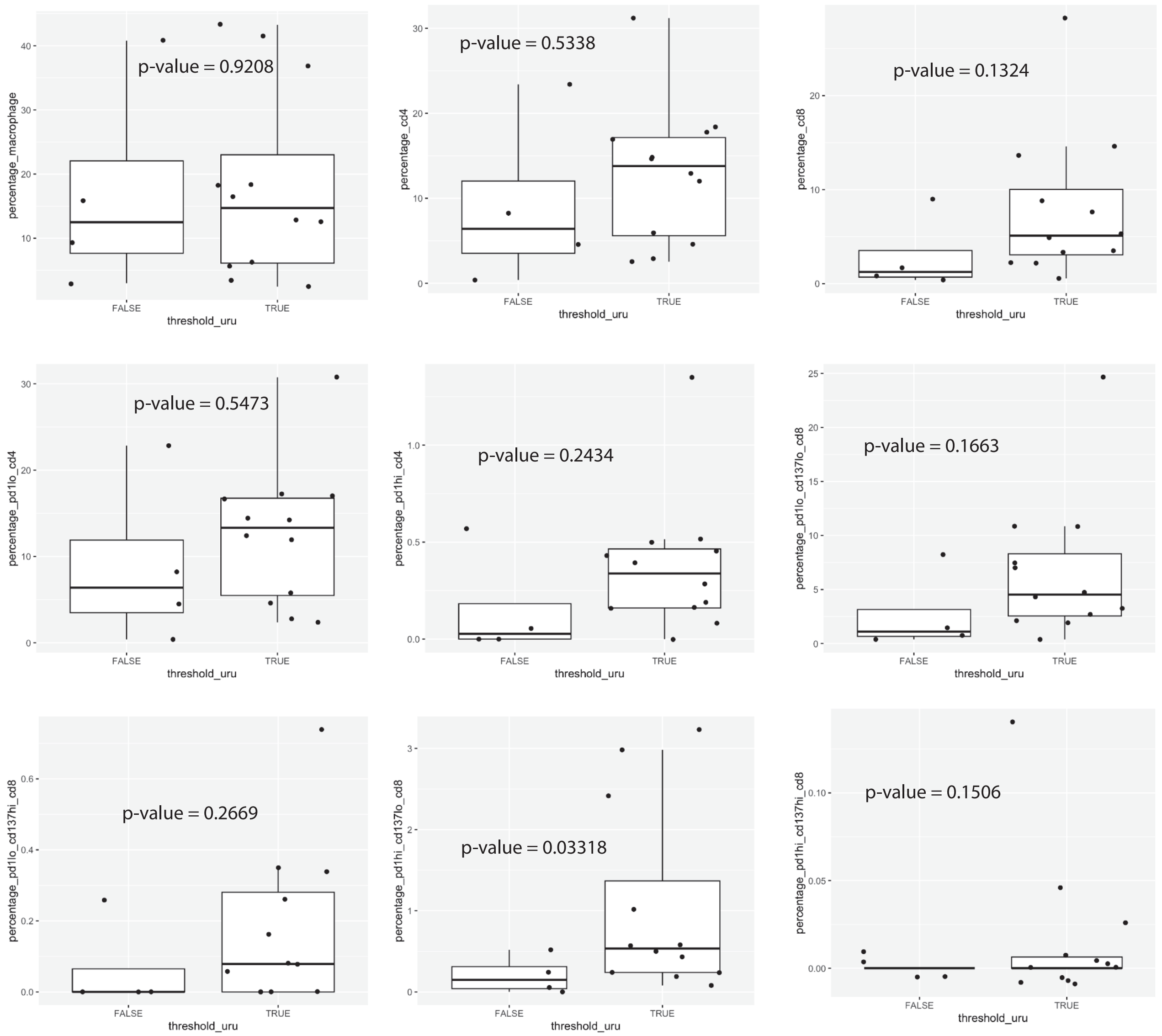

Figure S24. Immune cell abundances in URU treatment, related to Figure 6

Immune population abundances in tissues, binned by whether the simulation with CD137 agonist (Urelemab/URU) reached the tumor clearance threshold. None of these comparisons were significant.

CellPress
OPEN ACCESS

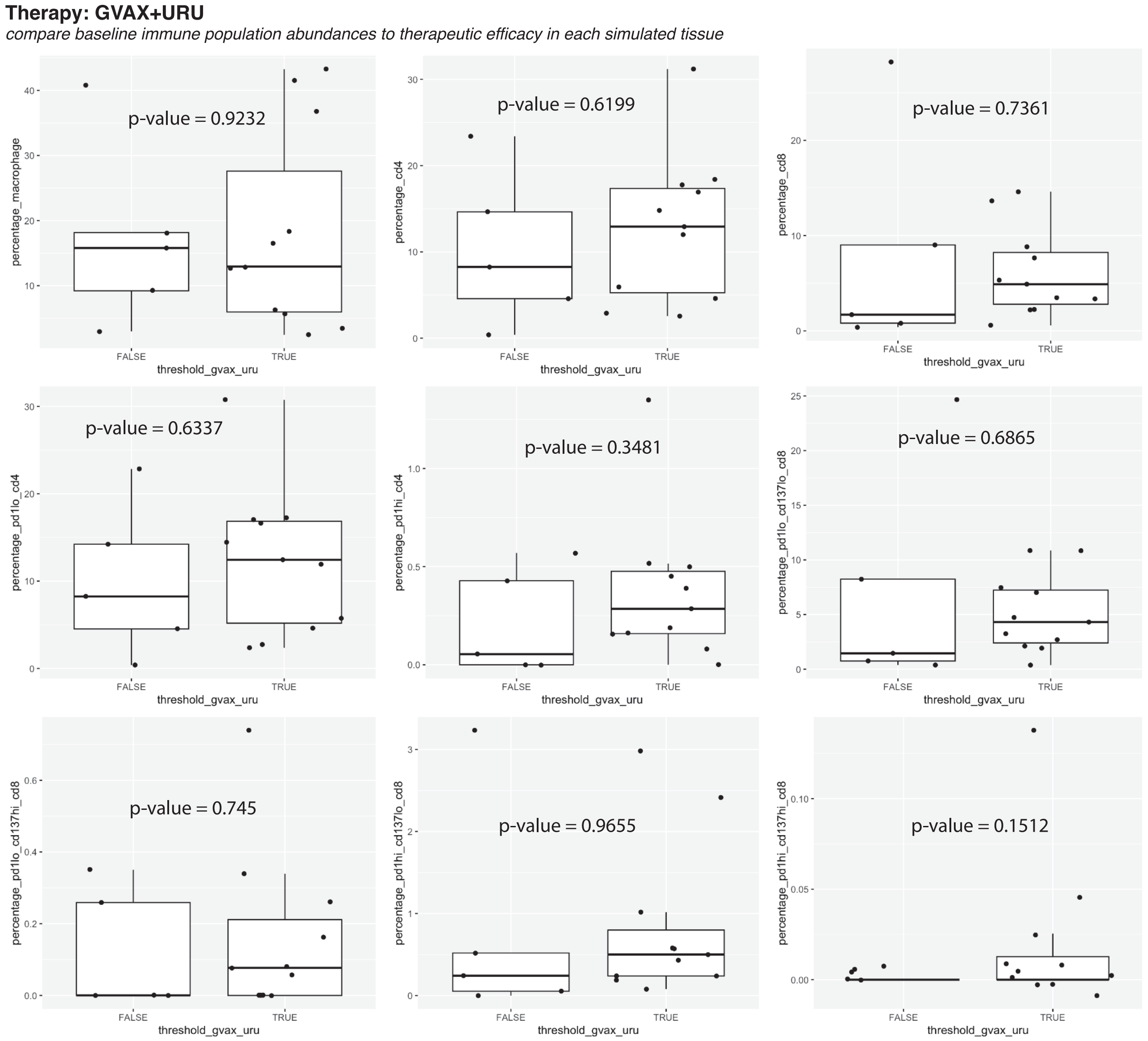

Figure S25. Immune cell abundances in GVAX + URU treatment, related to Figure 6

Immune population abundances in tissues, binned by whether the simulation with GVAX + URU reached the tumor clearance threshold. None of these comparisons were significant.

Therapy: ICI + URU
compare baseline immune population abundances to therapeutic efficacy in each simulated tissue

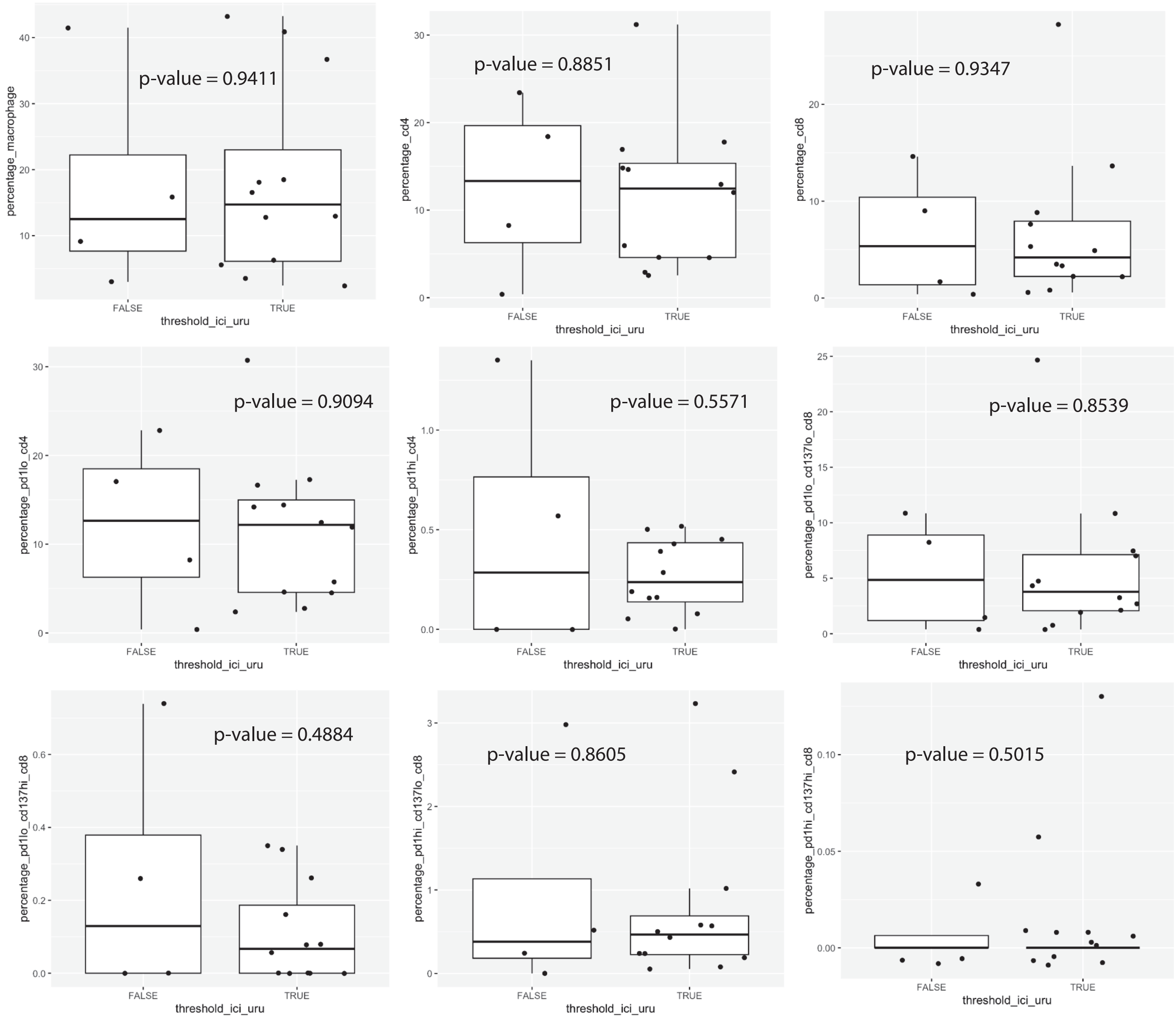

Figure S26. Immune cell abundances in ICI + URU treatment, related to Figure 6
Immune population abundances in tissues, binned by whether the simulation with ICI + URU reached the tumor clearance threshold. None of these comparisons were significant.
